# Synthesis of Multisubstituted Pyridines by Heterocyclization of TosMIC Derivatives: Total Synthesis of Caerulomycins A and K

**DOI:** 10.1021/acs.orglett.5c04454

**Published:** 2025-12-03

**Authors:** José A. García-García, José Luis Aceña, Patricia García-García, David Sucunza

**Affiliations:** Departamento de Química Orgánica y Química Inorgánica, Instituto de Investigación Química “Andrés M. del Río” (IQAR), 16720 Universidad de Alcalá, IRYCIS, 28805 Alcalá de Henares, Madrid, Spain

## Abstract

A concise synthesis of multisubstituted pyridines from α-allylic TosMIC derivatives and electrophiles is reported. The process involves a tandem heterocyclization exploiting the dual reactivity of the isocyanide group. The methodology tolerates various electrophiles, including halogen sources, and proceeds under mild conditions. Its utility is showcased in the total synthesis of caerulomycins A and K, in only five steps from TosMIC.

## Graphical abstract

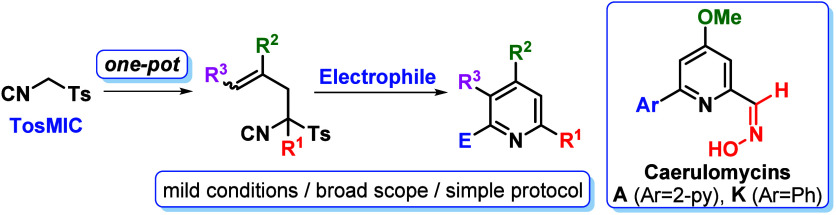

Pyridines represent a fundamental class of nitrogen-containing heterocycles that are widespread across natural products, pharmaceuticals and agrochemicals (Figure 1).^1^ Their structural versatility and prevalence, ranking as the most frequently appearing *N*-heterocycle in U.S. FDA-approved drugs (2013–2023), underscore their importance.^2^ Beyond their biological relevance, pyridines serve as key components in ligands, catalysts, and functional materials.^3^

**Figure 1:**
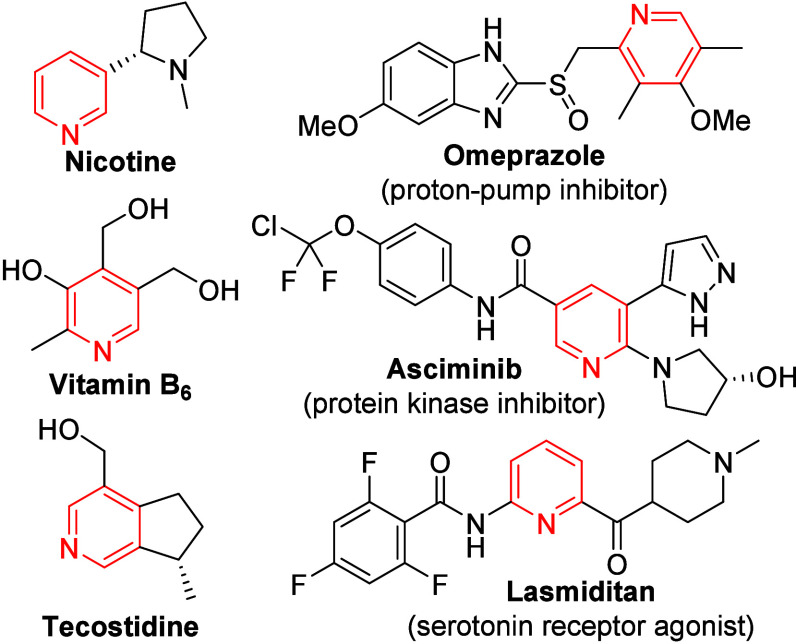
Examples of natural products (left) and drugs (right) based on the pyridine core.

Given their broad applicability, the development of synthetic methods for pyridine construction has been a longstanding focus.^4^ Classic multicomponent reactions, such as the Hantzsch, Kröhnke, and Bohlmann–Rahtz syntheses, provide efficient access to a variety of substituted pyridines.^5^ These methods, while foundational, often face limitations involving complex precursors, harsh conditions, or reduced functional group tolerance. Recent advances, including transition-metal-catalyzed cycloadditions and metal-free multicomponent processes, have expanded the toolkit for assembling highly functionalized pyridines.^6^ Nevertheless, challenges remain regarding the use of costly catalysts, functional group compatibility, and operational simplicity, factors that continue to drive the development of more practical and scalable synthetic routes.^7^

*p*-Tosylmethyl isocyanide (TosMIC) is a highly versatile and densely functionalized building block, featuring three reactive elements: an isocyanide group, acidic methylene protons, and a tosyl moiety.^8^ Traditionally employed in the synthesis of five-membered heterocycles, TosMIC has played a prominent role in heterocyclic chemistry.^9^ In recent years, our research has focused on expanding the synthetic utility of TosMIC to the construction of six-membered heterocycles.^10^ This effort has led to the development of new methodologies for the synthesis of isoquinolines^10c,10d^ and γ-carbolines,^10b^ through heterocyclization processes that take advantage of the capacity of isocyanides to act both as nucleophiles and electrophiles.

Herein, we report an efficient synthesis of multisubstituted pyridines using an approach related to the heterocyclization processes mentioned above. Moreover, to demonstrate its usefulness, we applied this new methodology to the synthesis of the natural products caerulomycins A^11^ and K,^12^ two members of a family of aromatic alkaloids possessing antimicrobial, immunosuppressive, and cytotoxic properties.^13^

First, synthesis of suitable TosMIC derivatives was a prerequisite for exploring our new approach. The target α-allylic α-alkyl compounds (**2a**–**2m**) were prepared via a single one-pot phase-transfer catalyzed (PTC) process. TosMIC reagent **1** reacted sequentially with two different alkyl halides in a biphasic system [CH_2_Cl_2_/NaOH (40%)] using tetrabutylammonium iodide (TBAI) as the phase-transfer catalyst. The initial step involved the addition of one equivalent of a simple alkyl halide at 0 °C, to suppress the formation of disubstituted byproducts. This was followed by the addition of substituted allylic bromides. The process furnished isonitriles **2a**–**2m** in good to excellent yields (Table 1).

**Table 1:**
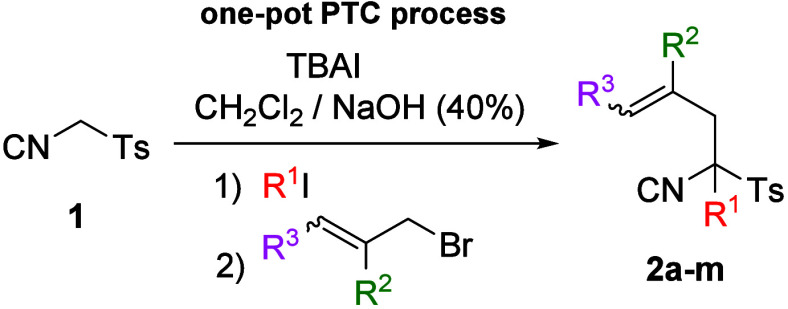
Synthesis of TosMIC Derivatives **2a**–**2m**

entry	R^1^	R^2^	R^3^	yield (%)	compd
1	Me	Ph	H	74	**2a**
2	Bu	Ph	H	78	**2b**
3	Ph	Ph	H	80^a^	**2c**
4	H	Ph	H	89^b^	**2d**
5	Me	4-MeO-C_6_H_4_	H	70	**2e**
6	Me	2-Br-C_6_H_4_	H	65	**2f**
7	Me	Ph	Me	89	**2g**
8	Me	Me	H	84	**2h**
9	Bn	Me	H	63	**2i**
10	Me	*t*-Bu	H	72	**2j**
11	Me	MeO	H	53	**2k**
12	Me	Br	H	51	**2l**
13	Me	H	H	79	**2m**

a Obtained in a single step process from α-phenyl TosMIC.

b Obtained in a single step process from TosMIC.

Our objective was to access pyridine derivatives via a heterocyclization process involving α-allylic TosMIC compounds **2** and various electrophiles. This transformation exploits the dual reactivity of isocyanides, which can act as both nucleophiles and electrophiles. Consequently, the cyclization would proceed through a tandem process in which alkene addition to the isocyanide group and the attack of this group on an electrophile occur simultaneously.

Based on our previous experience with heterocyclizations of TosMIC derivatives,^10b,10c^ we selected a carbonyl electrophile whose reactivity was enhanced by the addition of a Lewis acid, for our initial studies. Thus, α-allylic TosMIC **2a** was treated with acetone and AlEt_2_Cl. Various solvents and reagent equivalents were screened, with the best results obtained using 2 equiv of both reagents in CH_2_Cl_2_ (Table 2).

**Table 2:**
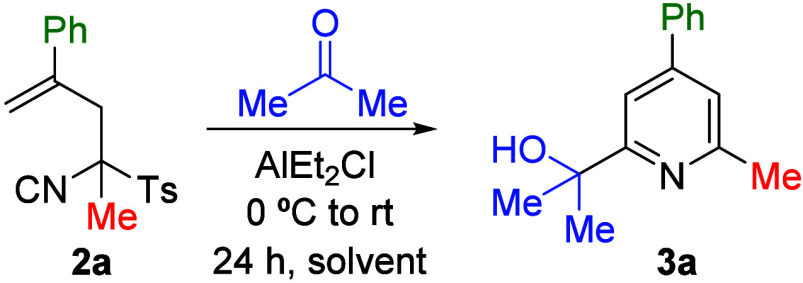
Optimization of the Reaction Conditions for **3a**

entry	solvent	equiv	yield (%)
1	CH_2_Cl_2_	2.0	76
2	CH_3_CN	2.0	68
3	Toluene	2.0	61
4	MeOH	2.0	0
5	CH_2_Cl_2_	1.1	60
6	CH_2_Cl_2_	5.0	31
7	CH_2_Cl_2_	2.0	54^a^

a Reaction at 0 °C.

Best conditions found for TosMIC derivative **2a** were applied to study the scope of this cyclization. A series of α-allylic TosMIC derivatives (**2a**–**2m**) was reacted with five different carbonyl compounds, including two aldehydes and three ketones of both aliphatic and aromatic types (Scheme 1). The corresponding pyridine products **3a**–**3o** were obtained in moderate to excellent yields (42–94%).

**Scheme 1:**
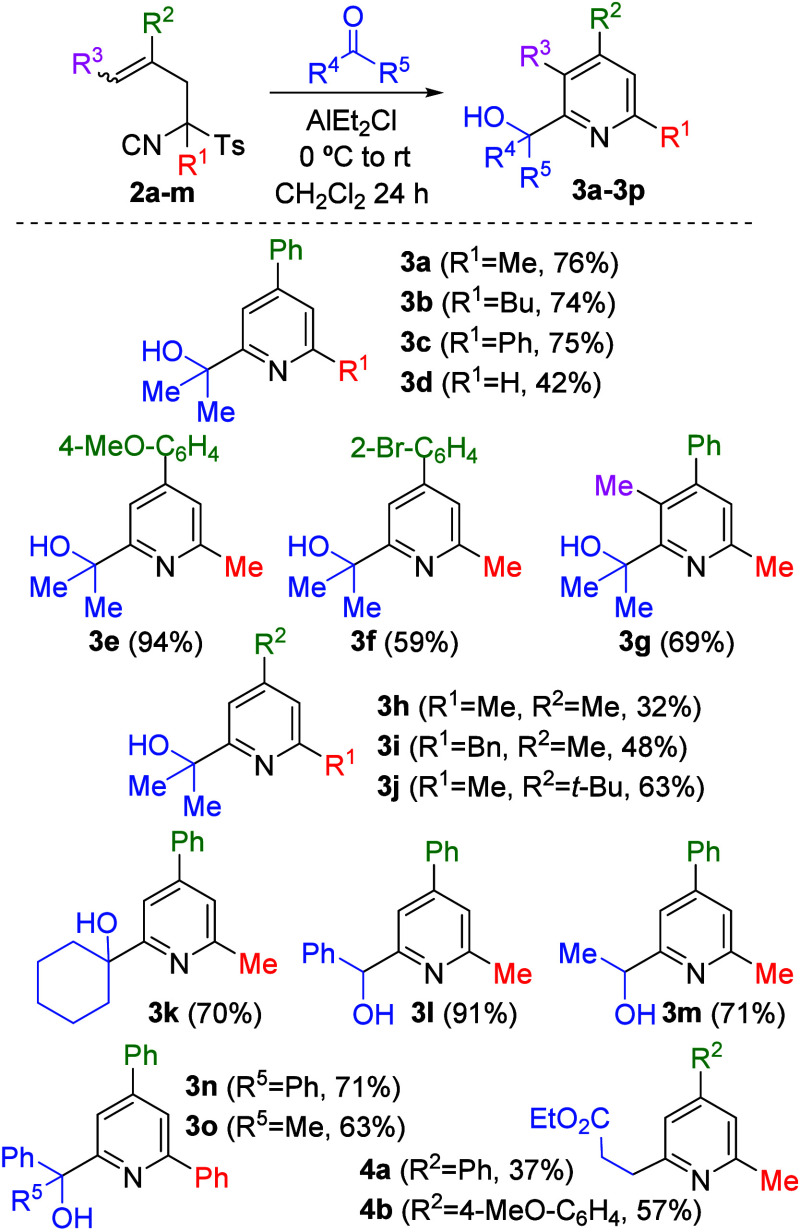
Synthesis of Pyridines **3a**–**3o** and **4a**–**4b**

Analysis of the reaction scope revealed that the substituent at the 4-position (R^2^) of the target pyridine plays a key role in determining the reaction yield. This trend can be rationalized by a mechanistic hypothesis (Scheme 2), which involves cyclization of the TosMIC derivative via electrophilic addition of the allylic alkene and nucleophilic attack of the isocyanide on the carbonyl compound in a single process. This transformation is proposed to proceed through a carbocationic intermediate, whose stability is modulated by the substituent at the 4-position. In general, aromatic substituents afford higher yields than aliphatic ones, presumably due to the formation of stabilized benzylic carbocations. Accordingly, the best results were obtained when a 4-MeO-C_6_H_4_ group was present (**3e**). In contrast, when the reaction was performed with a TosMIC derivative lacking substitution at this position, no cyclized product was detected, whereas the use of an aliphatic substituent capable of forming a tertiary carbocation led to only moderate yields (**3h**–**3j**). Derivative **2k** also failed to give any cyclized product, possibly due to its instability under acidic conditions. The mechanistic hypothesis concludes with an aromatization process involving elimination of the tosyl group and formation of the pyridine ring.

**Scheme 2:**
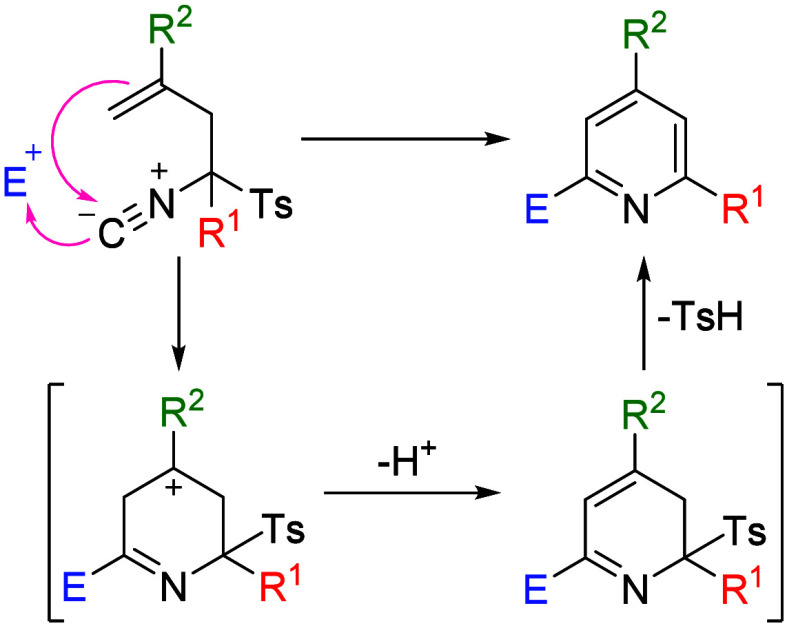
Mechanistic Hypothesis for the Heterocyclization

To further evaluate the scope of this new pyridine synthesis, we explored the heterocyclization of α-allylic TosMIC derivative **2a** using other types of electrophiles. The results were varied: no cyclization occurred with any electrophilic CF_3_ reagent tested,^14^ nor with propylene oxide, *N*-tosyl aziridine, or Eschenmoser’s salt in the presence of AlEt_2_Cl.^10b^ However, moderate yields were obtained when the Michael acceptor ethyl acrylate was employed instead of the carbonyl compounds under the same reaction conditions, although cyclization occurred only with the most reactive substrates—those bearing an aryl group at the 4-position of the pyridine ring (Scheme 1, compounds **4a** and **4b**). Acid-mediated cyclization was also investigated using both Brønsted and Lewis acids (Table 3). Thus, TosMIC derivative **2a** was treated with catalytic amounts of various acids, and only Sc­(OTf)_3_ was found to promote the formation of pyridine **5a** in good yield. Variation of the solvent and acid loading led to optimized conditions (CH_2_Cl_2_, 0.3 equiv of Sc­(OTf)_3_), which were subsequently applied to the synthesis of compounds **5b**–**5d** starting from precursors **2c**, **2i** and **2e**, respectively (Scheme 3). Once again, the substituent at the 4-position (R^2^) of the target pyridine was found to play a key role in determining the reaction yield, which ranged from 58% to 90% depending on the ability of the substituent at that position to stabilize the carbocationic intermediate.

**Table 3:**
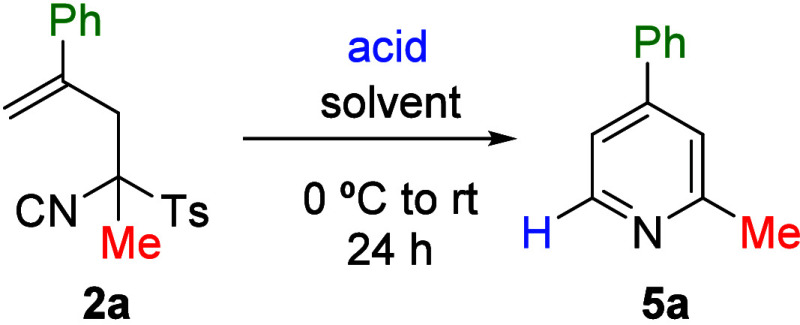
Optimization of the Reaction Conditions for **5a**

entry	acid	equiv	solvent	yield (%)
1	CF_3_CO_2_H^a^	0.3	CH_2_Cl_2_	0^b^
2	TsH	0.3	CH_2_Cl_2_	0^b^
3	AlCl_3_ ^a^	0.3	CH_2_Cl_2_	0^c^
4	BCl_3_	0.3	CH_2_Cl_2_	0^b^
5	Yb(OTf)_3_	0.3	CH_2_Cl_2_	37
6	Sc(OTf)_3_	0.3	CH_2_Cl_2_	70
7	Sc(OTf)_3_	0.3	CH_3_CN	38
8	Sc(OTf)_3_	0.3	MeOH	0^b^
9	Sc(OTf)_3_	0.3	HFIP	68
10	Sc(OTf)_3_	0.1	CH_2_Cl_2_	14
11	Sc(OTf)_3_	0.2	CH_2_Cl_2_	34
12	Sc(OTf)_3_	0.5	CH_2_Cl_2_	68

a Reaction performed at both 0 °C and rt.

b Decomposition.

c Only starting material recovered.

**Scheme 3:**
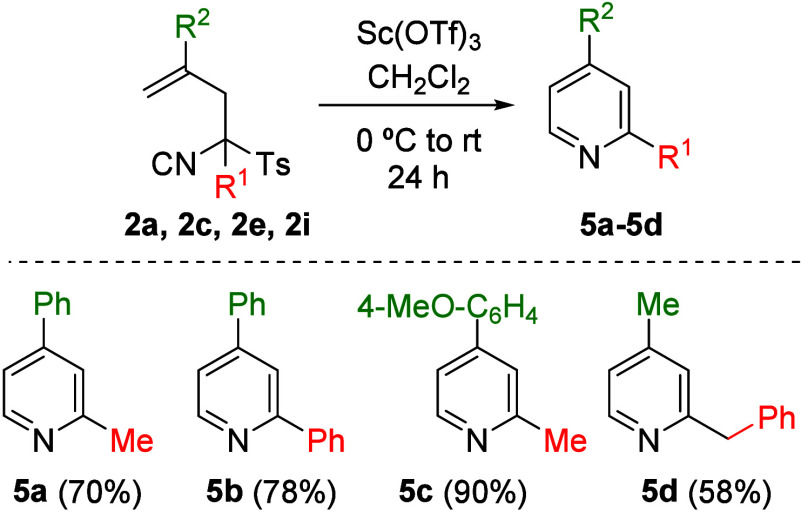
Synthesis of Pyridines **5a**–**5d**

Since all electrophiles used in the heterocyclization reaction so far involved a metal, an attempt was also made to perform the same type of transformation under metal-free conditions. For this purpose, TosMIC derivative **2a** was treated with various iodinating agents (*N*-iodophthalimide, 1,3-diiodo-5,5-di­methyl­hydan­toin, iodine and *N*-iodosuccinimide). Although all of them afforded the desired pyridine **6a** (Table 4), NIS gave the best result. Subsequent optimization of the solvent and the amount of reagent led to the final conditions used to study the scope of this transformation (Scheme 4). It is worth noting that in this case, the reaction performs better when CH_3_CN is used as the solvent.

**Table 4:**
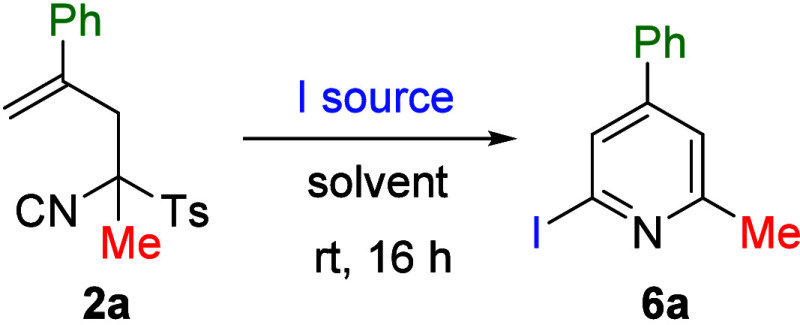
Optimization of the Reaction Conditions for **6a**

	I source	equiv	solvent	yield (%)
1	NIPht	2.0	CH_2_Cl_2_	65
2	DIH	2.0	CH_2_Cl_2_	22
3	I_2_	2.0	CH_2_Cl_2_	41
4	NIS	2.0	CH_2_Cl_2_	65
5	NIS	2.0	CH_3_CN	71
6	NIS	2.0	THF	40
7	NIS	2.0	Acetone	26
8	NIS	2.0	MeOH	7
9	NIS	1.1	CH_3_CN	23
10	NIS	3.0	CH_3_CN	48

**Scheme 4:**
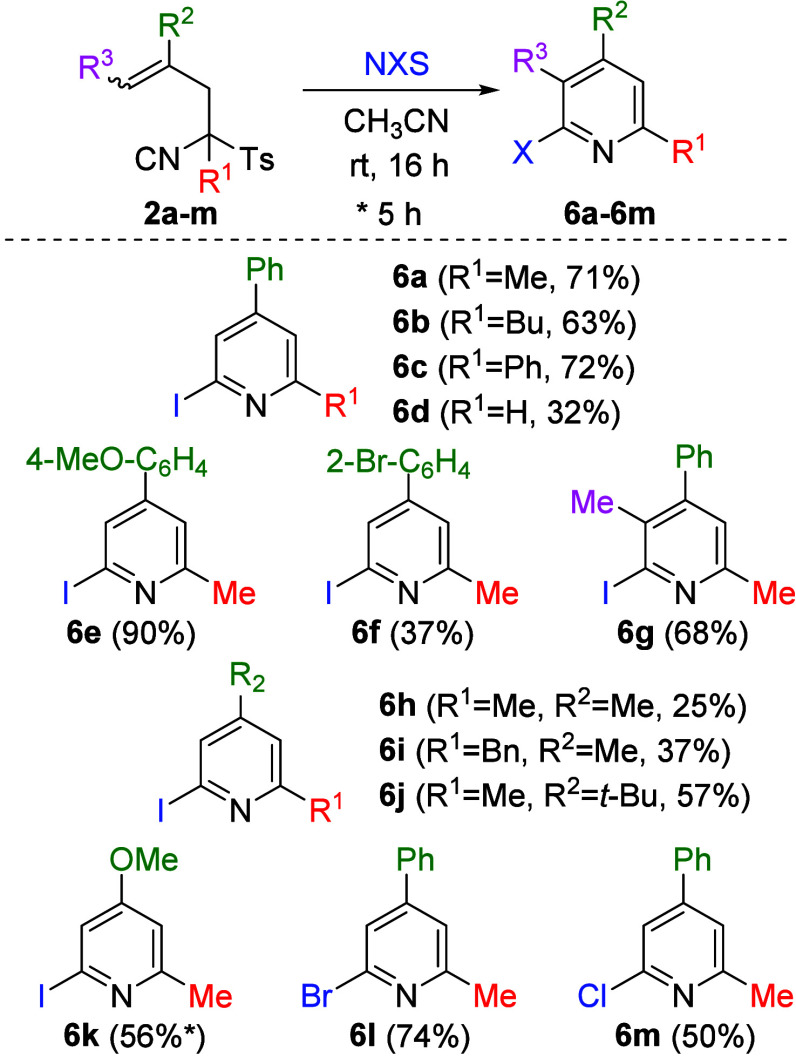
Synthesis of Pyridines **6a**–**6m**

The scope of this heterocyclization using *N*-iodosuccinimide as the electrophile follows the same reactivity pattern as the previous transformations. Once again, the substituent at the 4-position of the pyridine ring strongly influences the efficiency of the process. In general, aromatic substituents afford higher yields than aliphatic ones, with the best result observed for the 4-MeO-Ph group. Notably, the reaction proceeds with moderate yield when starting from TosMIC derivative **2k**, provided that the reaction time is reduced, affording pyridine **6k**. The cyclization process was also shown to occur with other halogens (Br, Cl), employing NBS or NCS instead of NIS, which led to the formation of the corresponding products **6l** and **6m** in moderate to good yields. In addition, a control experiment supporting the Scheme 2 mechanistic hypothesis showed that treating **2a** with 2 equiv of NIS and 1 equiv of the radical inhibitor 2,6-di-*tert*-butyl-4-methylphenol (BHT) provided **6a** in 49% yield.

Finally, the utility of this new methodology was showcased through the total synthesis of caerulomycins A and K, which can be accessed in just three steps from pyridine **6k** (Scheme 5). Both compounds share a closely related structure, differing only at the 2-position of the pyridine ring: caerulomycin A (**9**) contains a bipyridine motif, whereas caerulomycin K (**10**) features a phenyl group. As a result, the synthetic routes are analogous: a cross coupling reaction to introduce either the second pyridine ring or the phenyl moiety, followed by oxidation of the methyl group to an aldehyde using SeO_2_, and final oxime formation upon treatment with hydroxylamine. Regarding the coupling step, substitution of the iodine atom to install a second pyridine ring via a palladium-catalyzed Negishi coupling provided compound **7**, while a Suzuki reaction enabled the introduction of the phenyl group to yield compound **8**. Caerulomycin A had been previously synthesized from compound **7**,^11b^ whereas caerulomycin K was obtained in good yield by an analogous route, affording both natural products in only five steps from TosMIC.

**Scheme 5:**
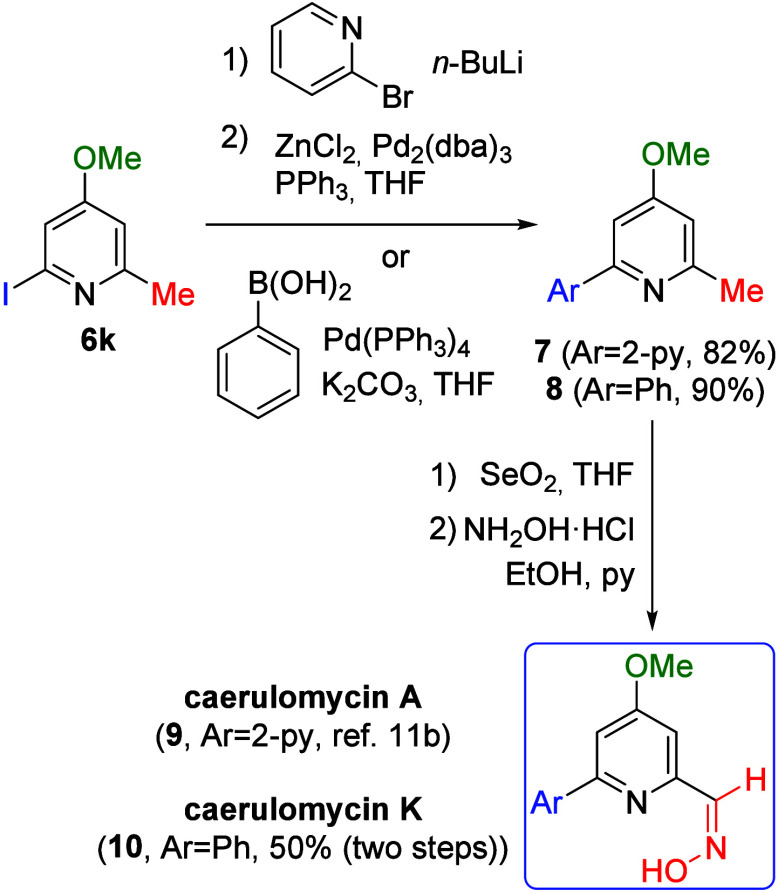
Total Synthesis of Caerulomycins A and K

In summary, we have developed a versatile and operationally simple method for the construction of highly functionalized pyridines via heterocyclization of α-allylic TosMIC derivatives with a variety of electrophiles. This transformation takes advantage of the ability of isocyanides to act both as nucleophiles and electrophiles. The methodology exhibits a broad substrate scope and good functional group tolerance, with reaction efficiency strongly influenced by the nature of the substituent at the 4-position of the pyridine ring. Notably, electrophilic halogenation using NIS enabled a transition-metal-free variant of the process, further expanding its synthetic utility. The synthetic value of the developed heterocyclization was highlighted by its application in the total synthesis of caerulomycins A and K, two biologically active natural products, which were accessed in only five steps from commercially available TosMIC.

## Supplementary Material



## Data Availability

The data underlying this study are available in the published article and its Supporting Information.

## Notes

Experimental procedures, characterization data, and ^1^H and ^13^C NMR spectra for new compounds (PDF)
