# Supplementary material for: Synthesis of Multisubstituted Pyridines by Heterocyclization of TosMIC Derivatives: Total Synthesis of Caerulomycins A and K
Source: Org Lett. 2025 Dec 3;28(3):907–11. doi: 10.1021/acs.orglett.5c04454 (PMC12836354; doi:10.1021/acs.orglett.5c04454)
Supplement: Supplementary file 1 [file ol5c04454_si_001.pdf]

Supporting Information

Synthesis of Multisubstituted Pyridines by Heterocyclization of TosMIC Derivatives: Total Synthesis of Caerulomycins A and K

José A. García-García, José Luis Aceña, Patricia García-García, and David Sucunza*

Departamento de Química Orgánica y Química Inorgánica, Instituto de Investigación Química "Andrés M. del Río" (IQAR), Universidad de Alcalá, IRYCIS, 28805, Alcalá de Henares, Madrid, Spain.

E-mail: david.sucunza@uah.es

Table of Contents

General Experimental Details -- S2

Experimental Procedures and Data -- S2

Copies of ^{1}H and ^{13}C spectra for novel compounds---------------------------------------S26

General Experimental Details

All reaction involving air sensitive compounds were carried out under inert atmosphere (Ar). Dry solvents, where necessary, were dried by a MBRAUN MB-SPS-800 apparatus. Starting materials sourced from commercial suppliers were used as received unless otherwise stated. Reaction mixtures were heated by means of an oil bath. Reactions were monitored using analytical TLC plates (Merck; silica gel 60 F254, 0.25 mm), and compounds were visualized with UV radiation. Silica gel grade 60 (70-230 mesh, Merck) was used for column chromatography. All melting points were determined in open capillary tubes on a Stuart Scientific SMP3 melting point apparatus (uncorrected). ^{1}H and ^{13}C NMR spectra were recorded on either a Varian Mercury VX-300, Varian Unity 500 MHz and Bruker Ascend 400 MHz spectrometer at room temperature. Chemical shifts are given in ppm (δ) downfield from tetramethylsilane, with calibration on the residual protio-solvent used (δ_H = 7.26 ppm and δ_C = 77.2 ppm for $CDCl_3$). Coupling constants (*J*) are in Hertz (Hz) and signals are described as follows: s, singlet; d, doublet; t, triplet; q, quadruplet; bs, broad singlet; dd, double doublet; apt, apparent triplet; dq, double quadruplet; ddd, double doublet of doublets and m, multiplet. High-resolution analysis (HRMS) were performed on an Agilent 6210 time of-flight LC/MS.

General procedure for the synthesis of α-allyl-α-alkyl TosMIC derivatives 2a-2m

To a cold (0 ºC), stirred solution of alkyl halide (1.1 equiv), the corresponding TosMIC reagent (1.0 equiv) and tetrabutylammonium iodide (0.2 equiv) in CH_2Cl_2 (0.5 M), a solution of NaOH (40% in water, 20 equiv) was added, and the reaction mixture was vigorously stirred at the same temperature for 2 h. Then the corresponding allyl bromide (1.5 equiv) in CH_2Cl_2 (0.5 M) and additional NaOH (40% in water, 20 equiv) were added, and the mixture was warmed up to room temperature and stirred for 18 h. Water was added, and the two layers were separated. The aqueous layer was extracted with CH_2Cl_2, and the combined organic layers were dried over anhydrous $MgSO_4$, filtered and purified by flash chromatography, supplying pure product.

1-((2-Isocyano-4-phenylpent-4-en-2-yl)sulfonyl)-4-methylbenzene (2a)

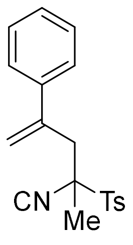

2a

Following the general procedure outlined above, from TosMIC (1.0 g, 5.12 mmol), methyl iodide (0.35 mL, 0.8 g, 5.63 mmol) and (3-bromoprop-1-en-2-yl)benzene (1.51 g, 7.68 mmol) in 10 mL of CH_2Cl_2, compound **2a** was obtained as a light yellow solid (1.24

g, 3.81 mmol, 74%). R$_f$ = 0.5 (Hexane:EtOAc, 4:1). Mp = 90–92 ºC. **^{1}H NMR (400 MHz, CDCl$_3$)** δ 7.89 (d, *J* = 8.3 Hz, 2H), 7.43 (d, *J* = 8.0 Hz, 2H), 7.39–7.27 (m, 5H), 5.55 (s, 1H), 5.31 (s, 1H), 3.30 (d, *J* = 13.7 Hz, 1H), 3.13 (d, *J* = 13.7 Hz, 1H), 2.49 (s, 3H), 1.41 (s, 3H). **^{13}C NMR (101 MHz, CDCl$_3$)** δ 164.5, 146.7, 140.6(2C), 131.6, 130.1, 129.3, 128.8, 128.3, 126.5, 120.9, 78.8, 38.3, 22.0, 21.1. **HRMS (ESI-TOF) m/z**: Calcd for $C_{19}H_{20}NO_2S$ [M+H]$^+$: 326.1209; found: 326.1209.

1-((4-Isocyano-2-phenyloct-1-en-4-yl)sulfonyl)-4-methylbenzene (2b)

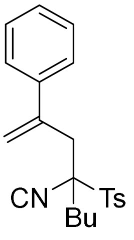

2b

Following the general procedure outlined above, from TosMIC (0.15 g, 0.77 mmol), butyl iodide (0.1 mL, 0.155 g, 0.85 mmol) and (3-bromoprop-1-en-2-yl)benzene (0.28 g, 1.15 mmol) in 1.5 mL of CH_2Cl_2, compound **2b** was obtained as a light yellow solid (0.22 g, 0.6 mmol, 78%). R$_f$ = 0.6 (Hexane:EtOAc, 4:1). Mp = 80–82 ºC. **^{1}H NMR (400 MHz, CDCl$_3$)** δ 7.89 (d, *J* = 8.3 Hz, 2H), 7.42 (d, *J* = 8.0 Hz, 2H), 7.35–7.26 (m, 5H), 5.48 (s, 1H), 5.29 (s, 1H), 3.31 (d, *J* = 13.9 Hz, 1H), 3.00 (d, *J* = 13.9 Hz, 1H), 2.50 (s, 3H), 1.86–1.75 (m, 2H), 1.48–1.33 (m, 1H), 1.32–1.17 (m, 1H), 0.99–0.88 (m, 2H), 0.68 (t, *J* = 7.3 Hz, 3H). **^{13}C NMR (101 MHz, CDCl$_3$)** δ 164.8, 146.4, 140.8, 140.8, 131.3, 130.3, 129.9, 128.5, 128.1, 126.6, 120.9, 81.9, 38.0, 32.9, 26.1, 22.4, 21.8, 13.4. **HRMS (ESI-TOF) m/z**: Calcd for $C_{22}H_{26}NO_2S$ [M+H]$^+$: 368.1679; found: 368.1679.

(1-Isocyano-1-tosylbut-3-ene-1,3-diyl)dibenzene (2c)

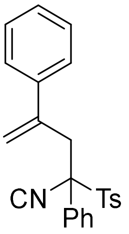

2c

Following the general procedure outlined above, from α-phenyl TosMIC (0.38 g, 1.4 mmol) and (3-bromoprop-1-en-2-yl)benzene (0.55 g, 2.8 mmol) in 3 mL of CH_2Cl_2, compound **2c** was obtained as a light yellow solid (0.43 g, 1.12 mmol, 80%). R$_f$ = 0.65 (Hexane:EtOAc, 4:1). Mp = 90–92ºC. **^{1}H NMR (400 MHz, CDCl$_3$)** δ 7.33 (d, *J* = 8.0 Hz, 2H), 7.28–7.19 (m, 3H), 7.17–7.02 (m, 9H), 5.26 (s, 1H), 5.13 (d, *J* = 1.2 Hz, 1H), 3.93 (d, *J* = 14.8 Hz, 1H), 3.73 (d, *J* = 13.5 Hz, 1H), 2.37 (s, 3H). **^{13}C NMR (101 MHz,**

$CDCl_3$) δ 166.9, 146.1, 141.1, 140.8, 131.2, 129.6, 129.2, 129.1, 128.6, 128.1, 128.0, 127.8, 127.5, 126.7, 120.1, 85.7, 37.9, 21.7. **HRMS (ESI-TOF) m/z**: Calcd for $C_{24}H_{22}NO_2S$ $[M+H]^+$: 388.1366; found: 388.1366.

1-((1-Isocyano-3-phenylbut-3-en-1-yl)sulfonyl)-4-methylbenzene (2d)

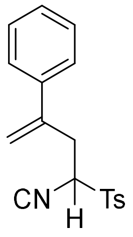

2d

Following the general procedure outlined above, from TosMIC (0.2 g, 1.02 mmol) and (3-bromoprop-1-en-2-yl)benzene (0.22 g, 1.13 mmol) in 2 mL of CH_2Cl_2, compound **2d** was obtained as a brown oil (0.25 g, 0.82 mmol, 89%). R_f = 0.48 (Hexane:EtOAc, 4:1). **^{1}H NMR (400 MHz, $CDCl_3$)** δ 7.86 (d, J = 8.4 Hz, 2H), 7.42 (d, J = 8.1 Hz, 2H), 7.39–7.33 (m, 5H), 5.53 (s, 1H), 5.31 (s, 1H), 4.45 (dd, J = 11.5, 2.8 Hz, 1H), 3.63 (d, J = 14.4 Hz, 1H), 2.81 (dd, J = 14.3, 11.5 Hz, 1H), 2.48 (s, 3H). **^{13}C NMR (101 MHz, $CDCl_3$)** δ 165.2, 146.8, 140.2, 137.9, 131.1, 130.2, 130.1, 129.0, 128.5, 126.2, 118.6, 71.5, 35.1, 21.8. **HRMS (ESI-TOF) m/z**: Calcd for $C_{18}H_{18}NO_2S$ $[M+H]^+$: 312.1053; found: 312.1054.

1-((2-Isocyano-4-(4-methoxyphenyl)pent-4-en-2-yl)sulfonyl)-4-methylbenzene (2e)

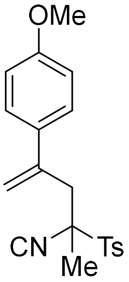

2e

Following the general procedure outlined above, from TosMIC (0.15 g, 0.79 mmol), methyl iodide (0.05 mL, 0.122 g, 0.87 mmol) and 1-(3-bromoprop-1-en-2-yl)-4-methoxybenzene (0.27 g, 1.19 mmol) in 1.6 mL of CH_2Cl_2, compound **2e** was obtained as a yellow oil (0.197 g, 0.554 mmol, 70%). R_f = 0.32 (Hexane:EtOAc, 4:1). **^{1}H NMR (400 MHz, $CDCl_3$)** δ 7.89 (d, J = 8.4 Hz, 2H), 7.46–7.39 (m, 2H), 7.35–7.26 (m, 2H), 6.86 (d, J = 8.8 Hz, 2H), 5.47 (d, J = 1.0 Hz, 1H), 5.21 (d, J = 1.0 Hz, 1H), 3.80 (s, 3H), 3.26 (d, J = 13.7 Hz, 1H), 3.09 (d, J = 13.7 Hz, 1H), 2.49 (s, 3H), 1.42 (s, 3H). **^{13}C NMR (101 MHz, $CDCl_3$)** δ 164.3, 159.6, 146.5, 139.8, 132.8, 131.4, 129.9, 129.2, 127.5, 119.1,

114.0, 78.8, 55.3, 38.1, 21.8, 21.0. **HRMS (ESI-TOF) m/z**: Calcd for $C_{13}H_{16}NO$ [M+H]+[-$C_7H_8SO_2$]: 201.1154; found: 201.1102.

1-Bromo-2-(4-isocyano-4-tosylpent-1-en-2-yl)benzene (2f)

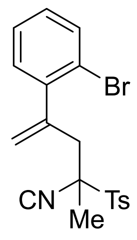

2f

Following the general procedure outlined above, from TosMIC (0.06 g, 0.29 mmol), methyl iodide (0.02 mL, 0.045 g, 0.32 mmol) and 1-bromo-2-(3-bromoprop-1-en-2-yl)benzene (0.095 g, 0.34 mmol) in 0.6 mL of CH_2Cl_2, compound **2f** was obtained as a yellow oil (0.075 g, 0.19 mmol, 65%). R_f = 0.51 (Hexane:EtOAc, 4:1). **^{1}H NMR (400 MHz, $CDCl_3$)** δ 7.86 (d, J = 8.3 Hz, 2H), 7.55 (dd, J = 8.0, 1.2 Hz, 1H), 7.41 (d, J = 8.1 Hz, 2H), 7.34–7.20 (m, 2H), 7.15 (ddd, J = 8.0, 6.8, 2.4 Hz, 1H), 5.51 (d, J = 1.2 Hz, 1H), 5.39 (d, J = 1.1 Hz, 1H), 3.25 (d, J = 2.6 Hz, 2H), 2.48 (s, 3H), 1.54 (s, 3H). **^{13}C NMR (101 MHz, $CDCl_3$)** δ 164.7, 146.6, 141.8, 140.9, 133.3, 131.4, 130.9, 129.9, 129.4, 128.9, 127.6, 124.5, 121.8, 78.2, 39.8, 21.8, 20.5. **HRMS (ESI-TOF) m/z**: Calcd for $C_{19}H_{18}BrNO_2S$ $[M]^+$: 403.0242; found: 403.0234.

1-((2-Isocyano-4-phenylhex-4-en-2-yl)sulfonyl)-4-methylbenzene (2g)

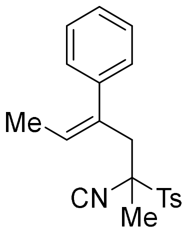

2g

Following the general procedure outlined above, from TosMIC (0.15 g, 0.76 mmol), methyl iodide (0.05 mL, 0.12 g, 0.32 mmol) and (1-bromobut-2-en-2-yl)benzene (0.19 g, 0.91 mmol) in 1.5 mL of CH_2Cl_2, compound **2g** was obtained as a light yellow solid (0.23 g, 0.76 mmol, 89%). R_f = 0.54 (Hexane:EtOAc, 4:1). Mp = 83–85ºC. **^{1}H NMR (400 MHz, $CDCl_3$)** δ 7.90 (d, J = 8.4 Hz, 2H), 7.42 (d, J = 7.7 Hz, 2H), 7.35–7.20 (m, 5H), 6.03 (q, J = 7.1 Hz, 1H), 3.44 (d, J = 14.0 Hz, 1H), 3.19 (d, J = 14.0 Hz, 1H), 2.49 (s, 3H), 1.80 (d, J = 7.0 Hz, 3H), 1.35 (s, 3H). **^{13}C NMR (101 MHz, $CDCl_3$)** δ 164.8, 146.5, 142.9, 132.9, 131.5(2C), 129.9, 129.2, 128.6, 127.4, 126.6, 78.5, 32.2, 21.8, 21.5, 15.2. **HRMS (ESI-TOF) m/z**: Calcd for $C_{20}H_{22}NO_2S$ $[M+H]^+$: 340.1366; found: 340.1366.

1-((2-Isocyano-4-methylpent-4-en-2-yl)sulfonyl)-4-methylbenzene (2h)

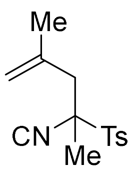

2h

Following the general procedure outlined above, from TosMIC (0.75 g, 3.82 mmol), methyl iodide (0.26 mL, 0.6 g, 4.2 mmol) and 3-bromo-2-methylprop-1-ene (0.77 g, 5.7 mmol) in 7.6 mL of CH_2Cl_2, compound **2h** was obtained as a light yellow solid (0.85 g, 3.23 mmol, 84%). R_f = 0.34 (Hexane:EtOAc, 4:1). Mp = 71–73°C. **^{1}H NMR (400 MHz, $CDCl_3$)** δ 7.89 (d, *J* = 8.3 Hz, 2H), 7.43 (dd, *J* = 31.8, 8.1 Hz, 2H), 5.09 (p, *J* = 1.6 Hz, 1H), 4.93 (q, *J* = 0.9 Hz, 1H), 2.76–2.63 (m, 2H), 2.50 (s, 3H), 1.87 (s, 3H), 1.66 (s, 3H). **^{13}C NMR (101 MHz, $CDCl_3$)** δ 164.8, 146.7, 137.4, 131.6, 130.1, 129.3, 119.0, 78.0, 41.2, 23.9, 22.0, 20.5. **HRMS (ESI-TOF) m/z**: Calcd for $C_{14}H_{18}NO_2S$ $[M+H]^+$: 264.1053; found: 264.1055.

1-((2-Isocyano-4-methyl-1-phenylpent-4-en-2-yl)sulfonyl)-4-methylbenzene (2i)

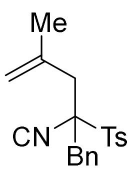

2i

Following the general procedure outlined above, from TosMIC (0.5 g, 2.56 mmol), benzyl bromide (0.33 mL, 0.48 g, 2.82 mmol) and 3-bromo-2-methylprop-1-ene (0.52 g, 3.84 mmol) in 5 mL of CH_2Cl_2, compound **2i** was obtained as a light yellow solid (0.55 g, 1.62 mmol, 63%). R_f = 0.42 (Hexane:EtOAc, 4:1). Mp = 79–81°C**. ^{1}H NMR (400 MHz, $CDCl_3$)** δ 7.88 (d, *J* = 8.4 Hz, 2H), 7.40 (d, *J* = 8.1 Hz, 2H), 7.30 (q, *J* = 2.9 Hz, 5H), 4.96 (p, *J* = 1.6 Hz, 1H), 4.81–4.76 (m, 1H), 3.24 (s, 2H), 2.75–2.61 (m, 2H), 2.48 (s, 3H), 1.65 (t, *J* = 1.1 Hz, 3H). **^{13}C NMR (101 MHz, $CDCl_3$)** δ 166.7, 146.4, 138.1, 132.4, 131.5, 131.1, 130.5, 129.8, 128.4, 128.0, 118.8, 80.8, 41.6, 40.0, 23.2, 21.8. **HRMS (ESI-TOF) m/z**: Calcd for $C_{20}H_{22}NO_2S$ $[M+H]^+$: 340.1366; found: 340.1367.

1-((2-Isocyano-5,5-dimethyl-4-methylenehexan-2-yl)sulfonyl)-4-methylbenzene (2j)

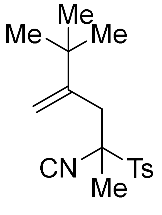

2j

Following the general procedure outlined above, from TosMIC (0.093 g, 0.48 mmol), methyl iodide (0.03 mL, 0.075 g 0.53 mmol) and 2-(bromomethyl)-3,3-dimethylbut-1-ene (0.13 g, 0.72 mmol) in 1 mL of CH_2Cl_2, compound **2j** was obtained as a light yellow oil (0.105 g, 0.34 mmol, 72%). R_f = 0.69 (Hexane:EtOAc, 4:1). **^{1}H NMR (400 MHz, $CDCl_3$)** δ 7.90 (d, J = 8.4 Hz, 2H), 7.43 (d, J = 8.1 Hz, 2H), 5.19 (d, J = 3.3 Hz, 2H), 2.77 (s, 2H), 2.49 (s, 3H), 1.70 (s, 3H), 1.06 (s, 9H). **^{13}C NMR (75 MHz, $CDCl_3$)** δ 164.8, 149.2, 146.5, 131.6, 129.9, 129.2, 111.8, 78.8, 36.9, 32.6, 28.9, 21.8, 20.2. **HRMS (ESI-TOF) m/z**: Calcd for $C_{17}H_{24}NO_2S$ $[M+H]^+$: 306.1522; found: 306.1522.

1-((2-Isocyano-4-methoxypent-4-en-2-yl)sulfonyl)-4-methylbenzene (2k)

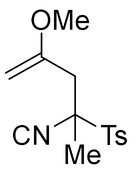

2k

Following the general procedure outlined above, from TosMIC (0.432 g, 2.21 mmol), methyl iodide (0.15 mL, 0.35 g 2.43 mmol) and freshly prepared 2-methoxylallyl bromide (0.467 g, 3.1 mmol) in 4.5 mL of CH_2Cl_2, compound **2k** was obtained as a brown solid (0.327 g, 1.17 mmol, 53%). R_f = 0.49 (Hexane:EtOAc, 4:1). Mp = 92–94°C**.** **^{1}H NMR (400 MHz, $CDCl_3$)** δ 7.90 (d, J = 8.4 Hz, 2H), 7.43 (d, J = 8.1 Hz, 2H), 5.45 (s, 1H), 5.29 (s, 1H), 3.58 (s, 3H), 3.10 (s, 2H), 2.48 (s, 3H), 1.77 (s, 3H). **^{13}C NMR (101 MHz, $CDCl_3$)** δ 164.9, 153.6, 146.7, 131.5, 130.0, 128.9, 91.8, 83.2, 55.7, 35.9, 21.8, 20.8. **HRMS (ESI-TOF) m/z**: Calcd for $C_{14}H_{18}NO_3S$ $[M+H]^+$: 280.1002; found: 280.1002.

1-((4-Bromo-2-isocyanopent-4-en-2-yl)sulfonyl)-4-methylbenzene (2l)

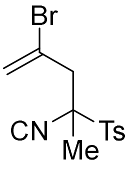

2l

Following the general procedure outlined above, from TosMIC (0.23 g, 1.19 mmol), methyl iodide (0.08 mL, 0.19 g 1.31 mmol) and 2,3-dibromoprop-1-ene (0.185 mL, 0.36 g, 1.79 mmol) in 2.5 mL of CH_2Cl_2, compound **2l** was obtained as a brown solid (0.201 g, 0.61 mmol, 51%). R_f = 0.47 (Hexane:EtOAc, 4:1). Mp = 75-77ºC. **^{1}H NMR (400 MHz, $CDCl_3$)** δ 7.82 (d, J = 8.4 Hz, 2H), 7.38 (d, J = 7.8 Hz, 2H), 5.82–5.77 (m, 1H), 5.73 (d, J = 2.1 Hz, 1H), 3.10–3.04 (m, 2H), 2.43 (s, 3H), 1.74 (s, 3H). **^{13}C NMR (101 MHz, $CDCl_3$)** δ 165.5, 146.9, 131.5, 130.1, 128.7, 125.1, 122.2, 77.8, 44.1, 21.9, 20.5. **HRMS (ESI-TOF) m/z**: Calcd for $C_{13}H_{15}BrNO_2S$ $[M+H]^+$: 328.0001; found: 328.0001.

1-((2-Isocyanopent-4-en-2-yl)sulfonyl)-4-methylbenzene (2m)

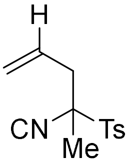

2m

Following the general procedure outlined above, from TosMIC (0.25 g, 1.28 mmol), methyl iodide (0.09 mL, 0.2 g 1.41 mmol) and 3-bromoprop-1-ene (0.166 mL, 0.23 g, 1.92 mmol) in 2.6 mL of CH_2Cl_2, compound **2m** was obtained as a light yellow solid (0.25 g, 0.1 mmol, 79%). R_f = 0.62 (Hexane:EtOAc, 4:1). Mp = 78–80ºC. **^{1}H NMR (400 MHz, $CDCl_3$)** δ 7.87 (d, J = 8.4 Hz, 2H), 7.42 (d, J = 8.2 Hz, 2H), 5.82–5.70 (m, 1H), 5.36–5.22 (m, 2H), 2.74 (dd, J = 13.9, 6.4 Hz, 1H), 2.70–2.62 (m, 1H), 2.48 (s, 3H), 1.63 (s, 3H).**^{13}C NMR (101 MHz, $CDCl_3$)** δ 164.0, 146.6, 131.3, 130.0, 129.3, 128.6, 122.5, 77.9, 38.4, 21.8, 20.4. **HRMS (ESI-TOF) m/z**: Calcd for $C_{13}H_{16}NO_2S$ $[M+H]^+$: 250.0896; found: 250.0896.

General procedure for the synthesis of pyridines 3a-3o

To a cold (0 °C), stirred solution of the corresponding α-allyl-α-alkyl TosMIC derivative **2** (1.0 equiv) and the corresponding aldehyde or ketone (2.0 equiv) in dry CH_2Cl_2 (0.1 M), under argon atmosphere, a solution of $AlEt_2Cl$ (1 M in hexanes, 2.0 equiv) was added dropwise and the reaction mixture was stirred at the same temperature for additional 15 min. The reaction mixture was warmed up to room temperature and stirred for 24 h. Then,

saturated aq $NaHCO_3$ solution was added, and the reaction mixture was stirred at room temperature for 1 h. The reaction mixture was extracted with CH_2Cl_2, and the organic layer was dried over anhydrous $MgSO_4$, filtered, and concentrated under reduced pressure. The crude product was purified by flash column chromatography on silica gel to supply the corresponding pyridines.

2-(6-Methyl-4-phenylpyridin-2-yl)propan-2-ol (3a)

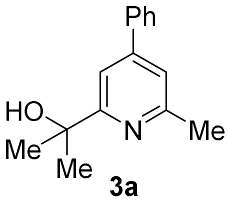

3a

Following the general procedure outlined above, from **2a** (50 mg, 0.153 mmol) and acetone (23 µL, 17.85 mg, 0.307 mmol) in 1.5 mL of CH_2Cl_2, compound **3a** was obtained as a yellow oil (26.7 mg, 0.117 mmol, 76%). R_f = 0.23 (Hexane:EtOAc, 4:1). **^{1}H NMR (400 MHz, $CDCl_3$)** δ 7.65–7.59 (m, 2H), 7.53–7.41 (m, 3H), 7.33 (d, *J* = 1.4 Hz, 1H), 7.26 (s, 1H), 5.57 (bs, 1H), 2.61 (s, 3H), 1.58 (s, 6H). **^{13}C NMR (101 MHz, $CDCl_3$)** δ 165.8, 156.7, 150.0, 138.8, 129.1, 129.0, 127.2, 119.7, 113.8, 71.4, 30.8, 24.3. **HRMS (ESI-TOF) m/z**: Calcd for $C_{15}H_{18}NO$ $[M+H]^+$: 228.1383; found: 228.1384.

2-(6-Butyl-4-phenylpyridin-2-yl)propan-2-ol (3b)

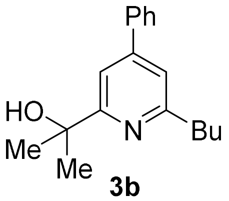

3b

Following the general procedure outlined above, from **2b** (36.8 mg, 0.1 mmol) and acetone (15 µL, 11.62 mg, 0.2 mmol) in 1 mL of CH_2Cl_2, compound **3b** was obtained as a yellow oil (19.9 mg, 0.074 mmol, 74%). R_f = 0.52 (Hexane:EtOAc, 4:1). **^{1}H NMR (400 MHz, $CDCl_3$)** δ 7.66–7.59 (m, 2H), 7.53–7.40 (m, 3H), 7.33 (d, *J* = 1.5 Hz, 1H), 7.25 (d, *J* = 1.4 Hz, 1H), 5.68 (s, 1H), 2.90–2.81 (t,*J* = 7.7 Hz, 2H), 1.84–1.72 (m, 2H), 1.58 (s, 6H), 1.42 (h, *J* = 7.4 Hz, 2H), 0.96 (t, *J* = 7.3 Hz, 3H). **^{13}C NMR (101 MHz, $CDCl_3$)** δ 165.7, 160.7, 150.0, 138.9, 129.0, 128.9, 127.2, 119.1, 113.9, 71.4, 37.8, 31.7, 30.8, 22.5, 14.0. **HRMS (ESI-TOF) m/z**: Calcd for $C_{18}H_{24}NO$ $[M+H]^+$: 270.1852; found: 270.1854.

2-(4,6-Diphenylpyridin-2-yl)propan-2-ol (3c)

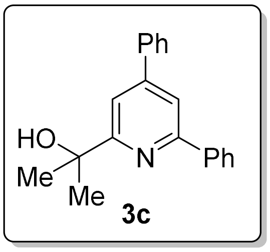

Following the general procedure outlined above, from **2c** (50 mg, 0.129 mmol) and acetone (19 µL, 14.99 mg, 0.258 mmol) in 1.3 mL of CH_2Cl_2, compound **3c** was obtained as a yellow oil (28 mg, 0.097 mmol, 75%). R_f = 0.5 (Hexane:EtOAc, 4:1). **^{1}H NMR (400 MHz, $CDCl_3$)** δ 8.10 (dd, *J* = 6.9, 1.6 Hz, 2H), 7.85 (d, *J* = 1.4 Hz, 1H), 7.70 (dd, *J* = 6.7, 1.6 Hz, 2H), 7.57–7.43 (m, 7H), 1.65 (s, 6H). **^{13}C NMR (101 MHz, $CDCl_3$)** δ 166.4, 155.6, 150.8, 138.9, 138.8, 129.3, 129.2, 129.2, 128.8, 127.3, 127.1, 117.1, 115.4, 71.9, 30.9. **HRMS (ESI-TOF) m/z**: Calcd for $C_{20}H_{20}NO$ $[M+H]^+$: 290.1539; found: 290.1537.

2-(4-Phenylpyridin-2-yl)propan-2-ol (3d)

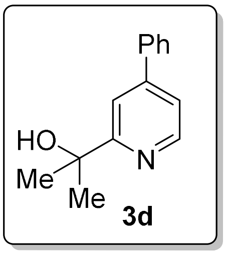

Following the general procedure outlined above, from **2d** (31.1 mg, 0.1 mmol) and acetone (15 µL, 11.62 mg, 0.2 mmol) in 1 mL of CH_2Cl_2, compound **3d** was obtained as an off white solid (9 mg, 0.042 mmol, 42%). R_f = 0.18 (Hexane:EtOAc, 4:1). Mp = 85–87ºC. **^{1}H NMR (400 MHz, $CDCl_3$)** δ 8.56 (d, *J* = 6.1 Hz, 1H), 7.68–7.61 (m, 2H), 7.57 (dd, *J* = 1.7, 0.9 Hz, 1H), 7.54–7.43 (m, 3H), 7.41 (dd, *J* = 5.2, 1.7 Hz, 1H), 1.60 (s, 6H). **^{13}C NMR (101 MHz, $CDCl_3$)** δ 166.7, 149.6, 147.9, 138.5, 129.1 (2C), 127.2, 120.2, 116.7, 71.9, 30.8. **HRMS (ESI-TOF) m/z**: Calcd for $C_{14}H_{15}NO$ $[M+H]^+$: 214.1226; found: 214.1224.

2-(4-(4-Methoxyphenyl)-6-methylpyridin-2-yl)propan-2-ol (3e)

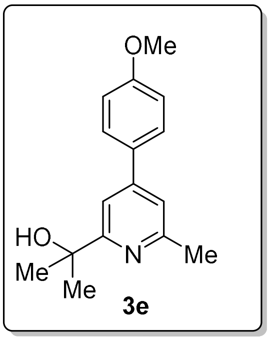

Following the general procedure outlined above, from **2e** (55 mg, 0.154 mmol) and acetone (23 µL, 17.97 mg, 0.31 mmol) in 1.5 mL of CH_2Cl_2, compound **3e** was obtained as a yellow oil (37.5 mg, 0.146 mmol, 94%). $R_f = 0.3$ (Hexane:EtOAc, 4:1). **^{1}H NMR (400 MHz, CDCl$_3$)** δ 7.58 (d, *J* = 8.8 Hz, 2H), 7.29 (d, *J* = 0.7 Hz, 1H), 7.22 (d, *J* = 1.8 Hz, 1H), 7.00 (d, *J* = 8.8 Hz, 2H), 3.86 (s, 3H), 2.59 (s, 3H), 1.57 (s, 6H). **^{13}C NMR (101 MHz, CDCl$_3$)** δ 165.7, 160.5, 156.5, 149.5, 130.9, 128.3, 119.1, 114.5, 113.2, 71.4, 55.4, 30.8, 24.3. **HRMS (ESI-TOF) m/z**: Calcd for $C_{16}H_{20}NO_2$ $[M+H]^+$: 258.1489; found: 258.1489.

2-(4-(2-Bromophenyl)-6-methylpyridin-2-yl)propan-2-ol (3f)

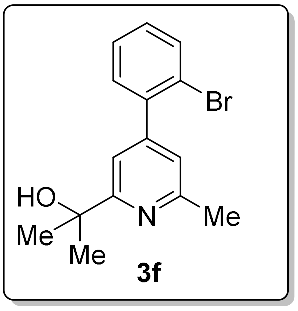

Following the general procedure outlined above, from **2f** (29 mg, 0.074 mmol) and acetone (8 µL, 8.6 mg, 0.148 mmol) in 0.7 mL of CH_2Cl_2, compound **3f** was obtained as a colorless oil (13 mg, 0.044 mmol, 59%). $R_f = 0.55$ (Hexane:EtOAc, 4:1). **^{1}H NMR (400 MHz, CDCl$_3$)** δ 7.69 (dd, *J* = 8.0, 1.2 Hz, 1H), 7.40 (td, *J* = 7.5, 1.2 Hz, 1H), 7.34–7.27 (m, 2H), 7.20 (d, *J* = 1.5 Hz, 1H), 7.09 (d, *J* = 1.4 Hz, 1H), 2.61 (s, 3H), 1.56 (s, 6H). **^{13}C NMR (101 MHz, CDCl$_3$)** δ 165.0, 156.2, 150.0, 140.3, 133.4, 130.8, 129.8, 127.6, 121.9, 121.9, 116.6, 71.4, 30.7, 24.3. **HRMS (ESI-TOF) m/z**: Calcd for $C_{15}H_{17}BrNO$ $[M+H]^+$: 306.0488; found: 306.0489.

2-(3,6-Dimethyl-4-phenylpyridin-2-yl)propan-2-ol (3g)

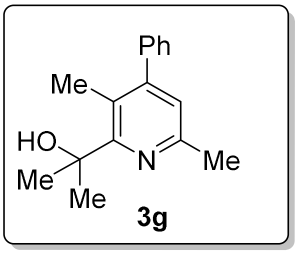

Following the general procedure outlined above, from **2g** (50 mg, 0.147 mmol) and acetone (22 µL, 17.1 mg, 0.295 mmol) in 1.5 mL of CH_2Cl_2, compound **3g** was obtained as a yellow oil (24.5 mg, 0.101 mmol, 69%). $R_f = 0.29$ (Hexane:EtOAc, 4:1). **^{1}H NMR (400 MHz, CDCl$_3$)** δ 7.48–7.37 (m, 3H), 7.27 (d, *J* = 1.6 Hz, 1H), 7.25 (d, *J* = 1.4 Hz, 1H), 6.95 (s, 1H), 2.53 (s, 3H), 2.26 (s, 3H), 1.63 (s, 6H). **^{13}C NMR (101 MHz, CDCl$_3$)** δ 161.9, 153.0, 151.9, 140.2, 128.6, 128.3, 127.7, 123.7, 123.4, 71.3, 29.0, 23.5, 17.2. **HRMS (ESI-TOF) m/z**: Calcd for $C_{16}H_{20}NO$ $[M+H]^+$: 242.1539; found: 242.1538.

2-(4,6-Dimethylpyridin-2-yl)propan-2-ol (3h)

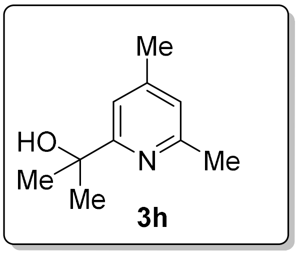

Following the general procedure outlined above, from **2h** (50 mg, 0.19 mmol) and acetone (28 µL, 22 mg, 0.38 mmol) in 1.9 mL of CH_2Cl_2, compound **3h** was obtained as a yellow oil (9.9 mg, 0.06 mmol, 32%). R_f = 0.17 (Hexane:EtOAc, 4:1). **^{1}H NMR (400 MHz, $CDCl_3$)** δ 6.86 (s, 1H), 6.79 (s, 1H), 5.57 (bs, 1H), 2.43 (s, 3H), 2.26 (s, 3H), 1.43 (s, 6H). **^{13}C NMR (101 MHz, $CDCl_3$)** δ 165.0, 155.8, 148.3, 122.3, 116.4, 71.0, 30.8, 24.0, 21.1. **HRMS (ESI-TOF) m/z**: Calcd for $C_{10}H_{16}NO$ $[M+H]^+$: 166.1226; found: 166.1226.

2-(6-Benzyl-4-methylpyridin-2-yl)propan-2-ol (3i)

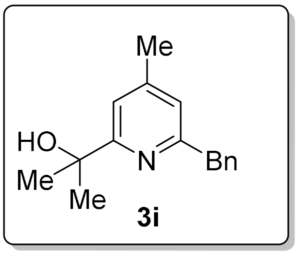

Following the general procedure outlined above, from **2i** (50 mg, 0.147 mmol) and acetone (22 µL, 17.11 mg, 0.295 mmol) in 1.5 mL of CH_2Cl_2, compound **3i** was obtained as a yellow oil (17 mg, 0.07 mmol, 48%). R_f = 0.5 (Hexane:EtOAc, 4:1). **^{1}H NMR (400 MHz, $CDCl_3$)** δ 7.33–7.16 (m, 5H), 6.94 (s, 1H), 6.79 (s, 1H), 5.60 (bs, 1H), 4.09 (s, 2H), 2.28 (s, 3H), 1.49 (s, 6H). **^{13}C NMR (101 MHz, $CDCl_3$)** δ 165.2, 158.4, 148.8, 139.4, 129.1, 128.5, 126.4, 122.0, 117.0, 71.2, 44.3, 30.8, 21.2. **HRMS (ESI-TOF) m/z**: Calcd for $C_{16}H_{20}NO$ $[M+H]^+$: 242.1539; found: 242.1539.

2-(4-(*tert*-Butyl)-6-methylpyridin-2-yl)propan-2-ol (3j)

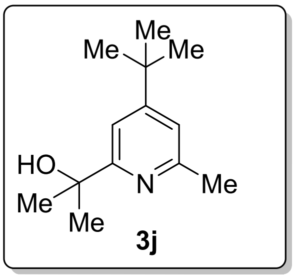

Following the general procedure outlined above, from **2j** (12.7 mg, 0.04 mmol) and acetone (6 µL, 4.83 mg, 0.08 mmol) in 0.4 mL of CH_2Cl_2, compound **3j** was obtained as a yellow oil (5.4 mg, 0.026 mmol, 63%). R_f = 0.4 (Hexane:EtOAc, 4:1). **^{1}H NMR (400 MHz, $CDCl_3$)** δ 7.09 (d, *J* = 1.2 Hz, 1H), 7.03 (d, *J* = 1.6 Hz, 1H), 2.53 (s, 3H), 1.52 (s, 6H), 1.30 (s, 9H). **^{13}C NMR (101 MHz, $CDCl_3$)** δ 165.2, 161.6, 155.9, 118.6, 112.4, 71.4,

34.9, 31.0, 30.8, 24.5. **HRMS (ESI-TOF) m/z**: Calcd for $C_{13}H_{22}NO$ $[M+H]^+$: 208.1696; found: 208.1696.

1-(6-Methyl-4-phenylpyridin-2-yl)cyclohexan-1-ol (3k)

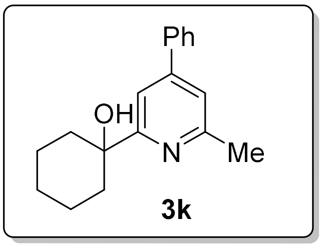

Following the general procedure outlined above, from **2a** (50 mg, 0.153 mmol) and cyclohexanone (32 µL, 30.1 mg, 0.307 mmol) in 1.5 mL of CH_2Cl_2, compound **3k** was obtained as a white solid (29 mg, 0.107 mmol, 70%). R_f = 0.66 (Hexane:EtOAc, 4:1). Mp = 87–90ºC. **^{1}H NMR (400 MHz, $CDCl_3$)** δ 7.62 (dd, J = 8.2, 1.5 Hz, 2H), 7.52–7.41 (m, 3H), 7.34 (d, J = 1.5 Hz, 1H), 7.25 (d, J = 1.5 Hz, 1H), 5.42 (bs, 1H), 2.60 (s, 3H), 1.94–1.64 (m, 10H). **^{13}C NMR (101 MHz, $CDCl_3$)** δ 165.9, 156.6, 149.9, 138.9, 129.0, 128.9, 127.2, 119.6, 114.1, 72.5, 38.9, 25.7, 24.3, 22.2. **HRMS (ESI-TOF) m/z**: Calcd for $C_{18}H_{22}NO$ $[M+H]^+$: 268.1696; found: 268.1694.

(6-Methyl-4-phenylpyridin-2-yl)(phenyl)methanol (3l)

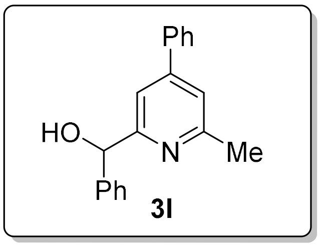

Following the general procedure outlined above, from **2a** (50 mg, 0.153 mmol) and benzaldehyde (31 µL, 32.6 mg, 0.307 mmol) in 1.5 mL of CH_2Cl_2, compound **3l** was obtained as a white solid (38.6 mg, 0.14 mmol, 91%). R_f = 0.18 (Hexane:EtOAc, 4:1). Mp = 122–124ºC. **^{1}H NMR (400 MHz, $CDCl_3$)** δ 7.56–7.49 (m, 2H), 7.47–7.38 (m, 5H), 7.37–7.27 (m, 4H), 7.13–7.08 (m, 1H), 5.76 (s, 1H), 5.64 (bs, 1H), 2.65 (s, 3H). **^{13}C NMR (101 MHz, $CDCl_3$)** δ 160.7, 157.2, 149.9, 143.6, 138.5, 129.1 (2C), 128.7, 127.9, 127.3, 127.2, 120.3, 116.6, 74.9, 24.5. **HRMS (ESI-TOF) m/z**: Calcd for $C_{19}H_{18}NO$ $[M+H]^+$: 276.1383; found: 276.1382. ^{1}H and ^{13}C NMR spectral data are in good agreement with the literature data.[1]

1-(6-Methyl-4-phenylpyridin-2-yl)ethan-1-ol (3m)

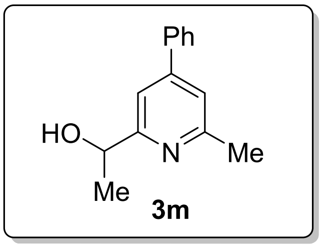

[1] Eidamshaus, C. and Reissig, H.-U. *Eur. J. Org. Chem.*, **2011**: 6056-6069.

Following the general procedure outlined above, from **2a** (50 mg, 0.153 mmol) and acetaldehyde (17 µL, 13.5 mg, 0.307 mmol) in 1.5 mL of CH_2Cl_2, compound **3m** was obtained as a white solid (23.3 mg, 0.109 mmol, 71%). R_f = 0.27 (Hexane:EtOAc, 4:1). Mp =112–114ºC. **^{1}H NMR (400 MHz, CDCl$_3$)** δ 7.62 (dd, *J* = 8.1, 1.5 Hz, 2H), 7.52–7.41 (m, 3H), 7.27 (s, 2H), 4.91 (q, *J* = 6.5 Hz, 1H), 4.58 (bs, 1H), 2.61 (s, 3H), 1.54 (d, *J* = 6.6 Hz, 3H).**^{13}C NMR (101 MHz, CDCl$_3$)** δ 162.8, 157.4, 149.7, 138.6, 129.0, 129.0, 127.1, 119.9, 114.8, 68.7, 24.4, 24.3. **HRMS (ESI-TOF) m/z**: Calcd for $C_{14}H_{16}NO$ $[M+H]^+$: 214.1226; found: 214.1224.

(6-Methyl-4-phenylpyridin-2-yl)diphenylmethanol (3n)

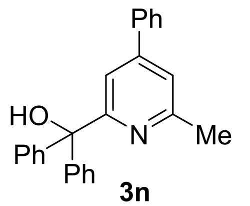

3n

Following the general procedure outlined above, from **2a** (50 mg, 0.153 mmol) and benzophenone (56 mg, 0.307 mmol) in 1.5 mL of CH_2Cl_2, compound **3n** was obtained as a white solid (38.7 mg, 0.11 mmol, 71%). R_f = 0.6 (Hexane:EtOAc, 4:1). Mp = 128–130ºC. **^{1}H NMR (400 MHz, CDCl$_3$)** δ 7.50–7.26 (m, 15H), 7.25 (s, 1H), 7.11 (s, 1H), 6.72 (bs, 1H), 2.62 (s, 3H). **^{13}C NMR (101 MHz, CDCl$_3$)** δ 162.8, 157.0, 149.3, 146.4, 138.4, 129.0, 128.2, 127.9, 127.3, 127.2, 120.2, 118.2, 80.7, 24.3. **HRMS (ESI-TOF) m/z**: Calcd for $C_{25}H_{22}NO$ $[M+H]^+$: 352.1696; found: 352.1693.

1-(6-Methyl-4-phenylpyridin-2-yl)-1-phenylethan-1-ol (3o)

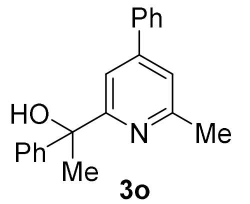

3o

Following the general procedure outlined above, from **2a** (50 mg, 0.153 mmol) and acetophenone (39 µL, 37 mg, 0.307 mmol) in 1.5 mL of CH_2Cl_2, compound **3o** was obtained as a white solid (27.8 mg, 0.096 mmol, 63%). R_f = 0.48 (Hexane:EtOAc, 4:1). Mp = 119–121ºC. **^{1}H NMR (400 MHz, CDCl$_3$)** δ 7.55 (dq, *J* = 6.2, 1.6 Hz, 4H), 7.49–7.39 (m, 3H), 7.32 (dd, *J* = 8.4, 6.8 Hz, 2H), 7.27–7.18 (m, 3H), 2.63 (s, 3H), 1.97 (s, 3H). **^{13}C NMR (101 MHz, CDCl$_3$)** δ 164.5, 156.6, 150.0, 147.4, 138.6, 129.0, 129.0, 128.2, 127.2, 126.9, 126.0, 119.9, 115.5, 74.8, 29.4, 24.3. **HRMS (ESI-TOF) m/z**: Calcd for $C_{20}H_{20}NO$ $[M+H]^+$: 290.1539; found: 290.1539.

General procedure for the synthesis of pyridines 4a and 4b

To a cold (0 °C), stirred solution of the corresponding α-allyl-α-alkyl TosMIC derivative (1.0 equiv) and ethyl acrylate (2.0 equiv) in dry CH_2Cl_2 (0.1 M), under argon atmosphere, a solution of $AlEt_2Cl$ (1 M in hexanes, 2.0 equiv) was added dropwise and the reaction mixture was stirred at the same temperature for additional 15 min. The reaction mixture was warmed up to room temperature and stirred for 24 h. Then saturated aq $NaHCO_3$ solution was added, and the reaction mixture was stirred at room temperature for 1 h. The reaction mixture was extracted with CH_2Cl_2, and the organic layer was dried over anhydrous $MgSO_4$, filtered, and concentrated under reduced pressure. The crude product was purified by flash column chromatography on silica gel to supply the corresponding pyridines.

Ethyl 3-(6-methyl-4-phenylpyridin-2-yl)propanoate (4a)

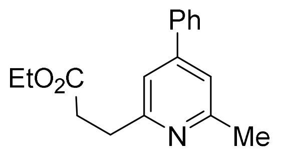

4a

Following the general procedure outlined above, from **2a** (30 mg, 0.092 mmol) in 0.9 mL of CH_2Cl_2, compound **4a** was obtained as a yellow oil (9.3 mg, 0.035 mmol, 37%). R_f = 0.24 (Hexane:EtOAc, 4:1). **^{1}H NMR (400 MHz, $CDCl_3$)** δ 7.64–7.57 (m, 2H), 7.51–7.39 (m, 3H), 7.21 (d, *J* = 1.9 Hz, 2H), 4.14 (q, *J* = 7.1 Hz, 2H), 3.14 (t, *J* = 7.6 Hz, 2H), 2.81 (t, *J* = 7.6 Hz, 2H), 2.58 (s, 3H), 1.23 (t, *J* = 7.2 Hz, 3H). **^{13}C NMR (101 MHz, $CDCl_3$)** δ 173.1, 160.0, 158.4, 138.7, 129.9, 129.0, 128.8, 127.0, 119.0, 118.0, 60.4, 34.0, 33.2, 24.6, 14.3. **HRMS (ESI-TOF) m/z**: Calcd for $C_{17}H_{20}NO_2$ $[M+H]^+$: 270.1489; found: 270.1488.

Ethyl 3-(4-(4-methoxyphenyl)-6-methylpyridin-2-yl)propanoate (4b)

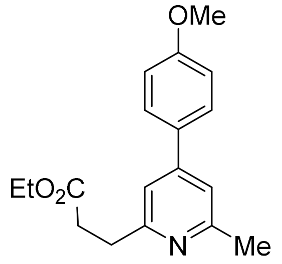

4b

Following the general procedure outlined above, from **2e** (25 mg, 0.07 mmol) in 0.7 mL of CH_2Cl_2, compound **4a** was obtained as a yellow oil (12 mg, 0.04 mmol, 57%). R_f = 0.16 (Hexane:EtOAc, 4:1). **^{1}H NMR (400 MHz, $CDCl_3$)** δ 7.56 (d, *J* = 8.8 Hz, 2H), 7.17 (s, 2H), 6.99 (d, *J* = 8.8 Hz, 2H), 4.13 (q, *J* = 7.1 Hz, 2H), 3.86 (s, 3H), 3.12 (t, *J* = 7.6

Hz, 2H), 2.80 (t, J = 7.6 Hz, 2H), 2.56 (s, 3H), 1.23 (t, J = 7.1 Hz, 3H). **^{13}C NMR (101 MHz, CDCl$_3$)** δ 173.1, 160.0, 159.9, 158.4, 138.7, 129.9, 129.0, 128.8, 127.0, 119.0, 118.0, 60.4, 34.0, 33.2, 24.6, 14.3. **HRMS (ESI-TOF) m/z**: Calcd.for $C_{18}H_{22}NO_3$ $[M+H]^+$: 300.1594, found: 300.1592.

General procedure for the synthesis of pyridines 5a-5d

To a cold (0 °C), stirred solution of the corresponding α-allyl-α-alkyl TosMIC derivative (1.0 equiv) in dry CH_2Cl_2 (0.1 M), under argon atmosphere, $Sc(OTf)_3$ (0.3 equiv) was added and the reaction mixture was stirred at the same temperature for additional 15 min. The reaction mixture was warmed up to room temperature and stirred for 24 h. Then saturated aq $NaHCO_3$ solution was added, and the reaction mixture was stirred at room temperature for 1 h. The reaction mixture was extracted with CH_2Cl_2, and the organic layer was dried over anhydrous $MgSO_4$, filtered, and concentrated under reduced pressure. The crude product was purified by flash column chromatography on silica gel to supply the corresponding disubstituted pyridine.

2-Methyl-4-phenylpyridine (5a)

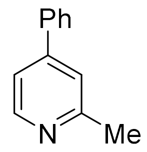

5a

Following the general procedur46 e outlined above, from **2a** (30 mg, 0.092 mmol) in 0.9 mL of CH_2Cl_2, compound **5a** was obtained as a yellow oil (10.9 mg, 0.064 mmol, 70%). R_f = 0.11 (Hexane:EtOAc, 4:1). **^{1}H NMR (400 MHz, CDCl$_3$)** δ 8.54 (d, J = 6.1 Hz, 1H), 7.66–7.59 (m, 2H), 7.52–7.35 (m, 4H), 7.31 (dd, J = 5.3, 2.3 Hz, 1H), 2.63 (s, 3H). **^{13}C NMR (101 MHz, CDCl$_3$)** δ 158.9, 149.6, 148.7, 138.5, 129.0, 128.9, 127.0, 121.2, 118.9, 24.6. **HRMS (ESI-TOF) m/z**: Calcd for $C_{12}H_{12}N$ $[M+H]^+$: 170.0964; found: 170.0964. ^{1}H and ^{13}C NMR spectral data are in good agreement with the literature data.[2]

2,4-Diphenylpyridine (5b)

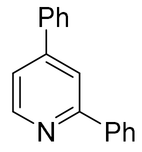

5b

Following the general procedure outlined above, from **2c** (30 mg, 0.077 mmol) in 0.8 mL of CH_2Cl_2, compound **5b** was obtained as a colorless oil (13.9 mg, 0.06 mmol, 78%). R_f = 0.35 (Hexane:EtOAc, 4:1). **^{1}H NMR (400 MHz, CDCl$_3$)** δ 8.75 (d, J = 5.1 Hz, 1H),

[2] Ye W. and Naohiko Y., *J. Am. Chem. Soc.* **2013**, *135*, 3756–3759.

8.09–8.02 (m, 2H), 7.94 (d, *J* = 0.9 Hz, 1H), 7.74–7.67 (m, 2H), 7.56–7.40 (m, 7H). **^{13}C NMR (101 MHz, $CDCl_3$)** δ 158.1, 150.1, 149.3, 139.5, 138.6, 129.2, 129.1, 129.1, 128.8, 127.1, 127.1, 120.3, 118.8. **HRMS (ESI-TOF) m/z**: Calcd for $C_{17}H_{14}N$ $[M+H]^+$: 232.1121; found: 232.1121. ^{1}H and ^{13}C NMR spectral data are in good agreement with the literature data.[2]

4-(4-Methoxyphenyl)-2-methylpyridine (5c)

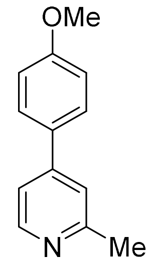

5c

Following the general procedure outlined above, from **2e** (28 mg, 0.078 mmol) in 0.8 mL of CH_2Cl_2, compound **5c** was obtained as a yellow solid (14.1 mg, 0.07 mmol, 90%). R_f = 0.35 (Hexane:EtOAc, 4:1). Mp = 81–83ºC. **^{1}H NMR (400 MHz, $CDCl_3$)** δ 8.50 (dd, *J* = 5.2, 0.8 Hz, 1H), 7.62–7.55 (m, 2H), 7.34 (d, *J* = 1.8 Hz, 1H), 7.27 (dd, *J* = 5.3, 1.4 Hz, 1H), 7.03–6.96 (m, 2H), 3.86 (s, 3H), 2.61 (s, 3H). **^{13}C NMR (101 MHz, $CDCl_3$)** δ 160.61, 158.75, 149.43, 148.52, 130.72, 128.32, 120.82, 118.52, 114.63, 55.54, 24.60. **HRMS (ESI-TOF) m/z**: Calcd for $C_{13}H_{14}NO$ $[M+H]^+$: 200.1070, found: 200.1070.

2-Benzyl-4-methylpyridine (5d)

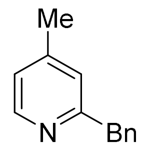

5d

Following the general procedure outlined above, from **2i** (50 mg, 0.147 mmol) in 1.5 mL of CH_2Cl_2, compound **5d** was obtained as a yellow oil after 48h of reaction (15.7 mg, 0.086 mmol, 58%). R_f = 0.2 (Hexane:EtOAc, 4:1). **^{1}H NMR (400 MHz, $CDCl_3$)** δ 8.40 (d, *J* = 6.0 Hz, 1H), 7.35–7.26 (m, 3H), 7.28–7.24 (m, 1H), 7.24–7.17 (m, 1H), 6.93 (d, *J* = 4.3 Hz, 2H), 4.12 (s, 2H), 2.27 (s, 3H). **^{13}C NMR (101 MHz, $CDCl_3$)** δ 160.7, 149.1, 147.6, 139.7, 129.1, 128.6, 126.3, 124.0, 122.3, 44.6, 21.0. **HRMS (ESI-TOF) m/z**: Calcd for $C_{13}H_{14}N$ $[M+H]^+$: 184.1121; found: 184.1121. ^{1}H and ^{13}C NMR spectral data are in good agreement with the literature data.[3]

[3] Rui S., Zhi-Wei Y., Yan W., Song-Li Z. and Lui L., *J. Am. Chem. Soc.* **2010**, *132*, 14391-14393.

General procedure for the synthesis of pyridines 6a-6m

A solution of the corresponding α-allyl-α-alkyl TosMIC derivative (1.0 equiv) and the corresponding *N*-halosuccinimide (2.0 equiv) in dry MeCN (0.1 M) under an argon atmosphere was vigorously stirred at room temperature for 18 h. The mixture was quenched with water (10 mL) and extracted with CH_2Cl_2. The combined organic layers were washed with a saturated solution of $Na_2S_2O_3$, dried over $MgSO_4$ and concentrated under reduced pressure. The crude product was purified by chromatography to give pure 2-iodopyridines.

2-Iodo-6-methyl-4-phenylpyridine (6a)

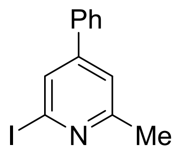

6a

Following the general procedure outlined above, from **2a** (325.4 mg, 1 mmol) and *N*-iodosuccinimide (450 mg, 2 mmol) in 10 mL of MeCN, compound **6a** was obtained as a yellow oil (209.1 mg, 0.708 mmol, 71%). R_f = 0.69 (Hexane:EtOAc, 4:1). **^{1}H NMR (400 MHz, $CDCl_3$)** δ 7.75 (s, 1H), 7.57 (dd, *J* = 7.9, 1.7 Hz, 2H), 7.50–7.43 (m, 3H), 7.31 (s, 1H), 2.58 (s, 3H). **^{13}C NMR (101 MHz, $CDCl_3$)** δ 160.6, 150.5, 136.9, 129.9, 129.4, 129.2, 127.0, 120.8, 118.1, 24.4. **HRMS (ESI-TOF) m/z**: Calcd for $C_{12}H_{11}IN$ $[M+H]^+$: 295.9931; found: 295.9929.

2-Butyl-6-iodo-4-phenylpyridine (6b)

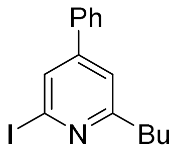

6b

Following the general procedure outlined above, from **2b** (36.8 mg, 0.1 mmol) and *N*-iodosuccinimide (45 mg, 0.2 mmol) in 1 mL of MeCN, compound **6b** was obtained as a yellow oil (21.2 mg, 0.063 mmol, 63%). R_f = 0.8 (Hexane:EtOAc, 4:1). **^{1}H NMR (400 MHz, $CDCl_3$)** δ 7.75 (d, *J* = 1.5 Hz, 1H), 7.61–7.54 (m, 2H), 7.52–7.42 (m, 3H), 7.29 (d, *J* = 1.5 Hz, 1H), 2.84–2.76 (m, 2H), 1.79–1.66 (m, 2H), 1.41 (h, *J* = 7.3 Hz, 2H), 0.95 (t, *J* = 7.4 Hz, 3H). **^{13}C NMR (101 MHz, $CDCl_3$)** δ 165.0, 150.4, 137.0, 130.0, 129.4, 129.1, 127.1, 120.1, 118.4, 38.0, 32.1, 22.5, 14.0. **HRMS (ESI-TOF) m/z**: Calcd for $C_{15}H_{17}IN$ $[M+H]^+$: 338.0400; found: 338.0400.

2-Iodo-4,6-diphenylpyridine (6c)

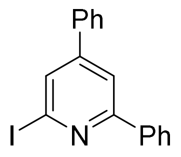

6c

Following the general procedure outlined above, from **2c** (50 mg, 0.129 mmol) and *N*-iodosuccinimide (58.1 mg, 0.258 mmol) in 1.3 mL of MeCN, compound **6c** was obtained as a yellow solid (33.2 mg, 0.093 mmol, 72%). $R_f = 0.78$ (Hexane:EtOAc, 4:1). Mp = 95–97ºC. **^{1}H NMR (400 MHz, $CDCl_3$)** δ 8.03 (dd, *J* = 8.2, 1.6 Hz, 2H), 7.87 (s, 2H), 7.64 (dd, *J* = 7.9, 1.7 Hz, 2H), 7.55–7.42 (m, 6H). **^{13}C NMR (101 MHz, $CDCl_3$)** δ 159.3, 150.9, 137.9, 137.0, 131.1, 129.6, 129.6, 129.2, 128.8, 127.1, 127.1, 119.0, 118.0. **HRMS (ESI-TOF) m/z**: Calcd for $C_{17}H_{13}IN$ $[M+H]^+$: 358.0087; found: 358.0086.

2-Iodo-4-phenylpyridine (6d)

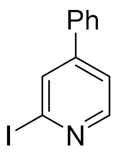

6d

Following the general procedure outlined above, from **2d** (31.1 mg, 0.1 mmol) and *N*-iodosuccinimide (45 mg, 0.2 mmol) in 1 mL of MeCN, compound **6d** was obtained as a yellow oil (8.9 mg, 0.032 mmol, 32%). $R_f = 0.6$ (Hexane:EtOAc, 4:1). **^{1}H NMR (400 MHz, $CDCl_3$)** δ 8.40 (dd, *J* = 5.1, 0.7 Hz, 1H), 7.95 (dd, *J* = 1.7, 0.7 Hz, 1H), 7.59 (dd, *J* = 7.9, 1.7 Hz, 2H), 7.48 (s, 4H). **^{13}C NMR (101 MHz, $CDCl_3$)** δ 150.9, 150.4, 136.6, 132.8, 129.7, 129.3, 127.1, 121.3, 118.9. **HRMS (ESI-TOF) m/z**: Calcd for $C_{11}H_9IN$ $[M+H]^+$: 281.9774; found: 281.9773. ^{1}H and ^{13}C NMR spectral data are in good agreement with the literature data.[4]

2-Iodo-4-(4-methoxyphenyl)-6-methylpyridine (6e)

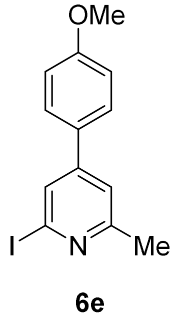

6e

[4] Naofumi H., Nao U. and Yoshiaki N., *Chem. Commun.*, **2021**, *57*, 5957–5960.

Following the general procedure outlined above, from **2e** (50 mg, 0.141 mmol) and *N*-iodosuccinimide (63.3 mg, 0.281 mmol) in 1.4 mL of MeCN, compound **6e** was obtained as a yellow oil (41 mg, 0.126 mmol, 90%). R_f = 0.46 (Hexane:EtOAc, 4:1). **^{1}H NMR (400 MHz, CDCl$_3$)** δ 7.71 (d, *J* = 1.5 Hz, 1H), 7.52 (d, *J* = 8.8 Hz, 2H), 7.26 (d, *J* = 1.5 Hz, 1H), 6.98 (d, *J* = 8.8 Hz, 2H), 3.85 (s, 3H), 2.55 (s, 3H). **^{13}C NMR (101 MHz, CDCl$_3$)** δ 160.8, 160.5, 149.9, 129.3, 129.0, 128.2, 120.2, 118.2, 114.6, 55.4, 24.4. **HRMS (ESI-TOF) m/z**: Calcd for $C_{13}H_{13}INO$ $[M+H]^+$: 326.0036; found: 326.0037.

4-(2-Bromophenyl)-2-iodo-6-methylpyridine (6f)

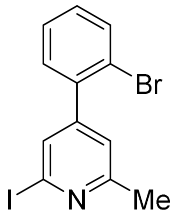

6f

Following the general procedure outlined above, from **2f** (30.4 mg, 0.075 mmol) and *N*-iodosuccinimide (33.8 mg, 0.150 mmol) in 0.8 mL of MeCN, compound **6f** was obtained as a colorless oil (10.3 mg, 0.028 mmol, 37%). R_f = 0.69 (Hexane:EtOAc, 4:1). **^{1}H NMR (400 MHz, CDCl$_3$)** δ 7.67 (dd, *J* = 8.3, 1.3 Hz, 1H), 7.59 (s, 1H), 7.38 (td, *J* = 7.5, 1.2 Hz, 1H), 7.31–7.27 (m, 1H), 7.24 (t, *J* = 1.2 Hz, 1H), 7.16 (s, 1H), 2.58 (s, 3H). **^{13}C NMR (101 MHz, CDCl$_3$)** δ 160.1, 150.4, 138.5, 133.5, 132.2, 130.6, 130.1, 127.7, 123.4, 121.6, 117.0, 24.4. **HRMS (ESI-TOF) m/z**: Calcd for $C_{12}H_{10}BrIN$ $[M+H]^+$: 373.9036; found: 373.9035.

2-Iodo-3,6-dimethyl-4-phenylpyridine (6g)

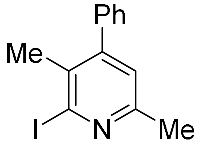

6g

Following the general procedure outlined above, from **2g** (47 mg, 0.138 mmol) and *N*-iodosuccinimide (62.3 mg, 0.277 mmol) in 1.4 mL of MeCN, compound **6g** was obtained as a yellow oil (29 mg, 0.094 mmol, 68%). R_f = 0.85 (Hexane:EtOAc, 4:1). **^{1}H NMR (400 MHz, CDCl$_3$)** δ 7.48–7.37 (m, 3H), 7.24 (dd, *J* = 7.9, 1.7 Hz, 2H), 6.93 (s, 1H), 2.51 (s, 3H), 2.28 (s, 3H). **^{13}C NMR (101 MHz, CDCl$_3$)** δ 157.0, 150.2, 139.6, 132.9, 128.4, 128.4, 128.1, 126.8, 124.0, 24.2, 23.6. **HRMS (ESI-TOF) m/z**: Calcd for $C_{13}H_{13}IN$ $[M+H]^+$: 310.0087; found: 310.0086.

2-Iodo-4,6-dimethylpyridine (6h)

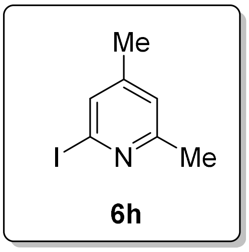

Following the general procedure outlined above, from **2h** (100 mg, 0.38 mmol) and *N*-iodosuccinimide (170.9 mg, 0.759 mmol) in 3.8 mL of MeCN, compound **6h** was obtained as a yellow solid (21.3 mg, 0.091 mmol, 25%). R_f = 0.74 (Hexane:EtOAc, 4:1). Mp = 118–120ºC. **^{1}H NMR (400 MHz, $CDCl_3$)** δ 7.38 (s, 1H), 6.92 (s, 1H), 2.47 (s, 3H), 2.23 (s, 3H). **^{13}C NMR (101 MHz, $CDCl_3$)** δ 158.9, 148.2, 131.6, 122.7, 116.7, 23.1, 19.2. **HRMS (ESI-TOF) m/z**: Calcd for C_7H_9IN $[M+H]^+$: 233.9774; found: 233.9773.

2-Benzyl-6-iodo-4-methylpyridine (6i)

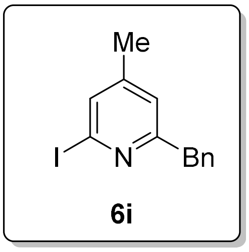

Following the general procedure outlined above, from **2i** (50 mg, 0.147 mmol) and *N*-iodosuccinimide (294.6 mg, 0.759 mmol) in 1.5 mL of MeCN, compound **6i** was obtained as a yellow solid (16.7 mg, 0.054 mmol, 37%). R_f = 0.78 (Hexane:EtOAc, 4:1). Mp = 60-62ºC. **^{1}H NMR (400 MHz, $CDCl_3$)** δ 7.40 (s, 1H), 7.30 (d, J = 6.5 Hz, 3H), 7.24 (s, 2H), 6.80 (s, 1H), 4.08 (s, 2H), 2.18 (s, 3H). **^{13}C NMR (101 MHz, $CDCl_3$)** δ 162.6, 149.5, 138.8, 133.2, 129.2, 128.7, 126.6, 123.4, 117.8, 44.1, 20.4. **HRMS (ESI-TOF) m/z**: Calcd for $C_{13}H_{13}IN$ $[M+H]^+$: 310.0087; found: 310.0087.

4-(*tert*-Butyl)-2-iodo-6-methylpyridine (6j)

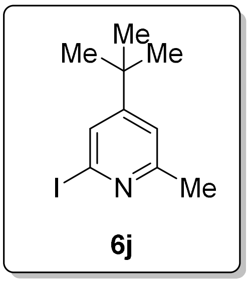

Following the general procedure outlined above, from **2j** (12.5 mg, 0.041 mmol) and *N*-iodosuccinimide (18.4 mg, 0.759 mmol) in 0.4 mL of MeCN, compound **6j** was obtained as a yellow oil (6.4 mg, 0.023 mmol, 57%). R_f = 0.8 (Hexane:EtOAc, 4:1). **^{1}H NMR (400 MHz, $CDCl_3$)** δ 7.52 (d, J = 1.1 Hz, 1H), 7.08 (d, J = 1.2 Hz, 1H), 2.50 (s, 3H), 1.27 (s,

9H). **^{13}C NMR (101 MHz, $CDCl_3$)** δ 162.46, 160.05 (2C), 129.23, 119.99, 34.67, 30.41, 24.38. **HRMS (ESI-TOF) m/z**: Calcd for $C_{10}H_{15}IN$ $[M+H]^+$: 276.0244; found: 276.0244.

2-Iodo-4-methoxy-6-methylpyridine (6k)

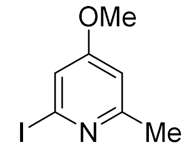

6k

Following the general procedure outlined above, from **2k** (48.8 mg, 0.175 mmol) and *N*-iodosuccinimide (78.5 mg, 0.349 mmol) in 1.8 mL of MeCN, compound **6k** was obtained as a yellow oil (22.1 mg, 0.098 mmol, 56%). R_f = 0.53 (Hexane:EtOAc, 4:1). **^{1}H NMR (400 MHz, $CDCl_3$)** δ 7.08 (d, *J* = 2.1 Hz, 1H), 6.63 (d, *J* = 2.0 Hz, 1H), 3.80 (s, 3H), 2.47 (s, 3H). **^{13}C NMR (101 MHz, $CDCl_3$)** δ 166.0, 161.1, 117.7, 109.6, 55.4, 24.4. **HRMS (ESI-TOF) m/z**: Calcd for C_7H_9INO $[M+H]^+$: 249.9723; found: 249.9721.

2-Bromo-6-methyl-4-phenylpyridine (6l)

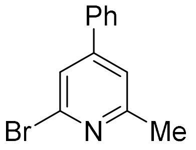

6l

Following the general procedure outlined above, from **2a** (50 mg, 0.154 mmol) and *N*-bromosuccinimide (54.7 mg, 0.307 mmol) in 1.5 mL of MeCN, compound **6l** was obtained as a yellow oil (28 mg, 0.113 mmol, 74%). R_f = 0.55 (Hexane:EtOAc, 4:1). **^{1}H NMR (400 MHz, $CDCl_3$)** δ 7.59 (dd, *J* = 7.9, 1.8 Hz, 2H), 7.54–7.43 (m, 4H), 7.31 (s, 1H), 2.60 (s, 3H). **^{13}C NMR (101 MHz, $CDCl_3$)** δ 160.1, 151.5, 142.0, 137.1, 129.5, 129.2, 127.1, 122.9, 120.4, 24.3. **HRMS (ESI-TOF) m/z**: Calcd for $C_{12}H_{11}BrN$ $[M+H]^+$: 248.0069; found 248.0068.

2-Chloro-6-methyl-4-phenylpyridine (6m)

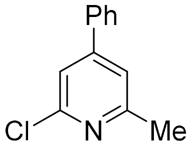

6m

Following the general procedure outlined above, from **2a** (50 mg, 0.154 mmol) and *N*-chlorosuccinimide (41 mg, 0.307 mmol) in 1.5 mL of MeCN, compound **6l** was obtained as a yellow solid (15.6 mg, 0.077 mmol, 50%). R_f = 0.64 (Hexane:EtOAc, 4:1). **^{1}H NMR**

(400 MHz, $CDCl_3$) δ 7.63 – 7.54 (m, 2H), 7.53 – 7.43 (m, 3H), 7.36 (d, *J* = 1.4 Hz, 1H), 7.29 (s, 1H), 2.60 (s, 3H). **^{13}C NMR (101 MHz, $CDCl_3$)** δ 159.6, 151.8, 151.2, 137.2, 129.5, 129.2, 127.1, 120.0, 119.1, 24.3. **HRMS (ESI-TOF) m/z**: Calcd for $C_{12}H_{11}ClN$ $[M+H]^+$: 204.0575; found 204.0575.

Synthesis of 4-methoxy-6-methyl-2,2'-bipyridine (7)

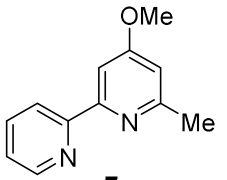

7

2-Bromopyridine (23µL, 0.24 mmol, 1.5 equiv) was added by syringe to a 10mL round bottom flask containing THF (2 mL) under a argon atmosphere. The solution was then cooled to –78°C and t-BuLi (0.29 mL, 1.7 M, 3.1 equiv) was added by syringe. The yellow solution was stirred at –78°C for 20 minutes, then a 2 mL THF solution of anhydrous $ZnCl_2$ (69 mg, 0.5 mmol, 3.2 equiv) was added quickly by cannula and the mixture was allowed to stir at room temperature for 90 minutes. Meanwhile, $Pd_2(dba)_3$ (3.6 mg, 0.025 equiv) and PPh_3 (8.2 mg, 0.2 equiv) were stirred in 2 mL THF for 1 h at room temperature under a argon atmosphere. A 10 mL round bottom flask containing the 2-Iodo-4-methoxy-6-methylpyridine (**6k**) (39 mg, 0.16 mmol, 1 equiv) was fitted with a condenser and purged with argon. The pyridylzinc and palladium catalyst solutions were then added to the solution containing iodopyridine **6k** by cannula, and the mixture was heated to reflux under a argon atmosphere for 16 h. The reaction was quenched by adding EDTA (10 mL, 0.1 M) and the resulting mixture was then diluted with CH_2Cl_2 (10 mL) and made basic with saturated aqueous $NaHCO_3$. The aqueous layer was extracted 3 times with 5 mL portions of CH_2Cl_2, and the combined organics were dried over $MgSO_4$, filtered through a pad of celite, and concentrated under reduced pressure. Purification by flash chromatograhy (Hexane:AcOEt, 7:3) provided **7** (26 mg, 0,13 mmol, 82%) as a pale yellow solid. R_f = 0.15 (Hexane:EtOAc, 4:1). Mp = 60–62 ºC. **^{1}H NMR (400 MHz, $CDCl_3$)** δ 8.65 (dt, *J* = 4.9, 0.9 Hz, 1H), 8.40 (dd, *J* = 9.1, 1.1 Hz, 1H), 7.83–7.74 (m, 2H), 7.32–7.25 (m, 1H), 6.70 (d, *J* = 2.4 Hz, 1H), 3.92 (s, 3H), 2.57 (s, 3H). **^{13}C NMR (101 MHz, $CDCl_3$)** δ 167.00, 159.37, 157.44, 156.35, 149.03, 136.83, 123.64, 121.41, 109.91, 103.53, 55.24, 24.72. **HRMS (ESI-TOF) m/z**: Calcd for $C_{12}H_{13}N_2O$ $[M+H]^+$: 201.1022; found 201.1022. ^{1}H and ^{13}C NMR spectral data are in good agreement with the literature data.[5]

[5] Jyatirmayee D. and Hans-Ulrich R., *Chem. Eur. J.* **2009**, *15*, 6811 – 6814.

Synthesis of 4-methoxy-2-methyl-6-phenylpyridine (8)

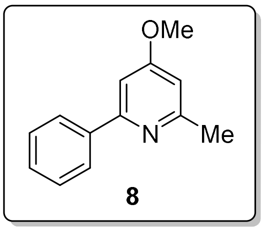

A Biotage microwave vial was charged with iodinated pyridine **6k** (139 mg, 0.56 mmol), phenylboronic acid (102 mg, 0.84 mmol), $Pd(PPh_3)_4$ (32 mg, 0.028 mmol, 5 mol %), and K_2CO_3 (116 mg, 0.84 mmol). Then a mixture of toluene/methanol (4:1, 13 mL) was added, and the vial was placed in a Biotage Initiator system. The reaction mixture was stirred and irradiated with MW at 80 °C for 60 min. The reaction was quenched by addition of water (5 mL) and extracted with CH_2Cl_2. The combined organic layers were dried over anhydrous $MgSO_4$, filtered, and concentrated under reduced pressure. The crude product was purified by flash column chromatography on silica gel (Hexane:EtOAc, 4:1), supplying **8** as a white solid (100 mg, 0.50 mmol, 90%). R_f= 0.64 (Hexane:EtOAc, 4:1). Mp = 70–72 ºC. **^{1}H NMR (400 MHz, $CDCl_3$)** δ 7.98 – 7.91 (m, 2H), 7.49 – 7.43 (m, 2H), 7.43 – 7.35 (m, 1H), 7.04 (d, *J* = 2.2 Hz, 1H), 6.64 (d, *J* = 2.2 Hz, 1H), 3.89 (s, 3H), 2.59 (s, 3H). **^{13}C NMR (101 MHz, $CDCl_3$)** 166.7, 160.0, 158.7, 139.9, 128.8, 128.6, 127.1, 107.2, 104.4, 55.1, 25.0. **HRMS (ESI-TOF) m/z**: Calcd for $C_{13}H_{14}NO$ $[M+H]^+$: 200.1070; found 200.1068.

Synthesis of caerulomycin K (10)

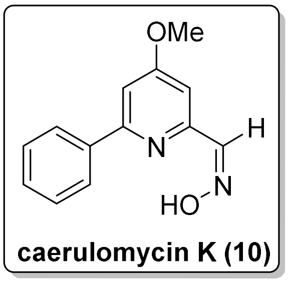

To a solution of **8** (20 mg, 0.1 mmol) in dry 1,4-dioxane (0.5 mL), selenium dioxide (33 mg, 0.3 mmol) was added under an argon atmosphere. The reaction mixture was stirred and reflux at 101 ºC for 48 hours. After TLC showed completed conversion, the reaction mixture was diluted with CH_2Cl_2, washed with saturated solution of $NaHCO_3$ (2x10 mL) and brine (3x10mL). The organic layer was dried over anhydrous $MgSO_4$ and concentrated under reduced pressure. The crude product was purified by flash column chromatography on silica gel (Hexane:EtOAc, 9:1), supplying 4-methoxy-6-phenylpicolinaldehyde as a white solid (14.9 mg, 0.07 mmol, 70%). R_f = 0.48 (Hexane:EtOAc, 4:1). Mp = 102–104 ºC. **^{1}H NMR (400 MHz, $CDCl_3$)** δ 10.13 (s, 1H), 8.08–8.01 (m, 2H), 7.54–7.45 (m, 3H), 7.44 (s, 2H), 3.97 (s, 3H). **^{13}C NMR (101 MHz, $CDCl_3$)** δ 193.9, 167.4, 159.5, 154.7, 138.3, 129.6, 128.9, 127.0, 111.3, 104.7, 55.7.

A mixture of the intermediate 4-methoxy-6-phenylpicolinaldehyde (23 mg, 0.1 mmol), hydroxylamine hydrochloride (38 mg, 0.5 mmol) and pyridine (35 μL, 34 mg, 0.4 mmol) was stirred and refluxed in EtOH (1.5 mL) for 1.5 hours. The solvent was removed under reduced pressure and water (5 mL) was added. Filtration of the precipitate and recrystallization from EtOH:H_2O (1:1) gave caerulomycin K **(10)** (16.4 mg, 0.072, 72%; 50% from pyridine **8**) as a white solid. Mp = 171–173 ºC. **^{1}H NMR (400 MHz, DMSO)** δ 11.68 (s, 1H), 8.15–8.07 (m, 3H), 7.54–7.43 (m, 4H), 7.26 (d, *J* = 2.3 Hz, 1H), 3.94 (s, 3H). **^{13}C NMR (101 MHz, DMSO**) δ 167.0, 158.1, 154.0, 149.5, 138.6, 129.8, 129.1, 127.2, 107.4, 104.3, 56.0. **HRMS (ESI-TOF) m/z**: Calcd for $C_{13}H_{13}N_2O_2$ $[M+H]^+$: 229.0972; found: 229.0972. ^{1}H and ^{13}C NMR spectral data are in good agreement with the literature data.[6]

[6] Peng F., Shuxia W., Kui H., Xia L., Peipei L., Yi W. and Weiming Z., *J. Nat. Prod.* **2011**, *74*, 1751–1756.

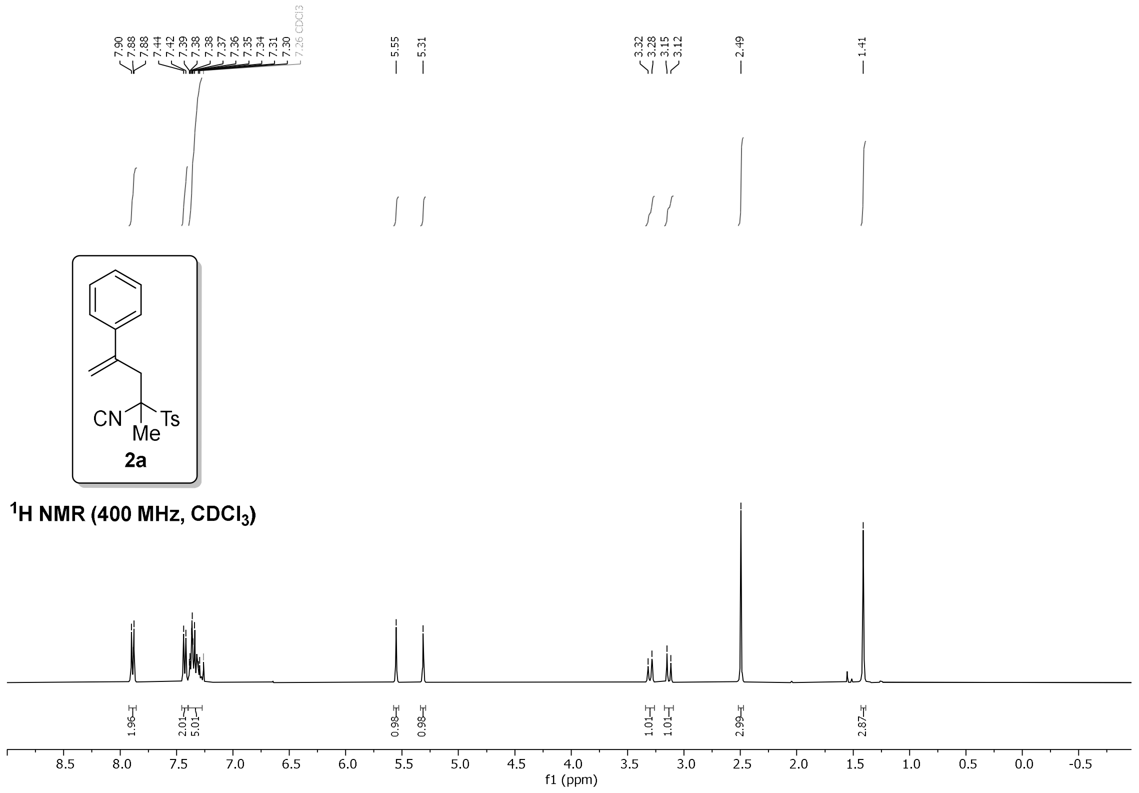
7.90 7.88 7.88 7.44 7.42 7.39 7.38 7.38 7.37 7.36 7.35 7.34 7.31 7.30 7.26 CDCl3
5.55 5.31
3.32 3.28 3.15 3.12
2.49
1.41
CN Ts Me
2a
^{1}H NMR (400 MHz, $CDCl_3$)
1.96 2.01 5.01 0.98 0.98 1.01 1.01 2.99 2.87
8.5 8.0 7.5 7.0 6.5 6.0 5.5 5.0 4.5 4.0 3.5 3.0 2.5 2.0 1.5 1.0 0.5 0.0 -0.5
f1 (ppm)

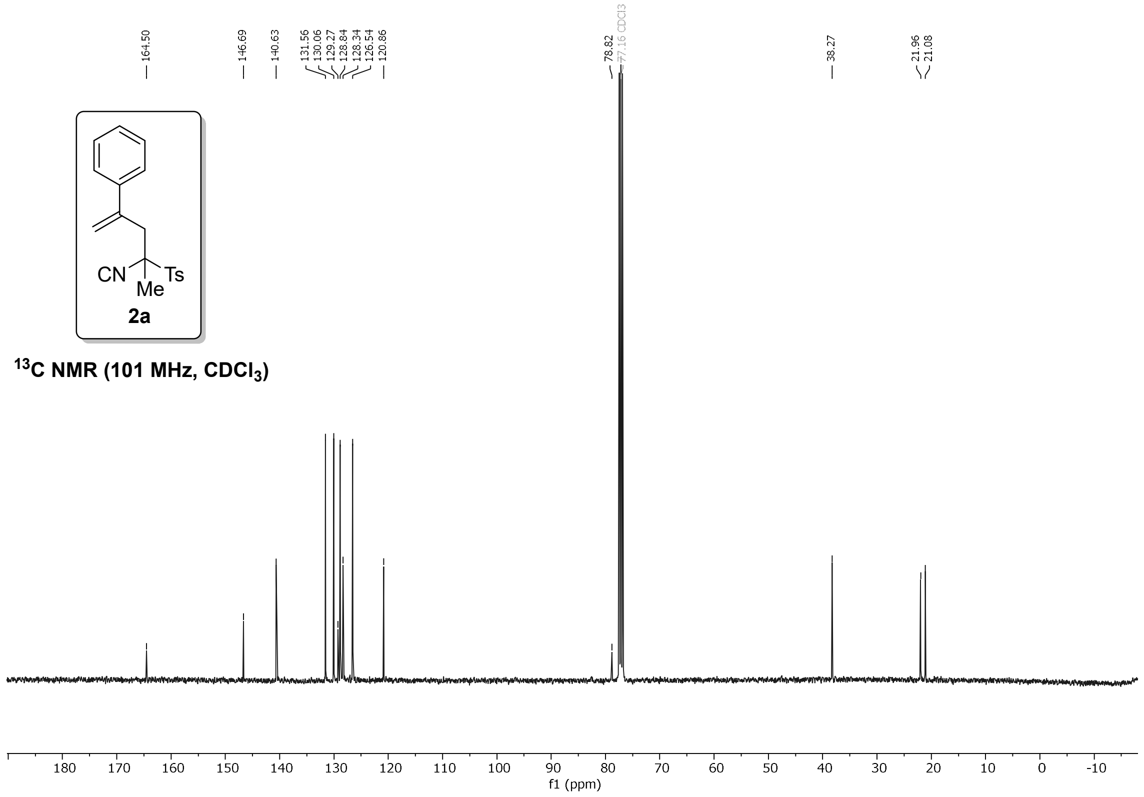
164.50
146.69
140.63
131.56 130.06 129.27 128.84 128.34 126.54 120.86
78.82 77.16 CDCl3
38.27
21.96 21.08
CN Ts Me
2a
^{13}C NMR (101 MHz, $CDCl_3$)
180 170 160 150 140 130 120 110 100 90 80 70 60 50 40 30 20 10 0 -10
f1 (ppm)

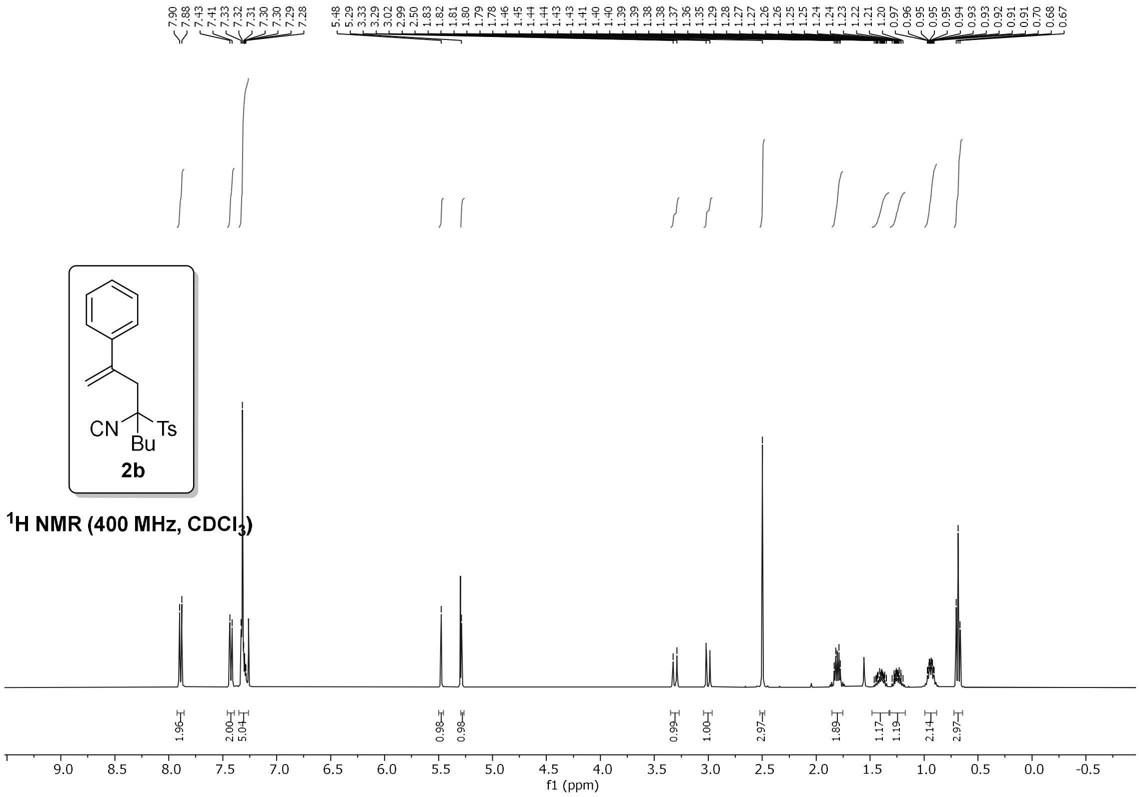

2b

^{1}H NMR (400 MHz, $CDCl_3$)

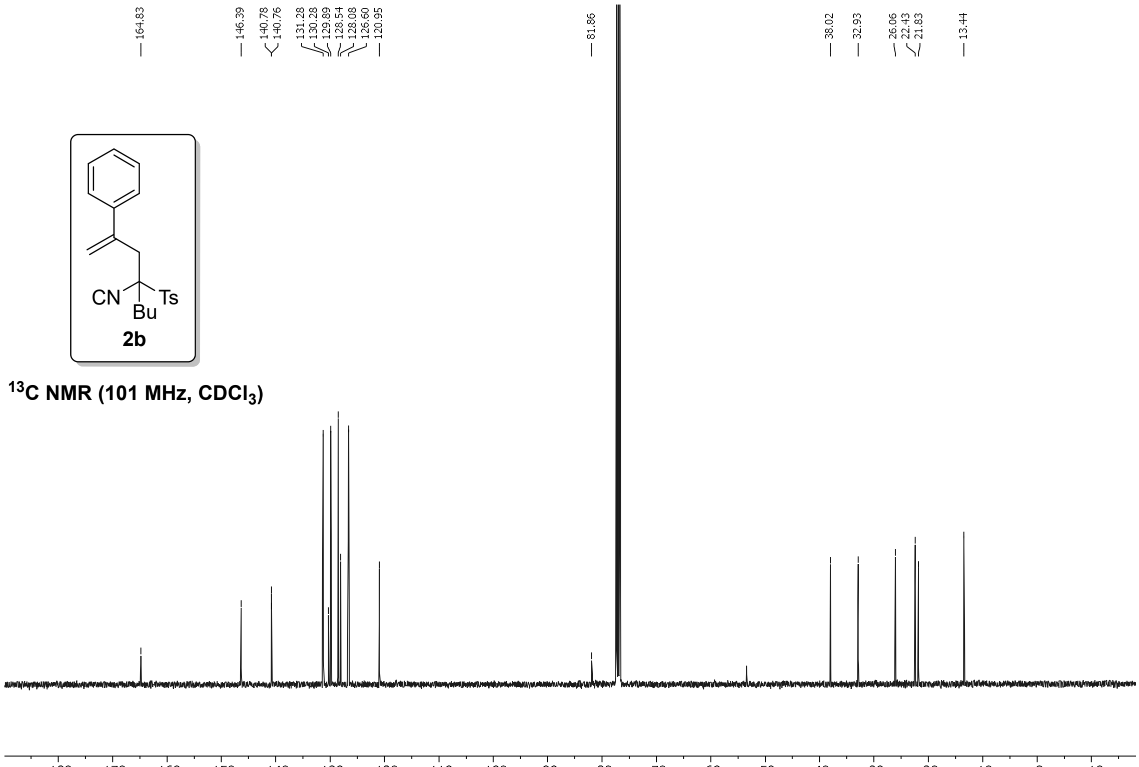

2b

^{13}C NMR (101 MHz, $CDCl_3$)

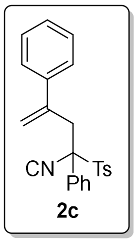

2c

^{1}H NMR (400 MHz, CDCl$_3$)

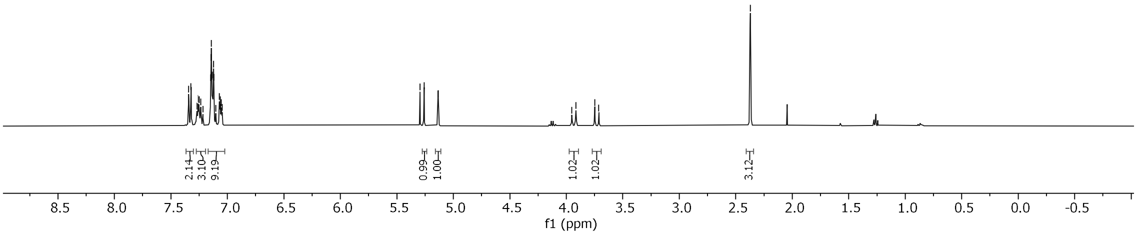

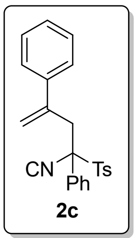

2c

^{13}C NMR (101 MHz, CDCl$_3$)

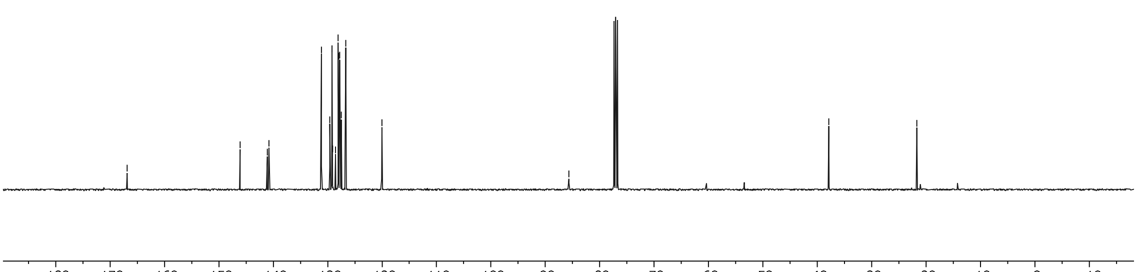

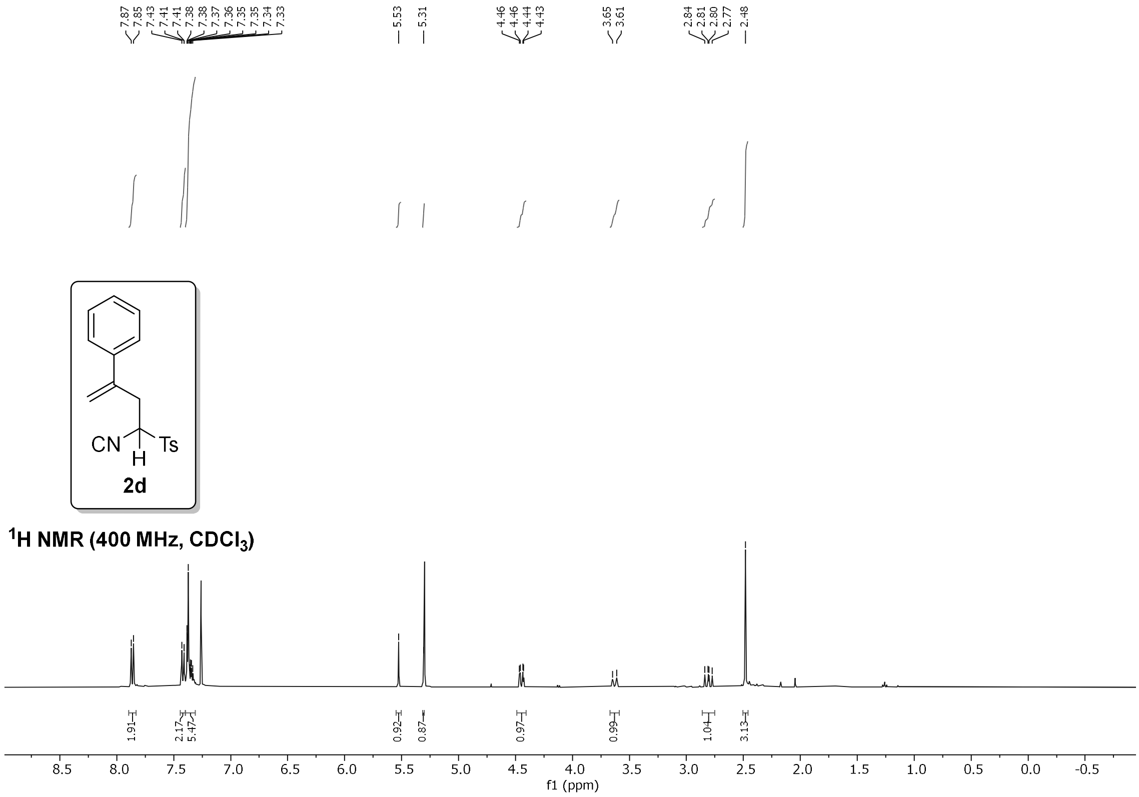

^{1}H NMR (400 MHz, $CDCl_3$)

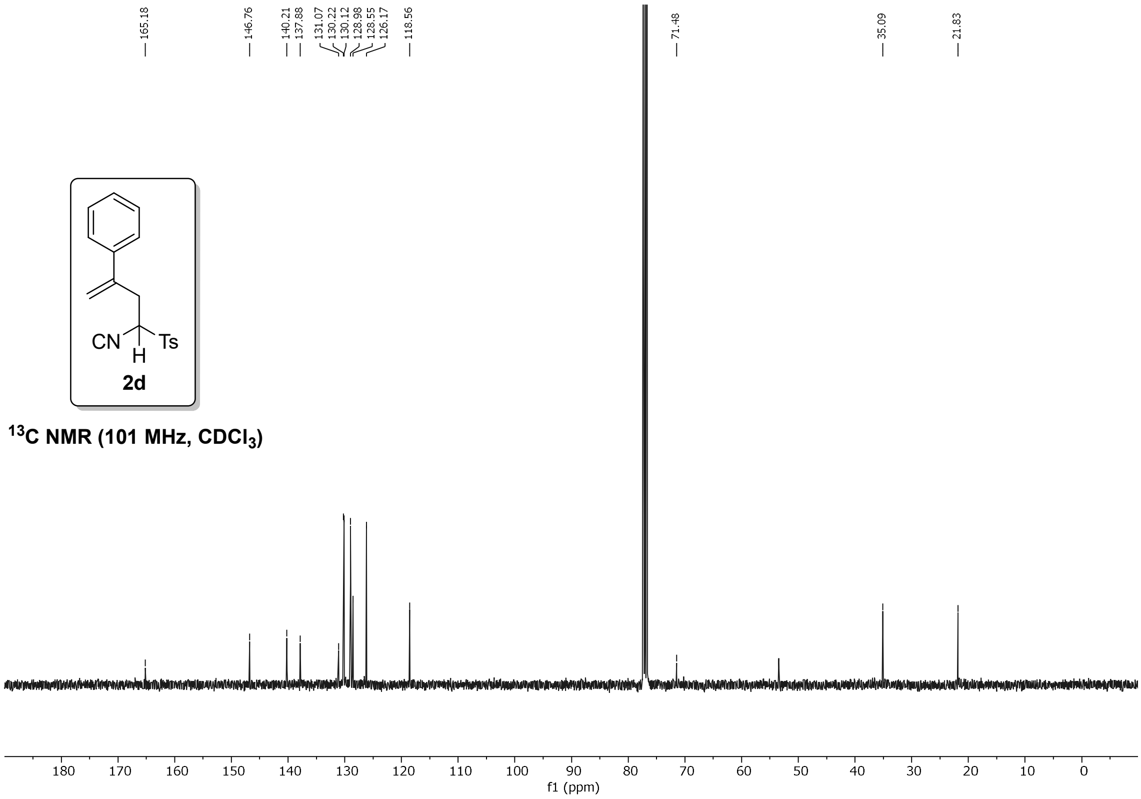

^{13}C NMR (101 MHz, $CDCl_3$)

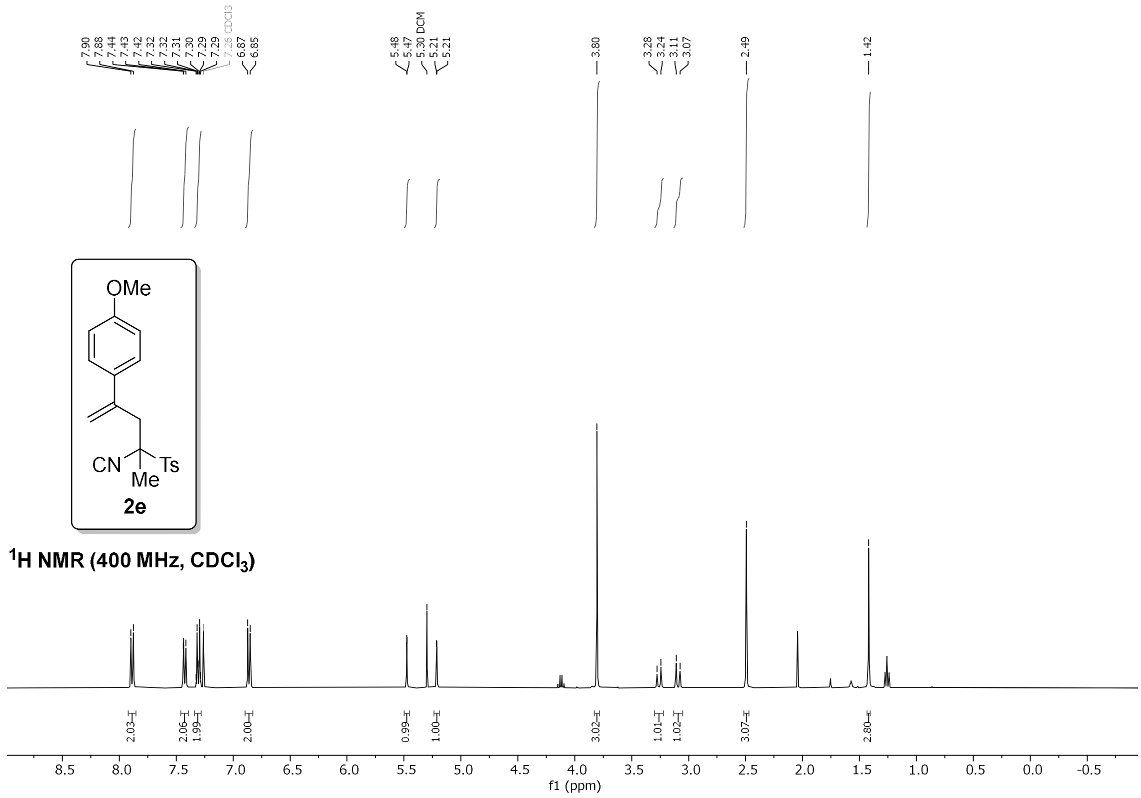
7.90
7.88
7.44
7.43
7.42
7.32
7.32
7.31
7.30
7.29
7.29
7.26 CDCl3
6.87
6.85
5.48
5.47
5.30 DCM
5.21
5.21
3.80
3.28
3.24
3.11
3.07
2.49
1.42
OMe
CN
Ts
Me
2e
1H NMR (400 MHz, CDCl3)
2.03
2.06
1.99
2.00
0.99
1.00
3.02
1.01
1.02
3.07
2.80
f1 (ppm)

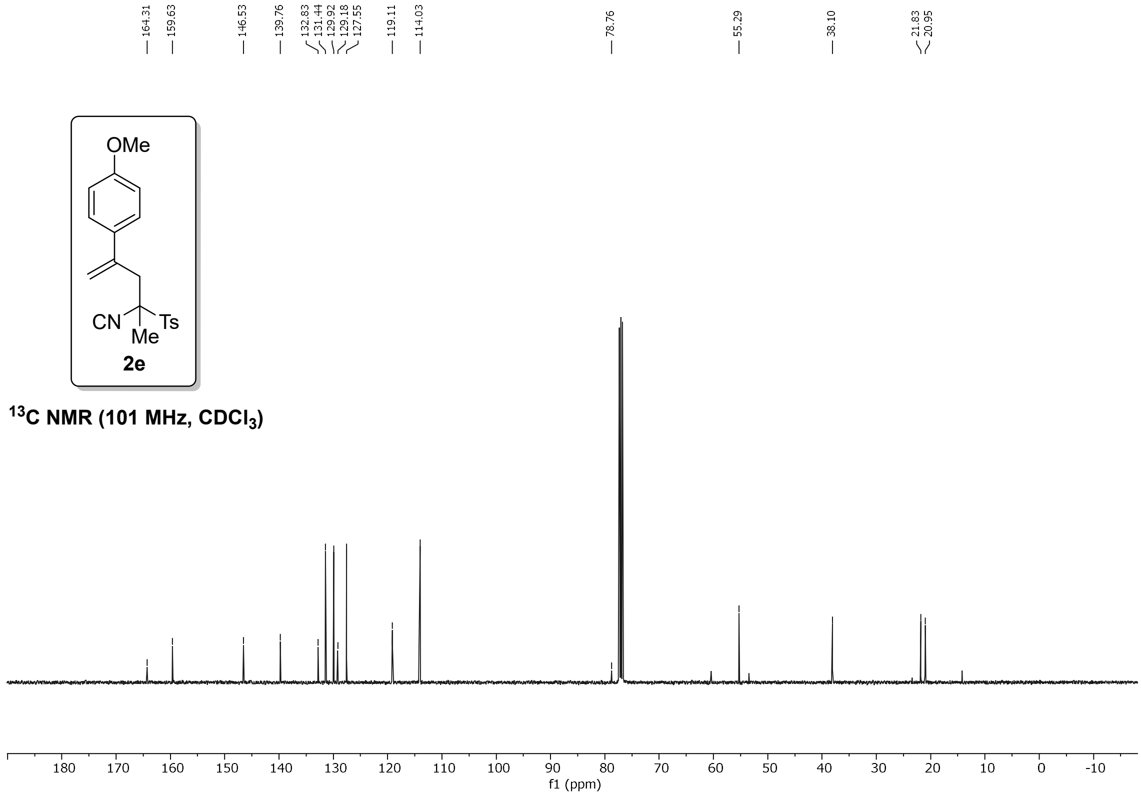
164.31
159.63
146.53
139.76
132.83
131.44
129.92
129.18
127.55
119.11
114.03
78.76
55.29
38.10
21.83
20.95
OMe
CN
Ts
Me
2e
13C NMR (101 MHz, CDCl3)
f1 (ppm)

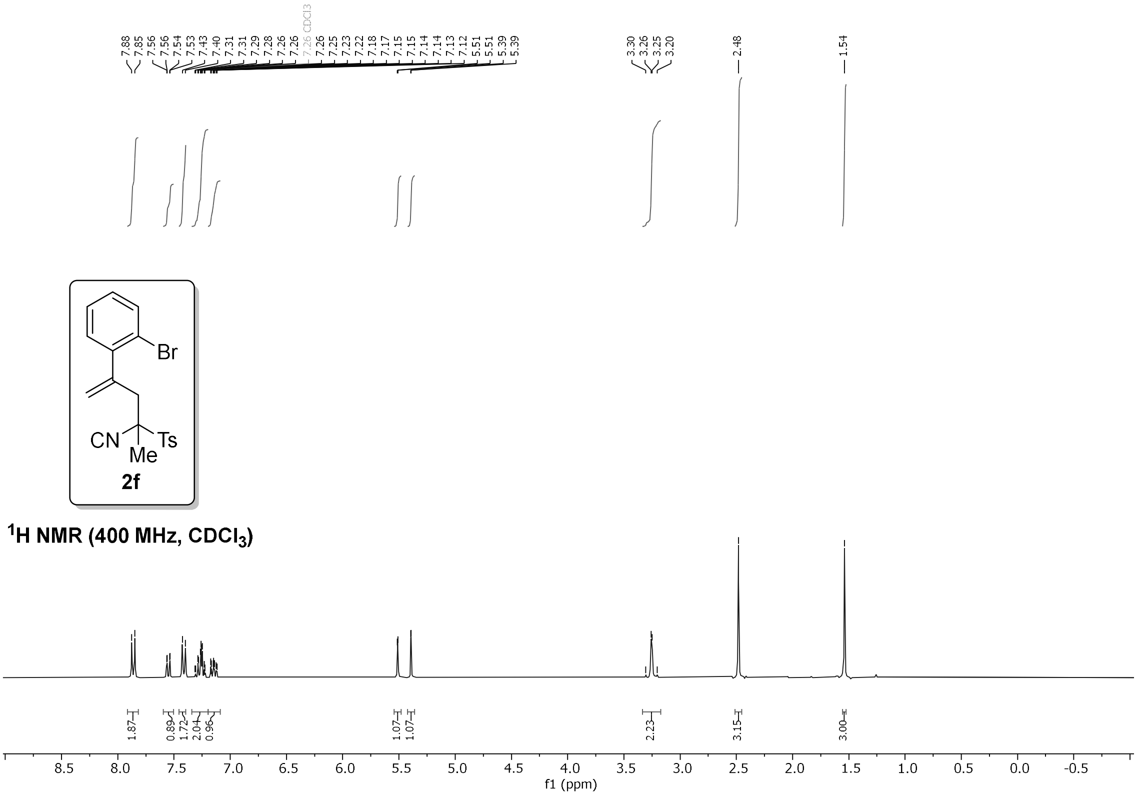

^{1}H NMR (400 MHz, CDCl$_3$)

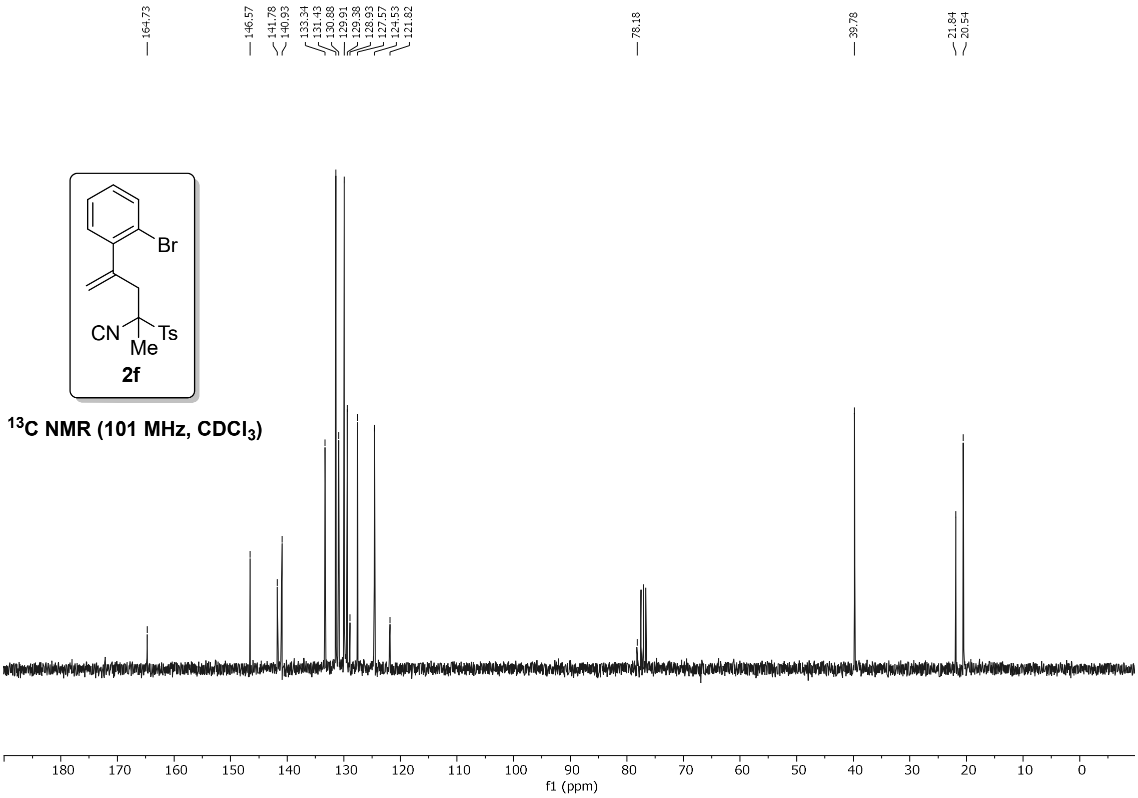

^{13}C NMR (101 MHz, CDCl$_3$)

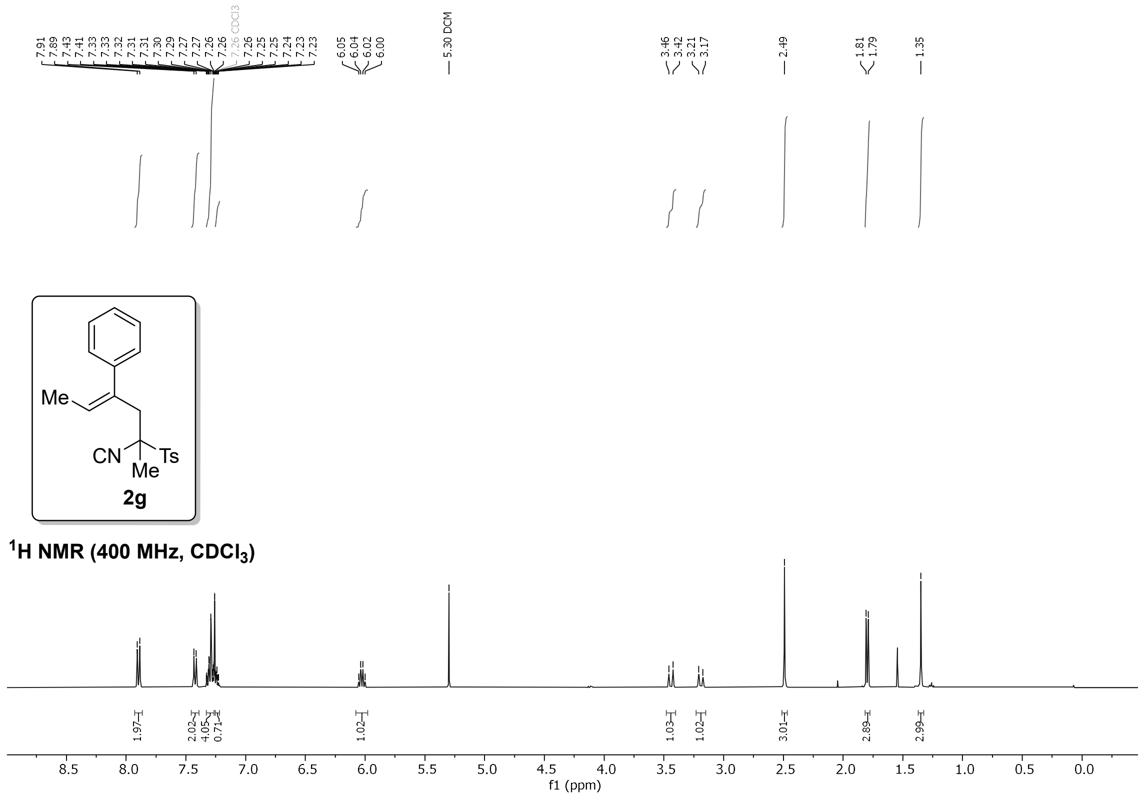

^{1}H NMR (400 MHz, CDCl$_3$)

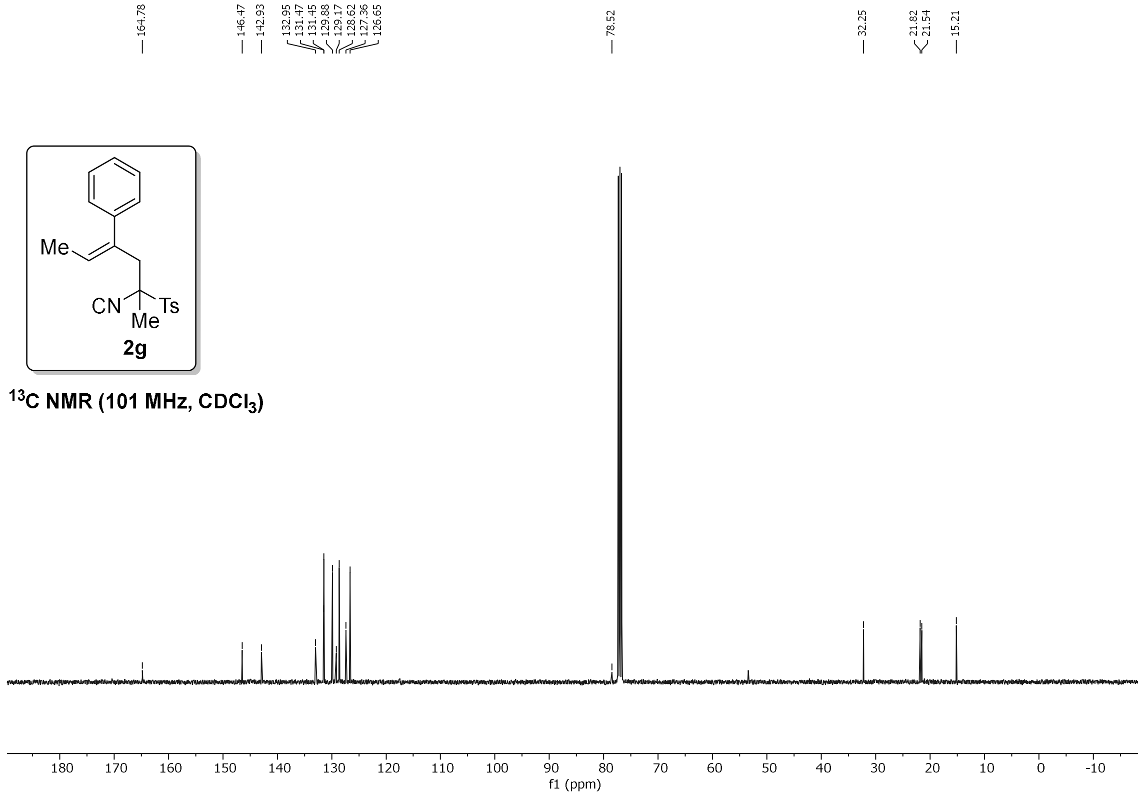

^{13}C NMR (101 MHz, CDCl$_3$)

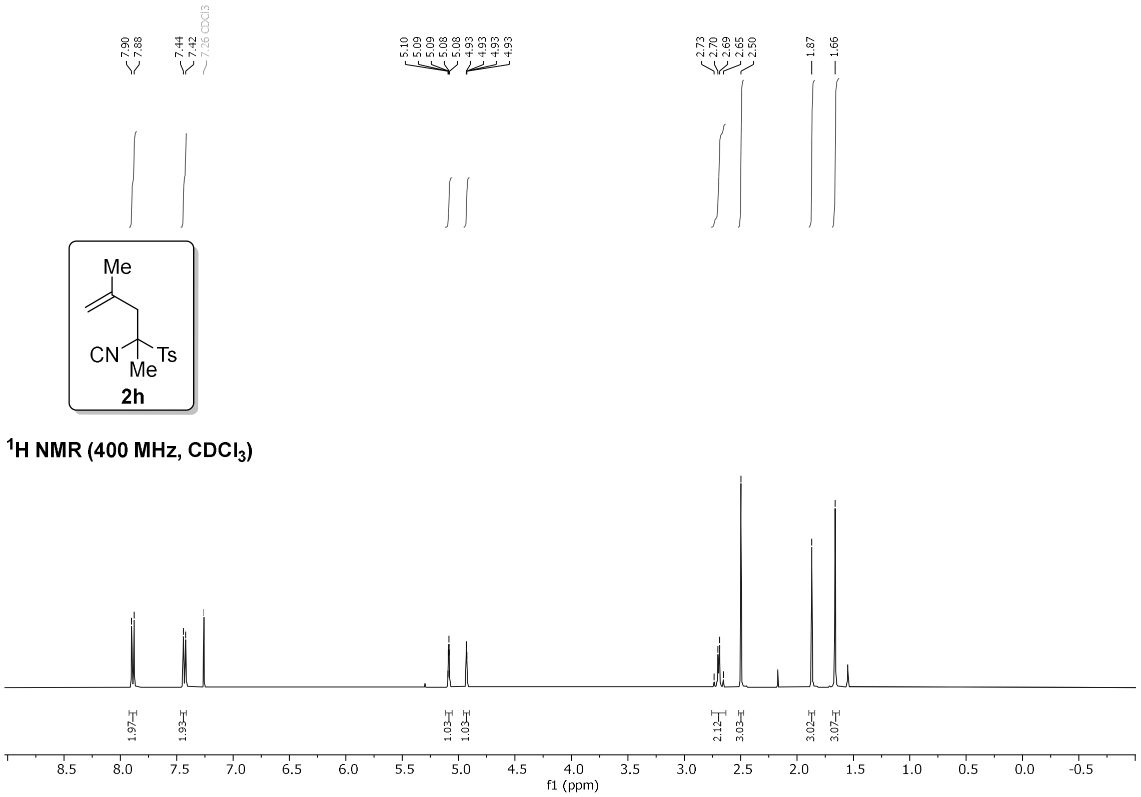
7.90
7.88
7.44
7.42
7.26 CDCl3
5.10
5.09
5.09
5.08
5.08
4.93
4.93
4.93
4.93
2.73
2.70
2.69
2.65
2.50
1.87
1.66
Me
CN
Ts
Me
2h
^{1}H NMR (400 MHz, $CDCl_3$)
1.97
1.93
1.03
1.03
2.12
3.03
3.02
3.07
f1 (ppm)

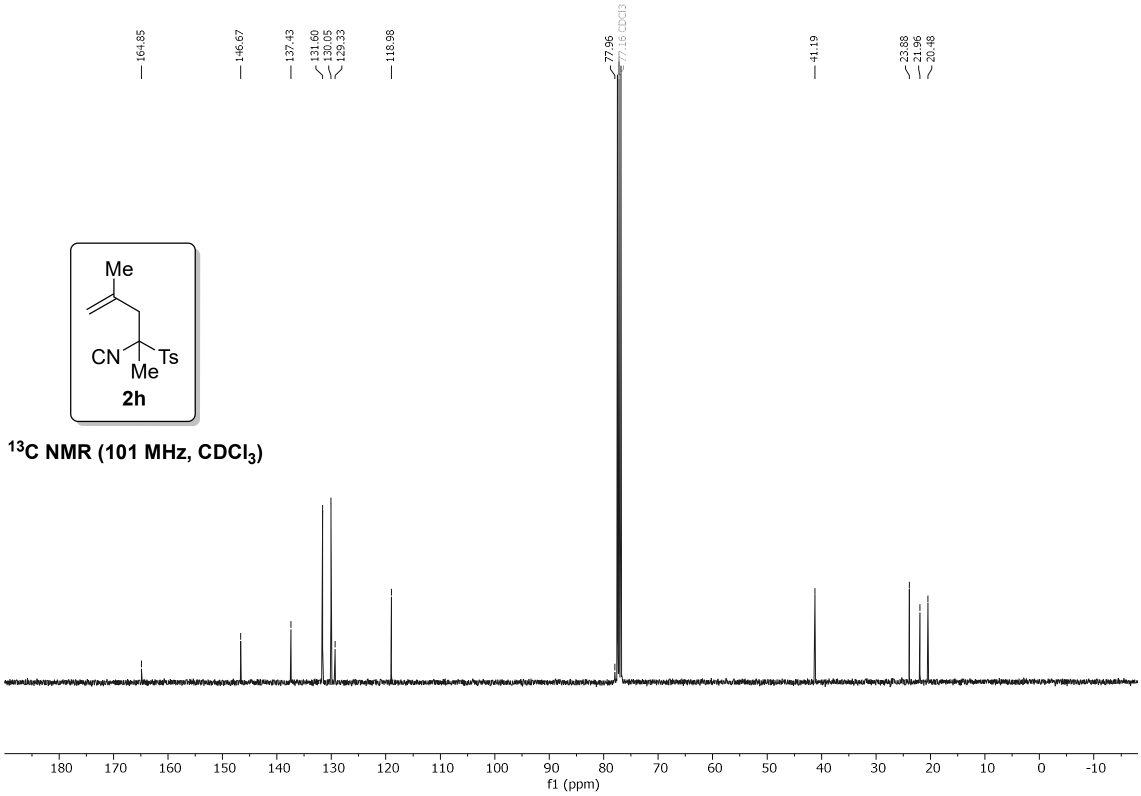
164.85
146.67
137.43
131.60
130.05
129.33
118.98
77.96
77.16 CDCl3
41.19
23.88
21.96
20.48
Me
CN
Ts
Me
2h
^{13}C NMR (101 MHz, $CDCl_3$)
f1 (ppm)

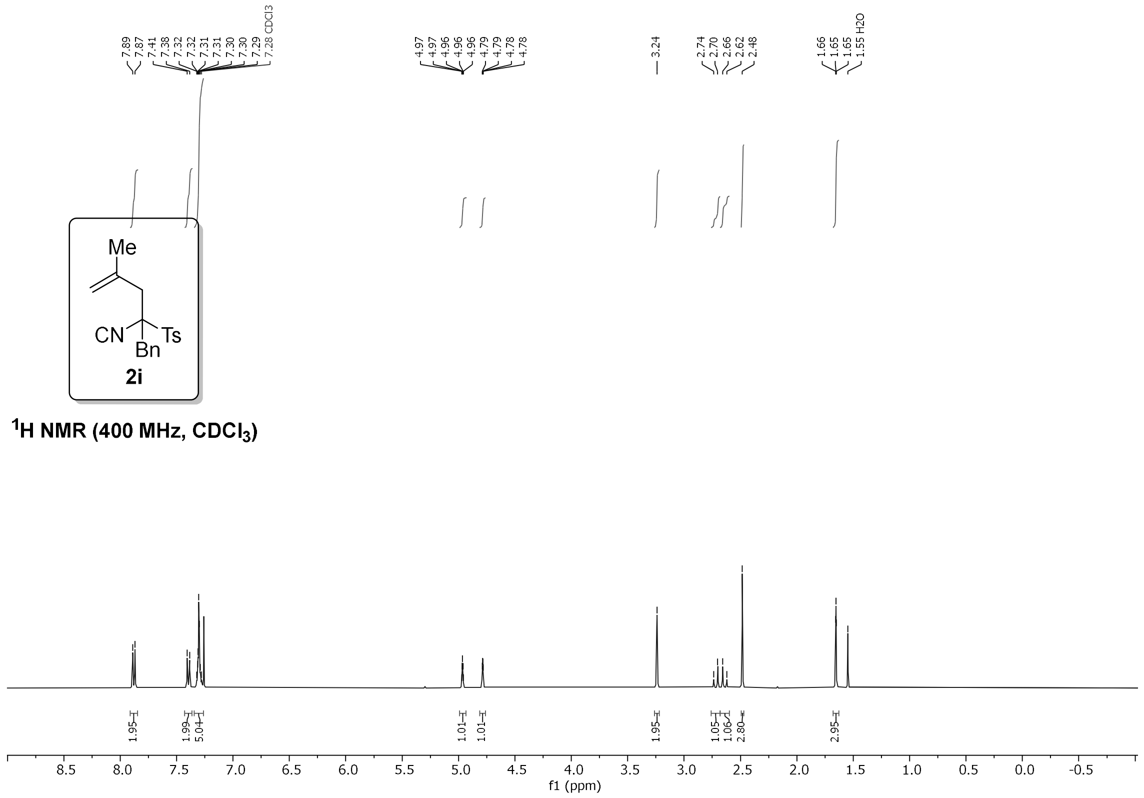

^{1}H NMR (400 MHz, $CDCl_3$)

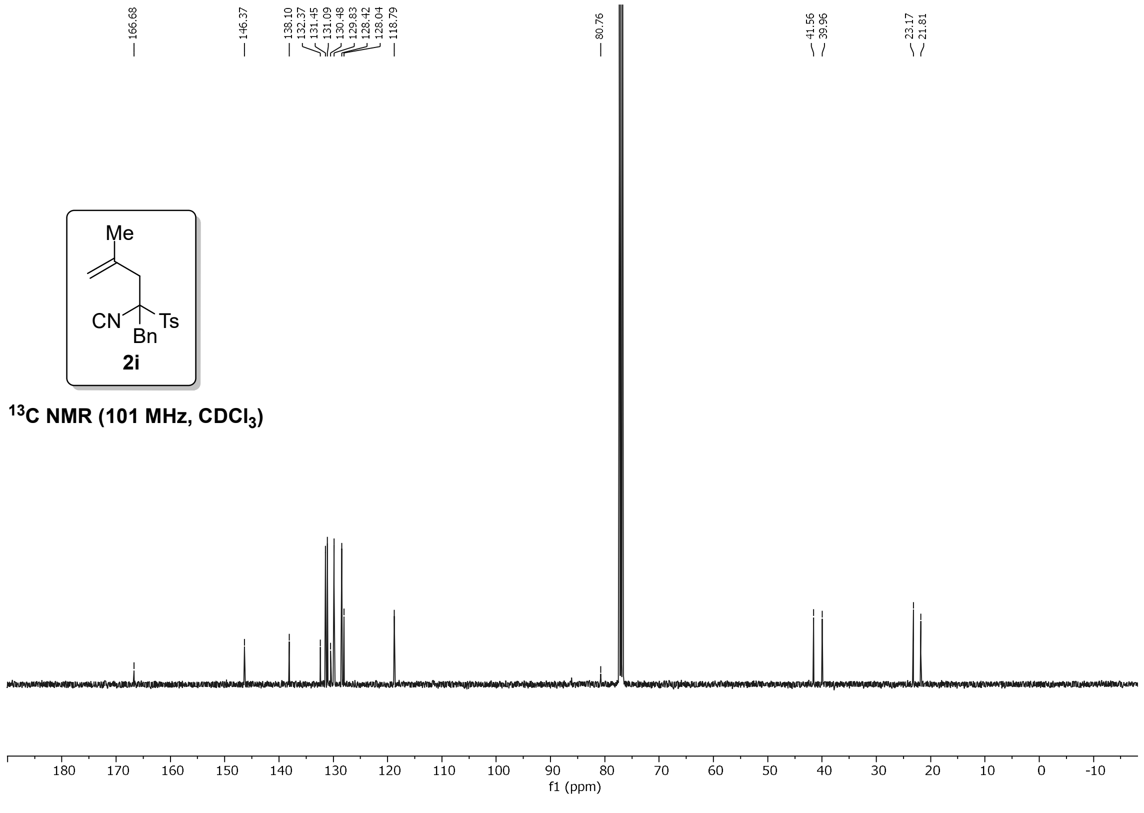

^{13}C NMR (101 MHz, $CDCl_3$)

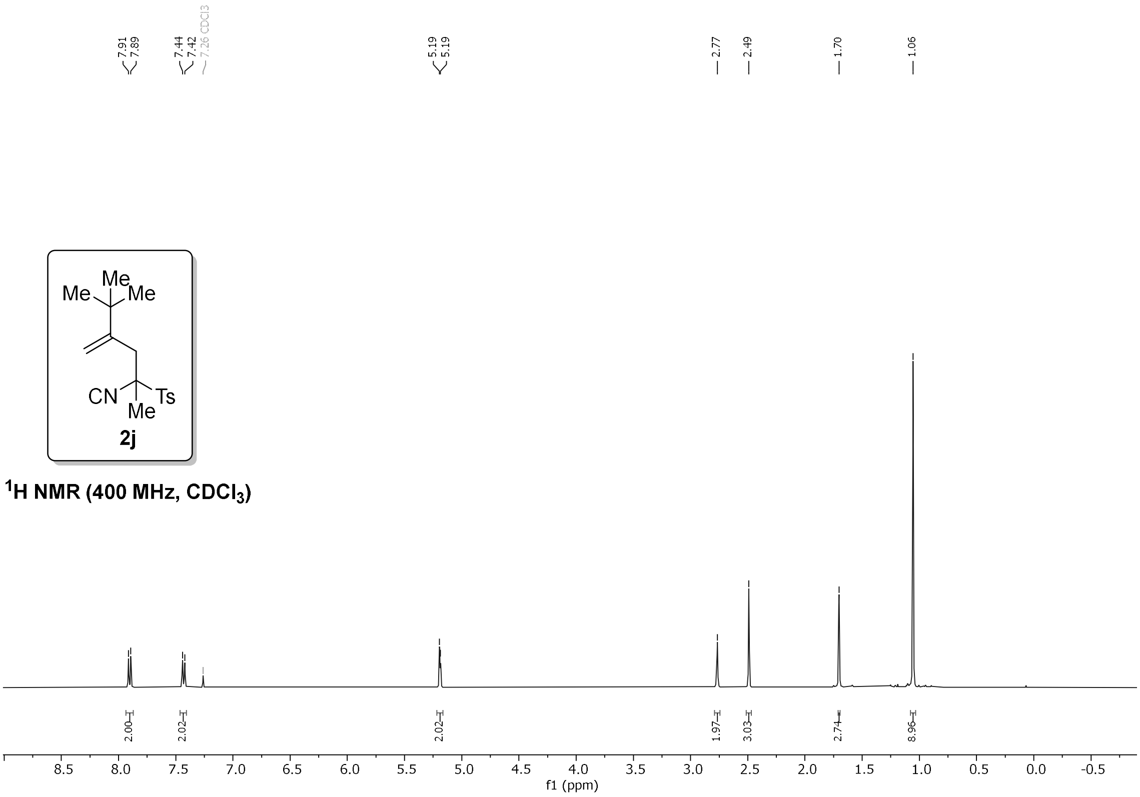

^{1}H NMR (400 MHz, $CDCl_3$)

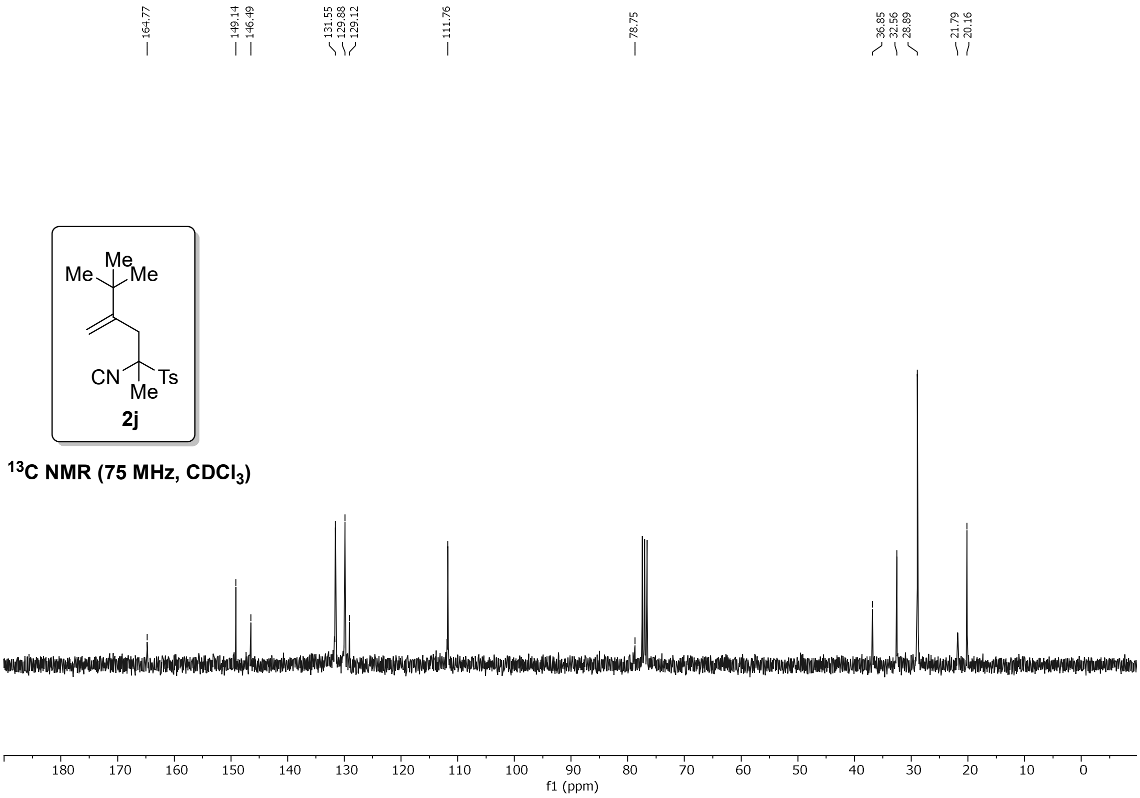

^{13}C NMR (75 MHz, $CDCl_3$)

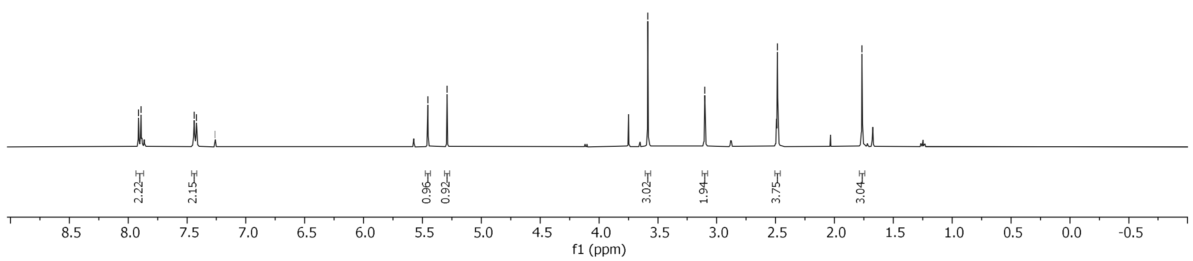

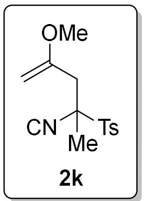

2k

^{1}H NMR (400 MHz, CDCl$_3$)

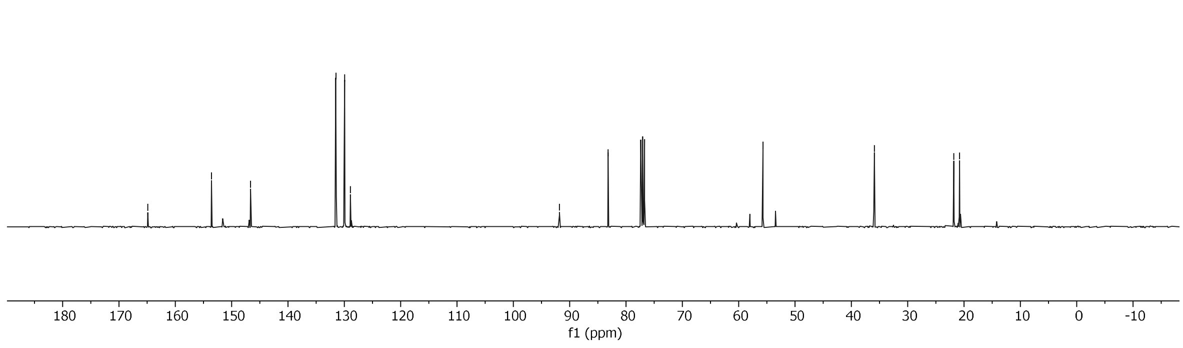

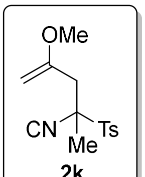

2k

^{13}C NMR (101 MHz, CDCl$_3$)

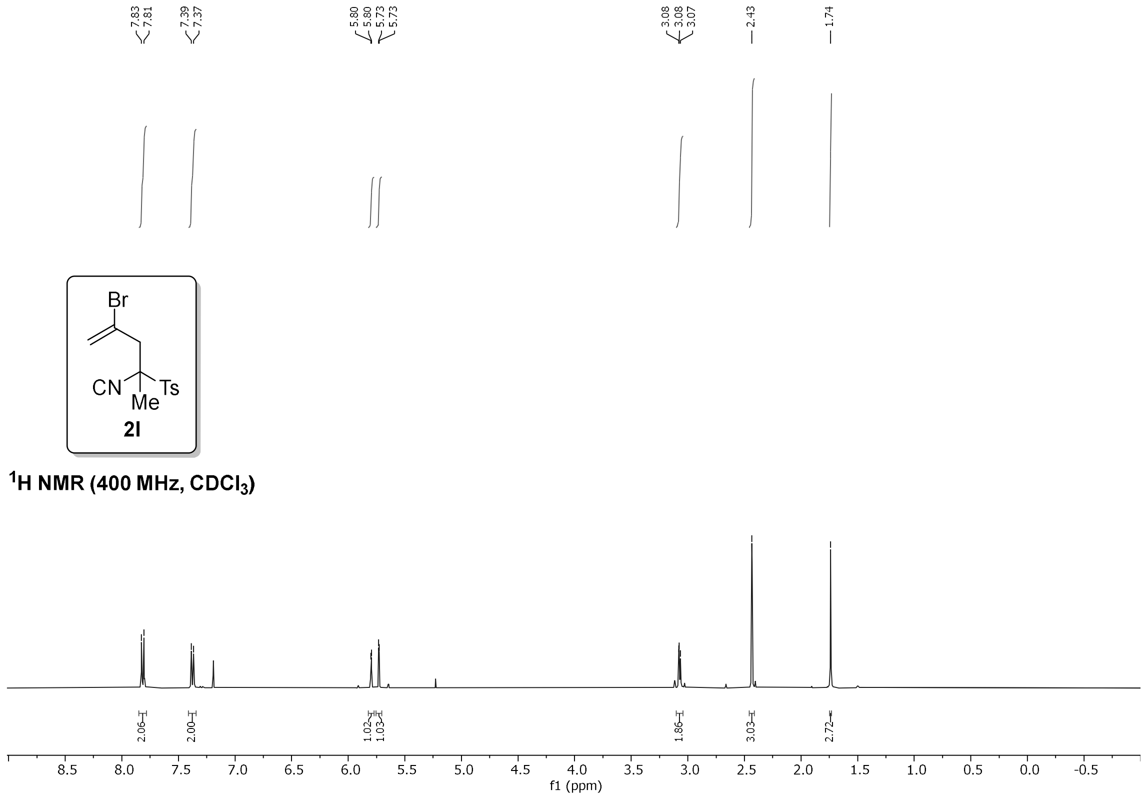
7.83
7.81
7.39
7.37
5.80
5.80
5.73
5.73
3.08
3.08
3.07
2.43
1.74
Br
CN
Ts
Me
2l
^{1}H NMR (400 MHz, CDCl$_3$)
2.06
2.00
1.02
1.03
1.86
3.03
2.72
f1 (ppm)

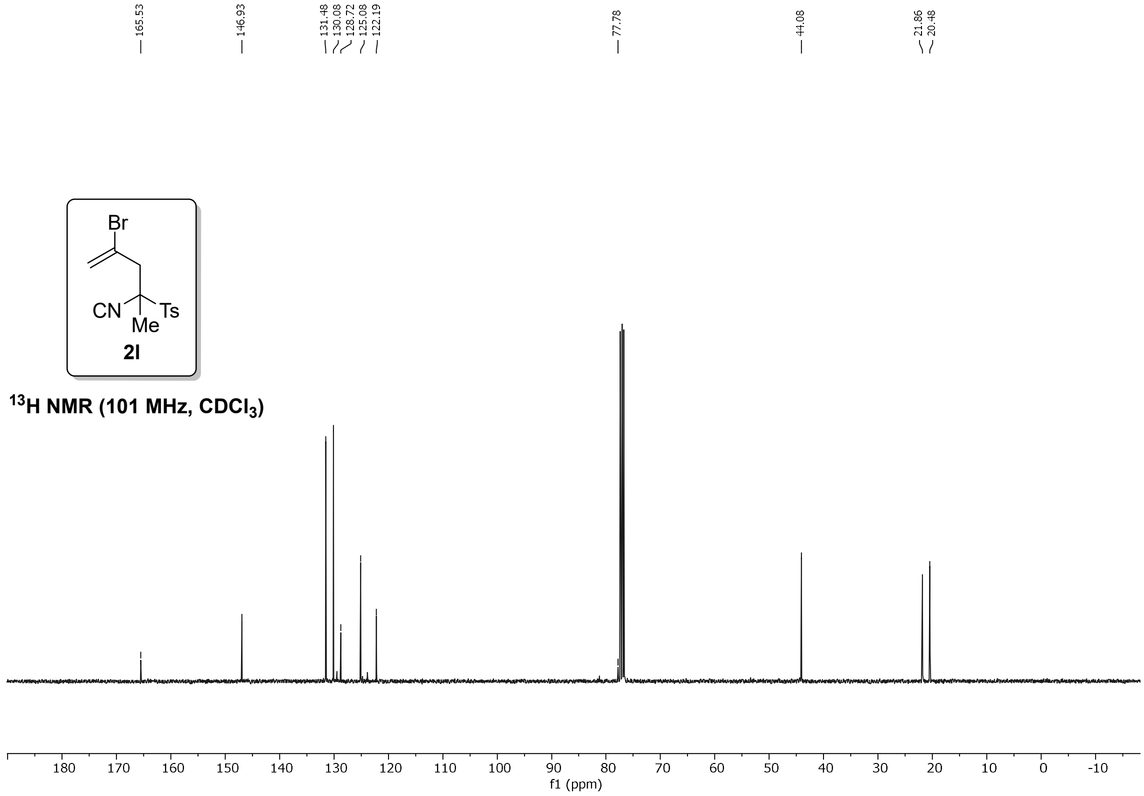
165.53
146.93
131.48
130.08
128.72
125.08
122.19
77.78
44.08
21.86
20.48
Br
CN
Ts
Me
2l
^{13}H NMR (101 MHz, CDCl$_3$)
f1 (ppm)

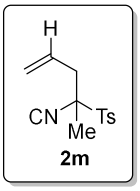

^{1}H NMR (400 MHz, CDCl$_3$)

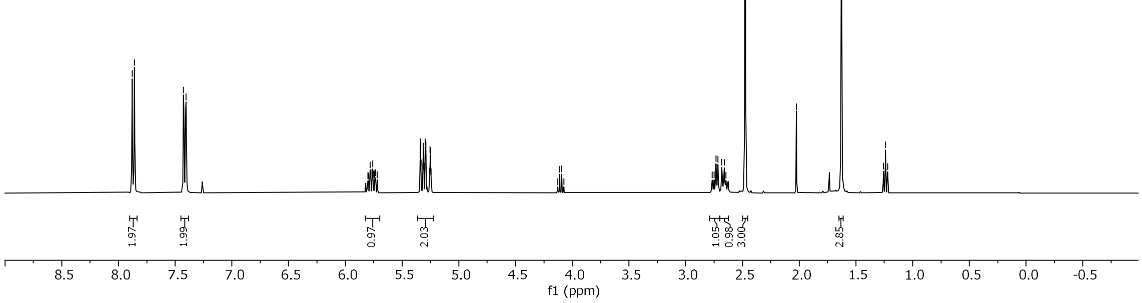

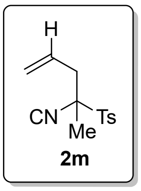

^{13}C NMR (101 MHz, CDCl$_3$)

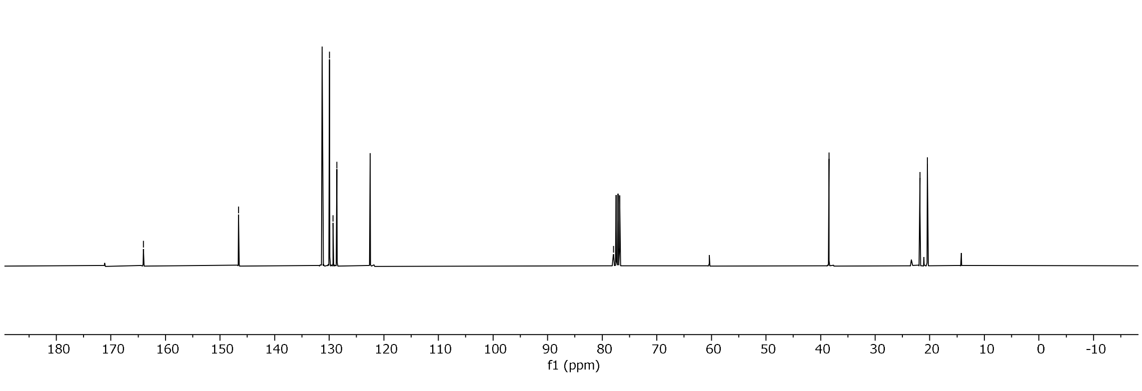

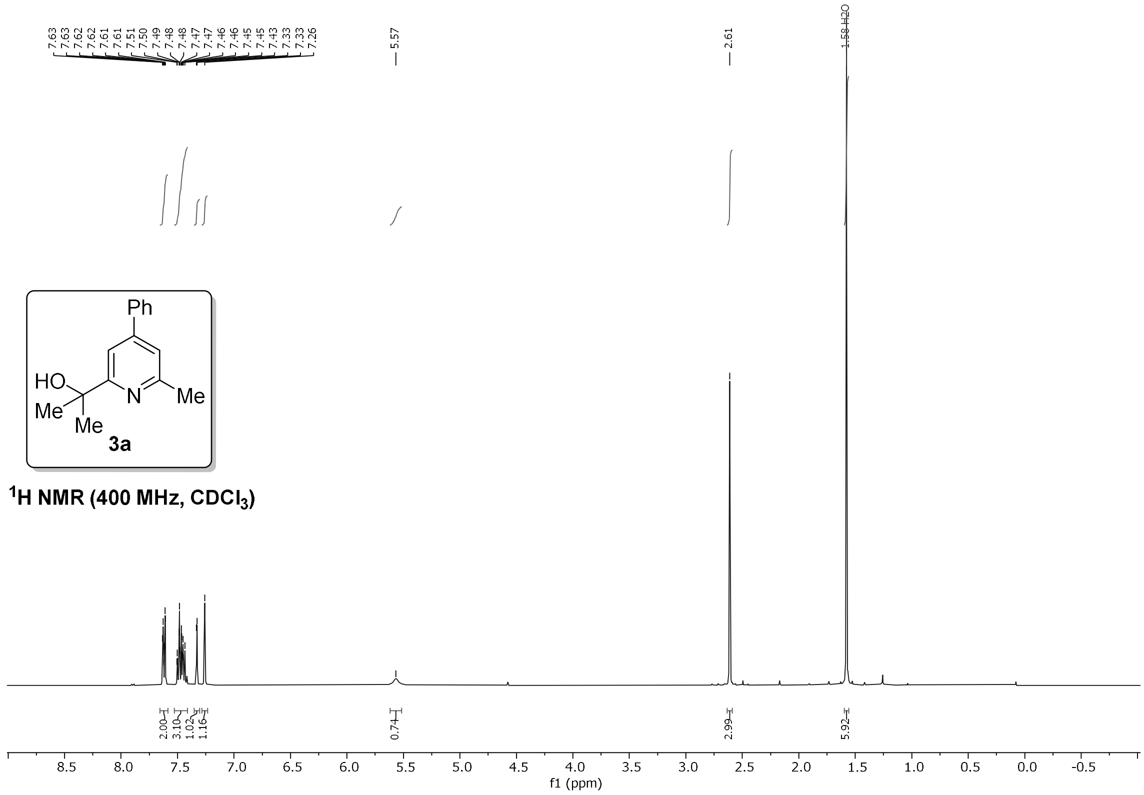

7.63 7.63 7.62 7.62 7.61 7.61 7.51 7.50 7.49 7.48 7.48 7.47 7.47 7.46 7.46 7.45 7.45 7.43 7.33 7.33 7.26
5.57
2.61
1.58 H2O
Ph
HO
N
Me
Me
Me
3a
1H NMR (400 MHz, CDCl3)
2.00 3.10 1.02 1.16
0.74
2.99
5.92
8.5 8.0 7.5 7.0 6.5 6.0 5.5 5.0 4.5 4.0 3.5 3.0 2.5 2.0 1.5 1.0 0.5 0.0 -0.5
f1 (ppm)

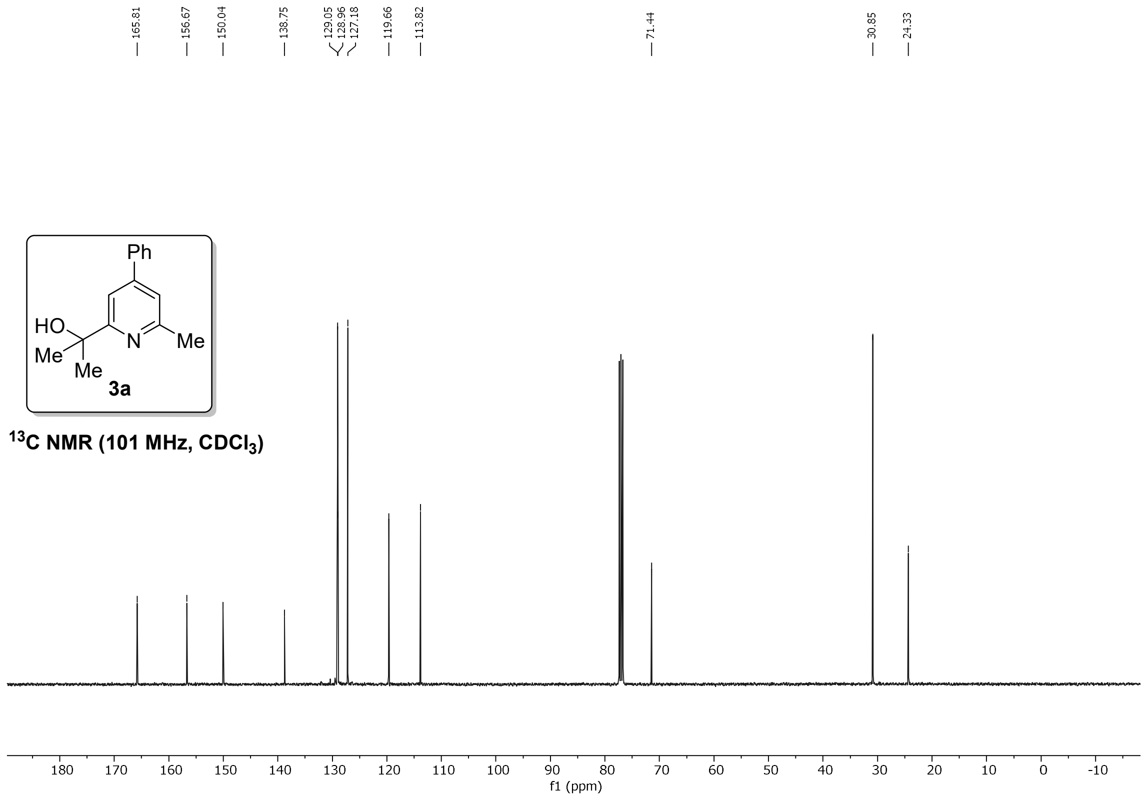

165.81
156.67
150.04
138.75
129.05 128.96 127.18
119.66
113.82
71.44
30.85
24.33
Ph
HO
N
Me
Me
Me
3a
13C NMR (101 MHz, CDCl3)
180 170 160 150 140 130 120 110 100 90 80 70 60 50 40 30 20 10 0 -10
f1 (ppm)

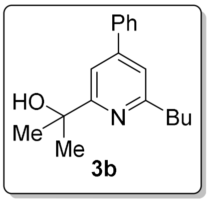

^{1}H NMR (400 MHz, $CDCl_3$)

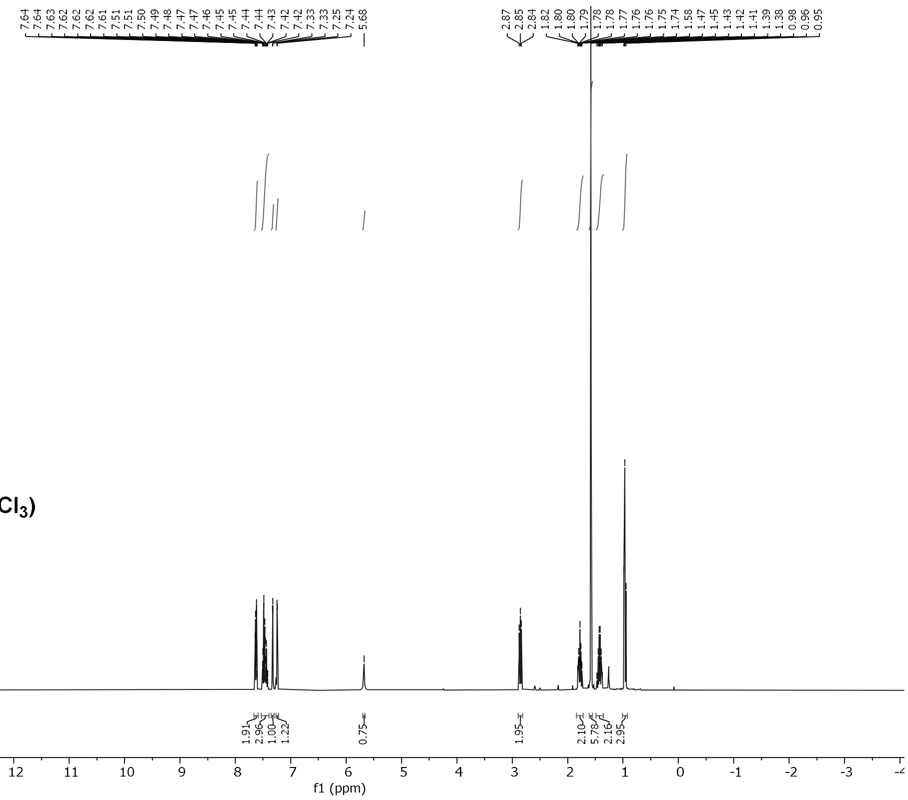

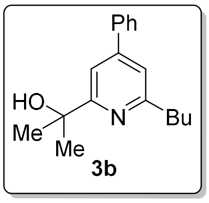

^{13}C NMR (101 MHz, $CDCl_3$)

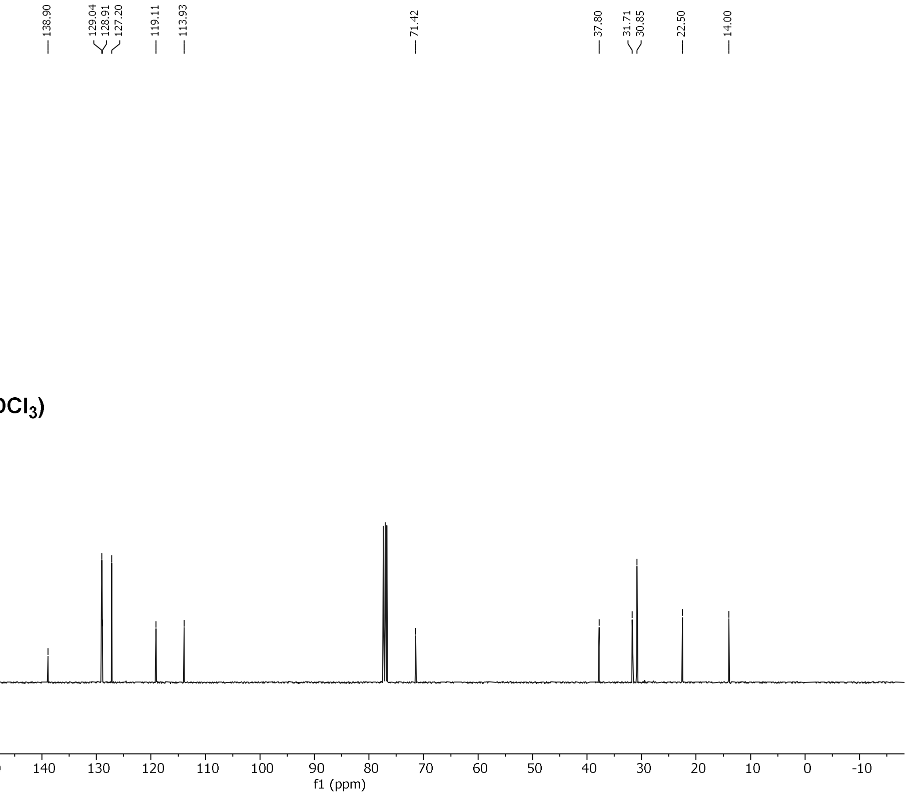

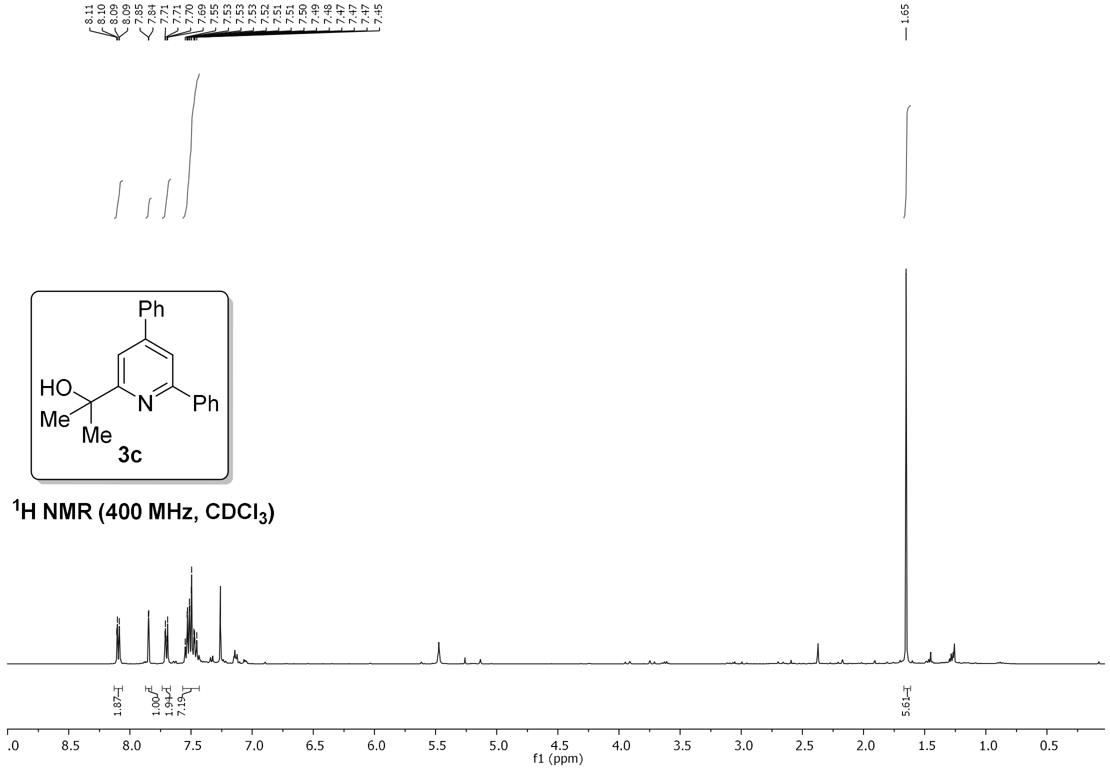
8.11
8.10
8.09
8.09
7.85
7.84
7.71
7.71
7.70
7.69
7.55
7.53
7.53
7.53
7.52
7.51
7.51
7.50
7.49
7.48
7.47
7.47
7.47
7.45
1.65
Ph
HO
N
Ph
Me
Me
3c
1H NMR (400 MHz, CDCl3)
1.87
1.00
1.94
7.19
5.61
f1 (ppm)

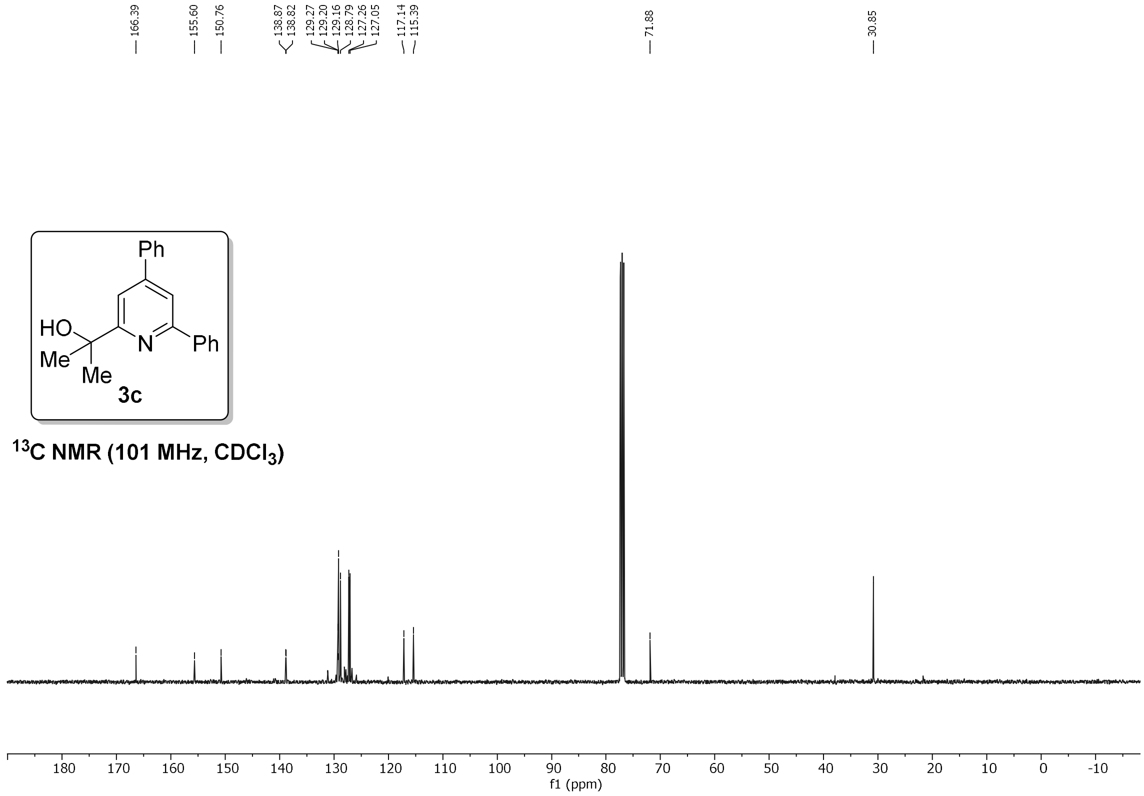
166.39
155.60
150.76
138.87
138.82
129.27
129.20
129.16
128.79
127.26
127.05
117.14
115.39
71.88
30.85
Ph
HO
N
Ph
Me
Me
3c
13C NMR (101 MHz, CDCl3)
f1 (ppm)

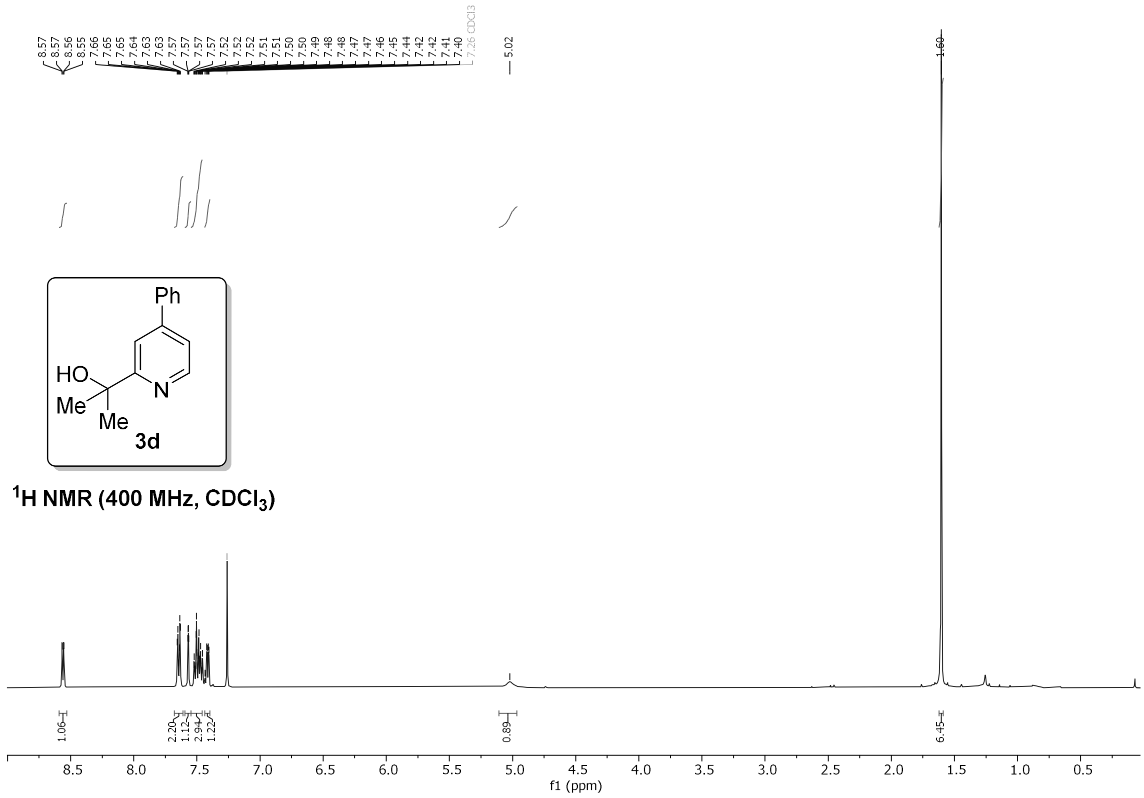

^{1}H NMR (400 MHz, $CDCl_3$)

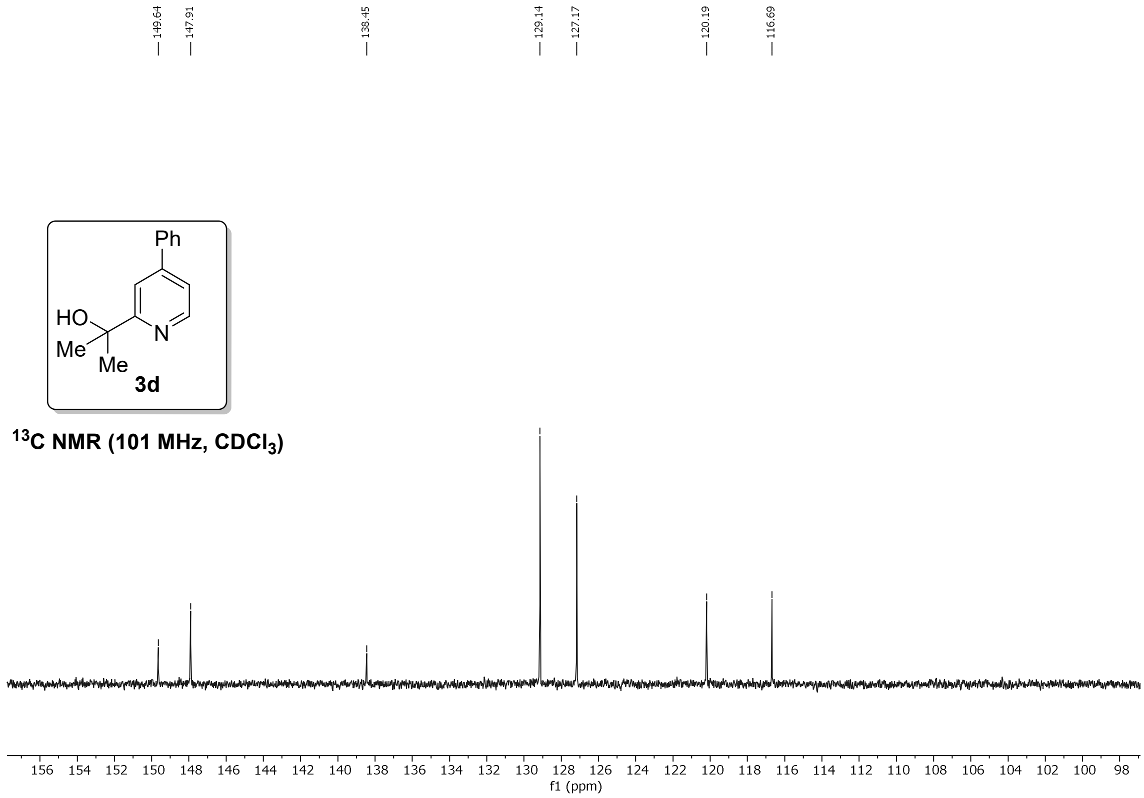

^{13}C NMR (101 MHz, $CDCl_3$)

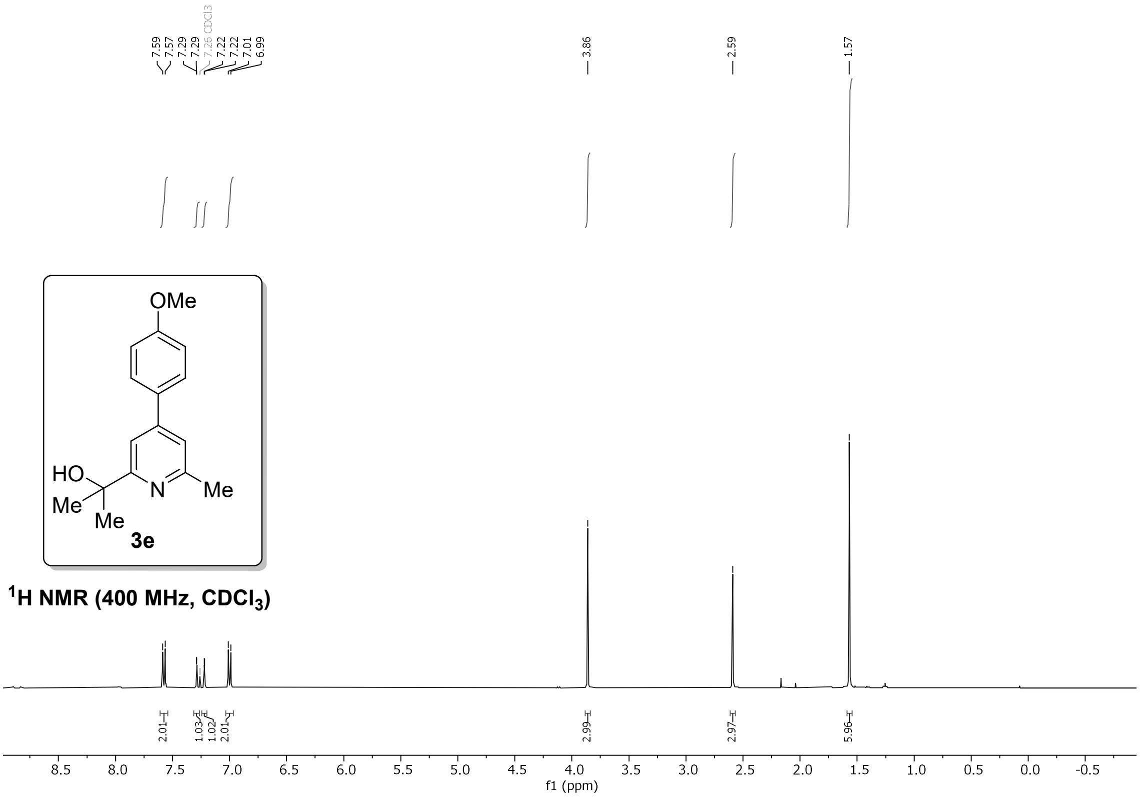

^{1}H NMR (400 MHz, CDCl$_3$)

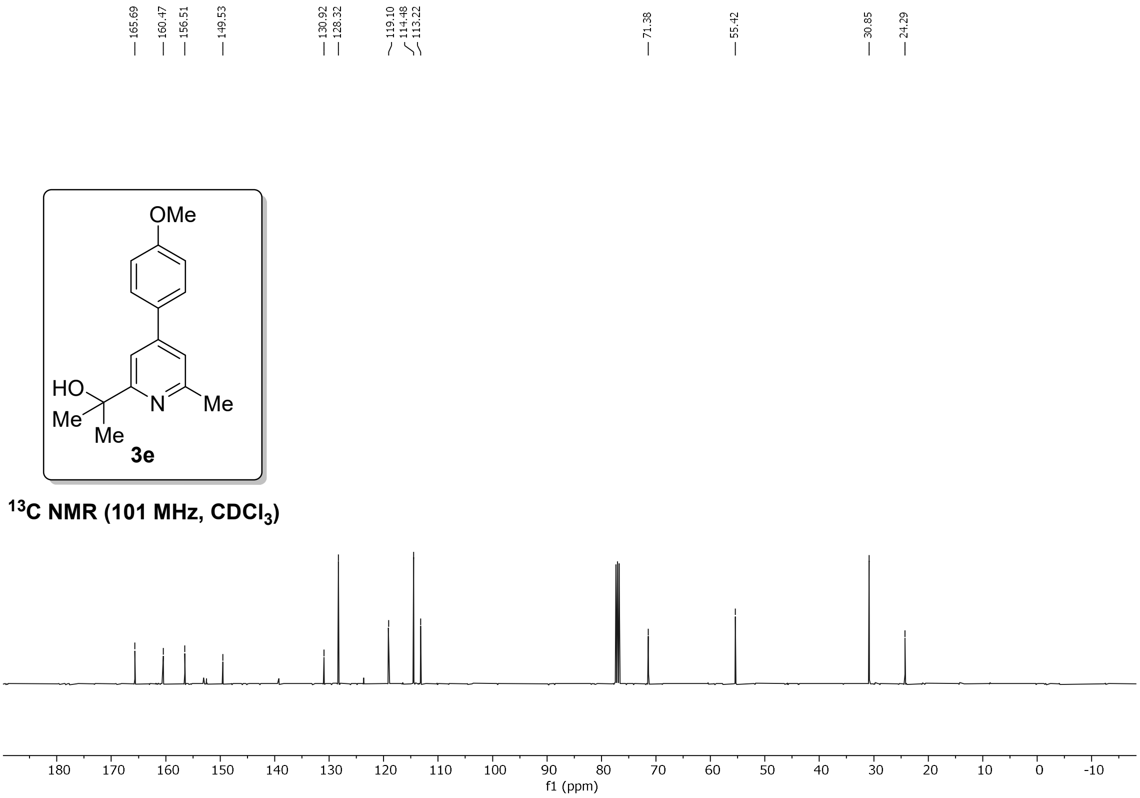

^{13}C NMR (101 MHz, CDCl$_3$)

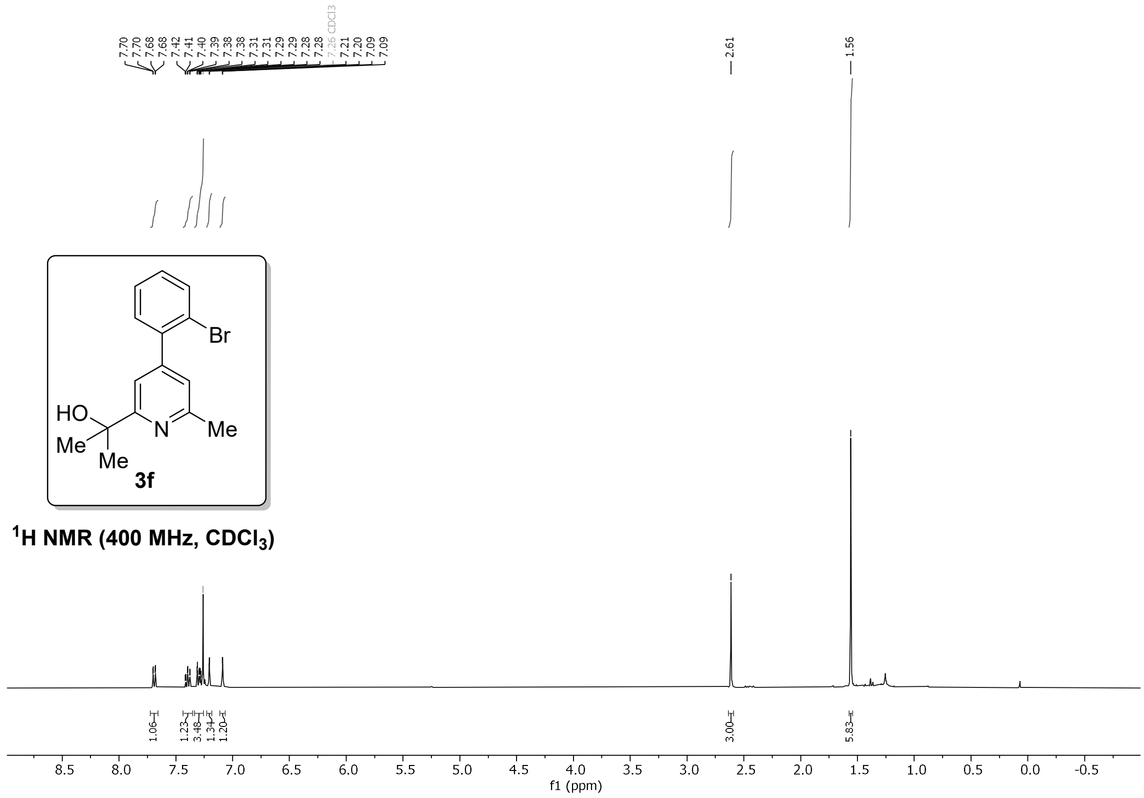
7.70
7.70
7.68
7.68
7.42
7.41
7.40
7.39
7.38
7.38
7.31
7.31
7.29
7.29
7.28
7.28
7.26 CDCl3
7.21
7.20
7.09
7.09
2.61
1.56
Br
HO
N
Me
Me
Me
3f
^{1}H NMR (400 MHz, $CDCl_3$)
1.06
1.23
3.48
1.34
1.20
3.00
5.83
8.5 8.0 7.5 7.0 6.5 6.0 5.5 5.0 4.5 4.0 3.5 3.0 2.5 2.0 1.5 1.0 0.5 0.0 -0.5
f1 (ppm)

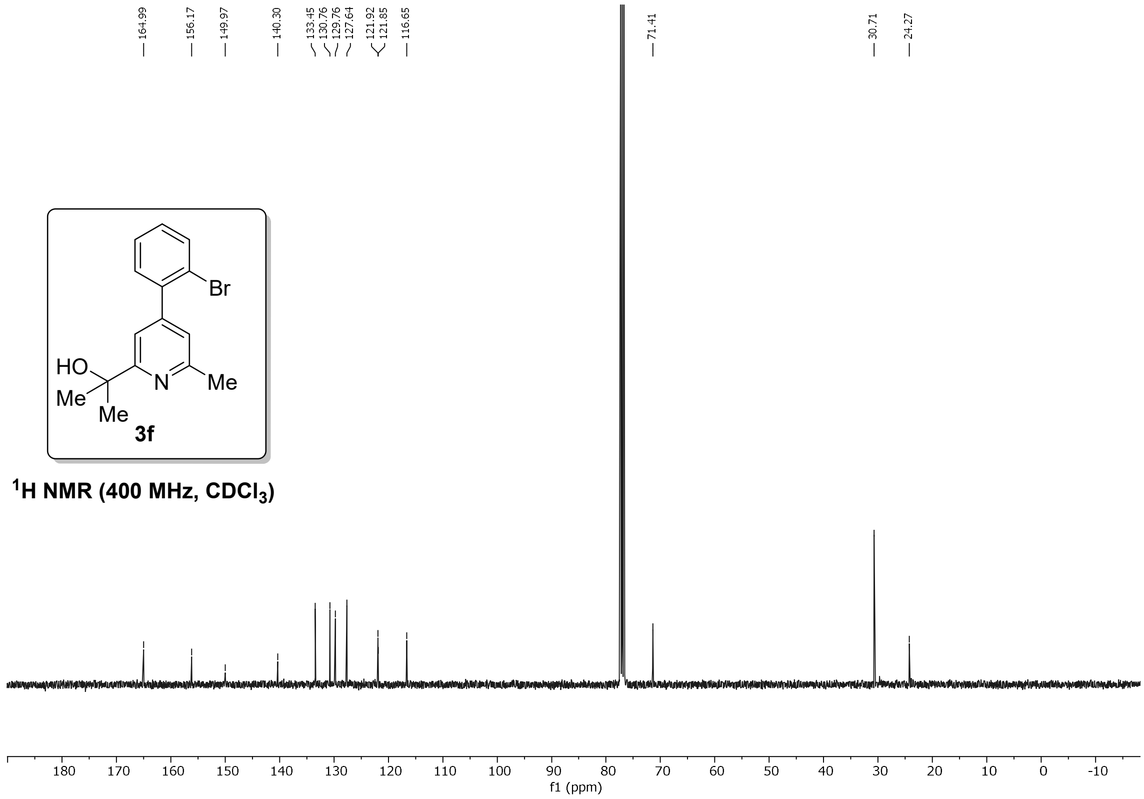
164.99
156.17
149.97
140.30
133.45
130.76
129.76
127.64
121.92
121.85
116.65
71.41
30.71
24.27
Br
HO
N
Me
Me
Me
3f
^{1}H NMR (400 MHz, $CDCl_3$)
180 170 160 150 140 130 120 110 100 90 80 70 60 50 40 30 20 10 0 -10
f1 (ppm)

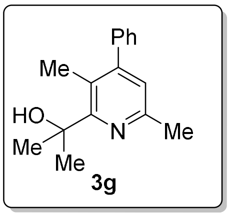

^{1}H NMR (400 MHz, $CDCl_3$)

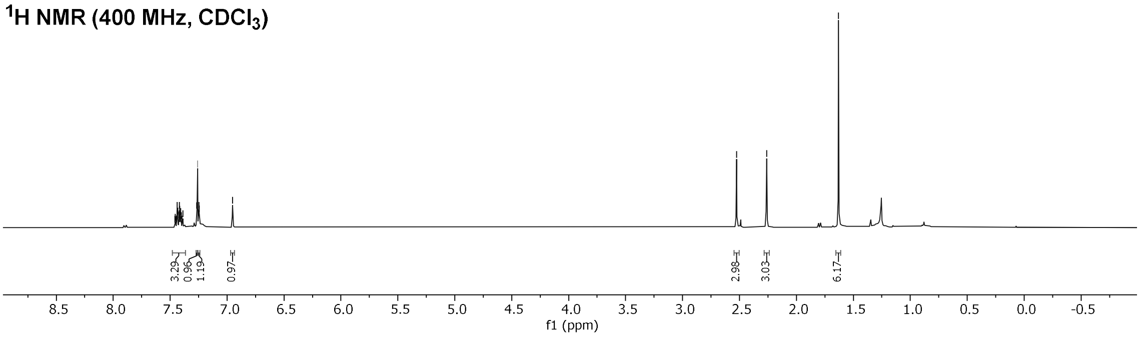

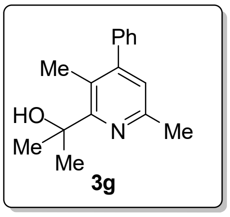

^{13}C NMR (101 MHz, $CDCl_3$)

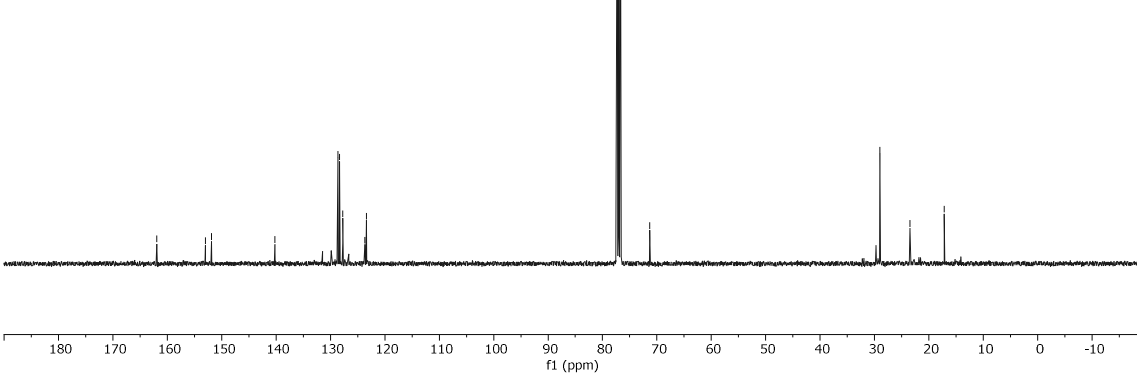

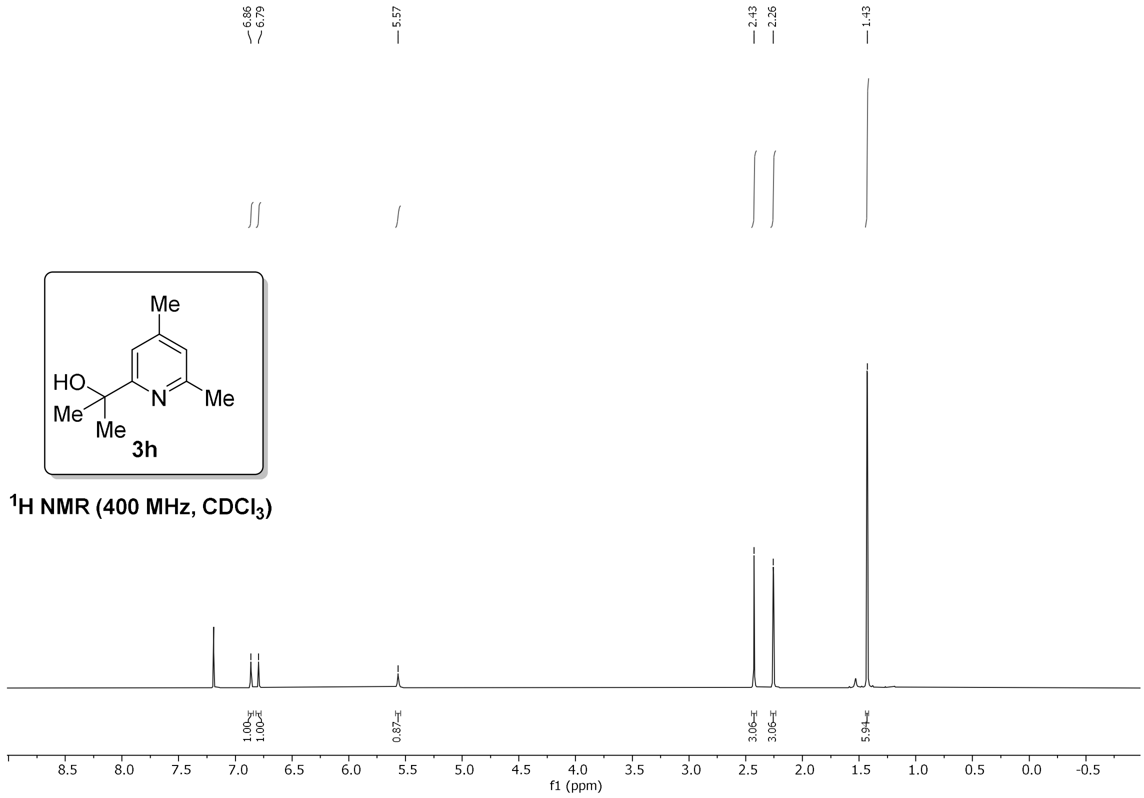

^{1}H NMR (400 MHz, $CDCl_3$)

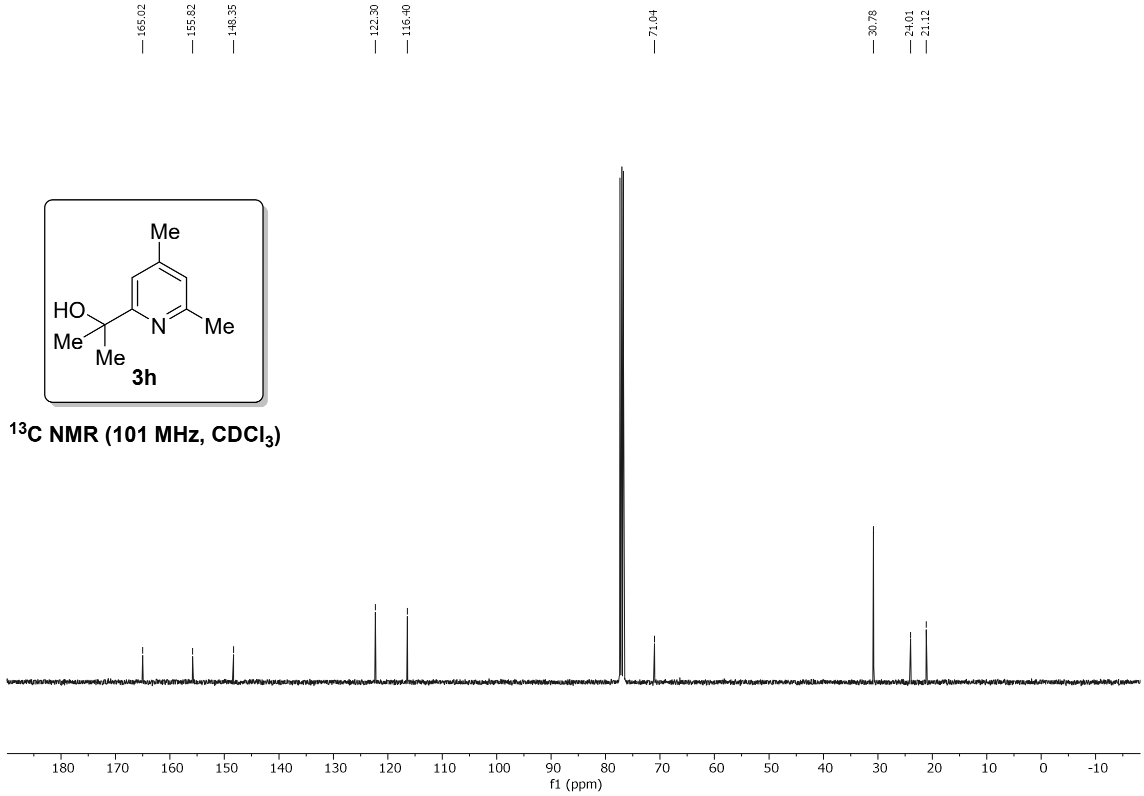

^{13}C NMR (101 MHz, $CDCl_3$)

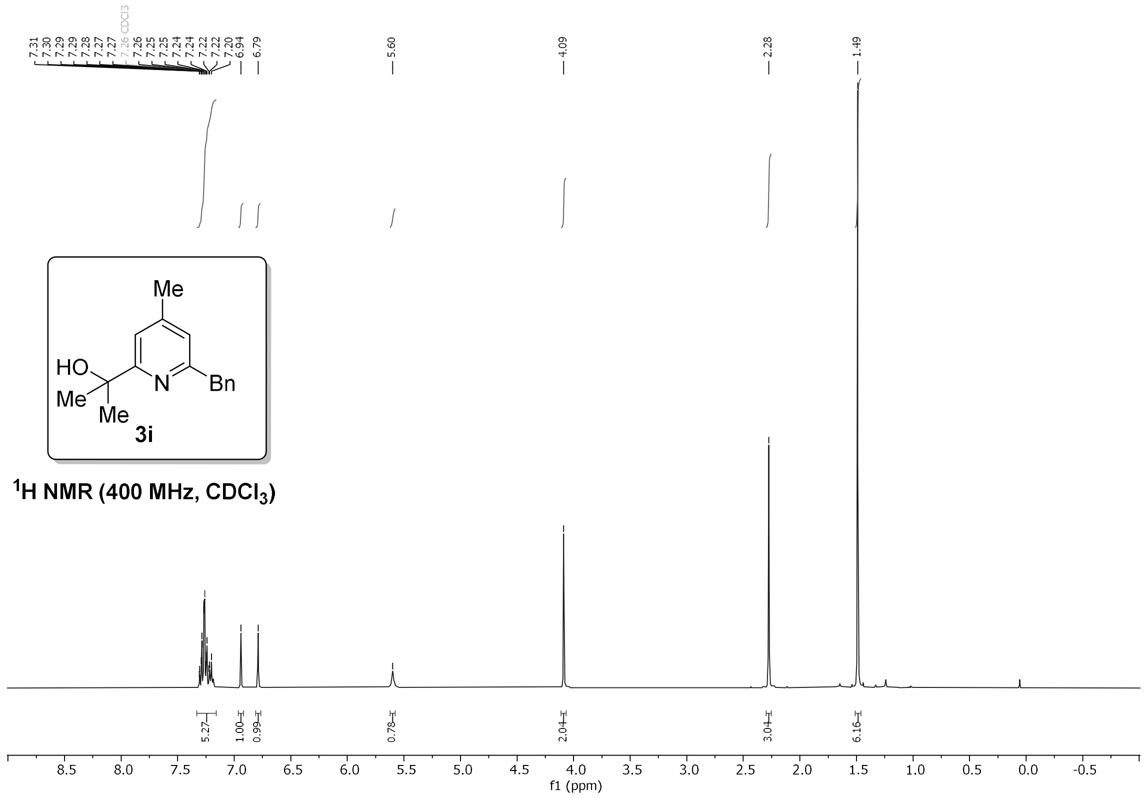
7.31 7.30 7.29 7.29 7.28 7.27 7.27 7.26 CDCl3 7.26 7.25 7.25 7.24 7.24 7.22 7.22 7.20 6.94 6.79
5.60
4.09
2.28
1.49
Me
HO
N
Bn
Me
Me
3i
1H NMR (400 MHz, CDCl3)
5.27 1.00 0.99 0.78 2.04 3.04 6.16
8.5 8.0 7.5 7.0 6.5 6.0 5.5 5.0 4.5 4.0 3.5 3.0 2.5 2.0 1.5 1.0 0.5 0.0 -0.5
f1 (ppm)

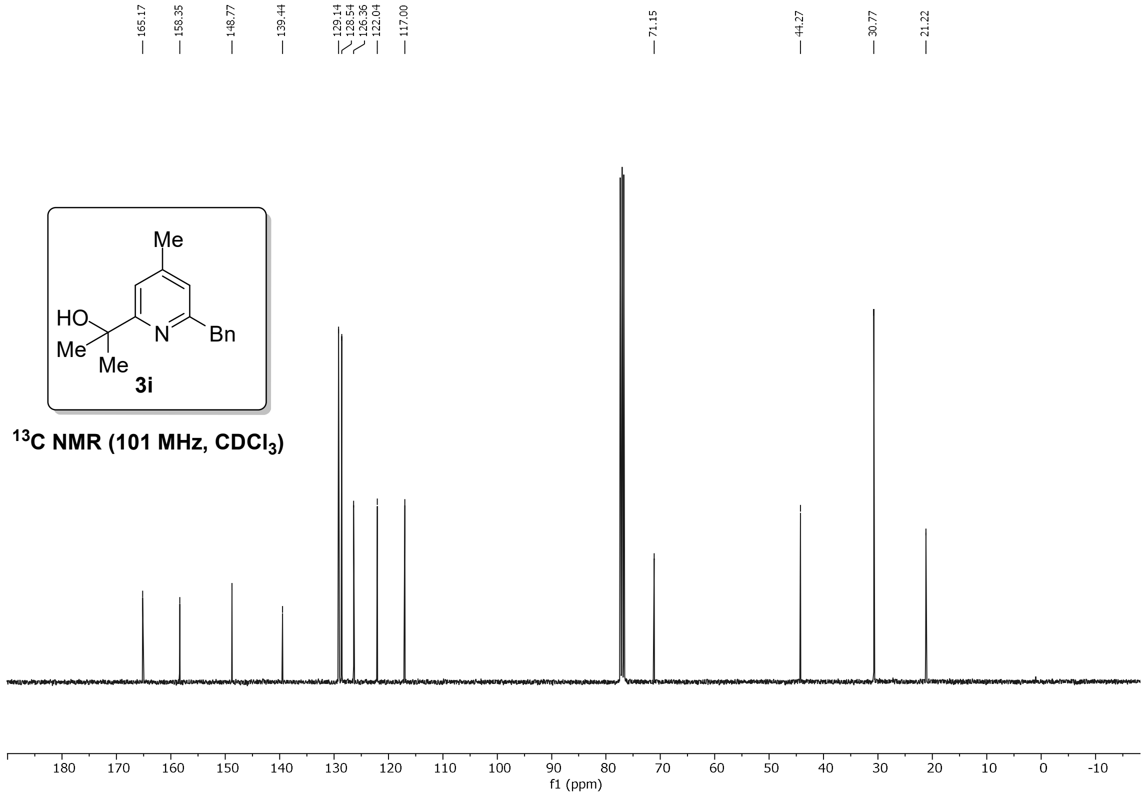
165.17
158.35
148.77
139.44
129.14 128.54 126.36 122.04
117.00
71.15
44.27
30.77
21.22
Me
HO
N
Bn
Me
Me
3i
13C NMR (101 MHz, CDCl3)
180 170 160 150 140 130 120 110 100 90 80 70 60 50 40 30 20 10 0 -10
f1 (ppm)

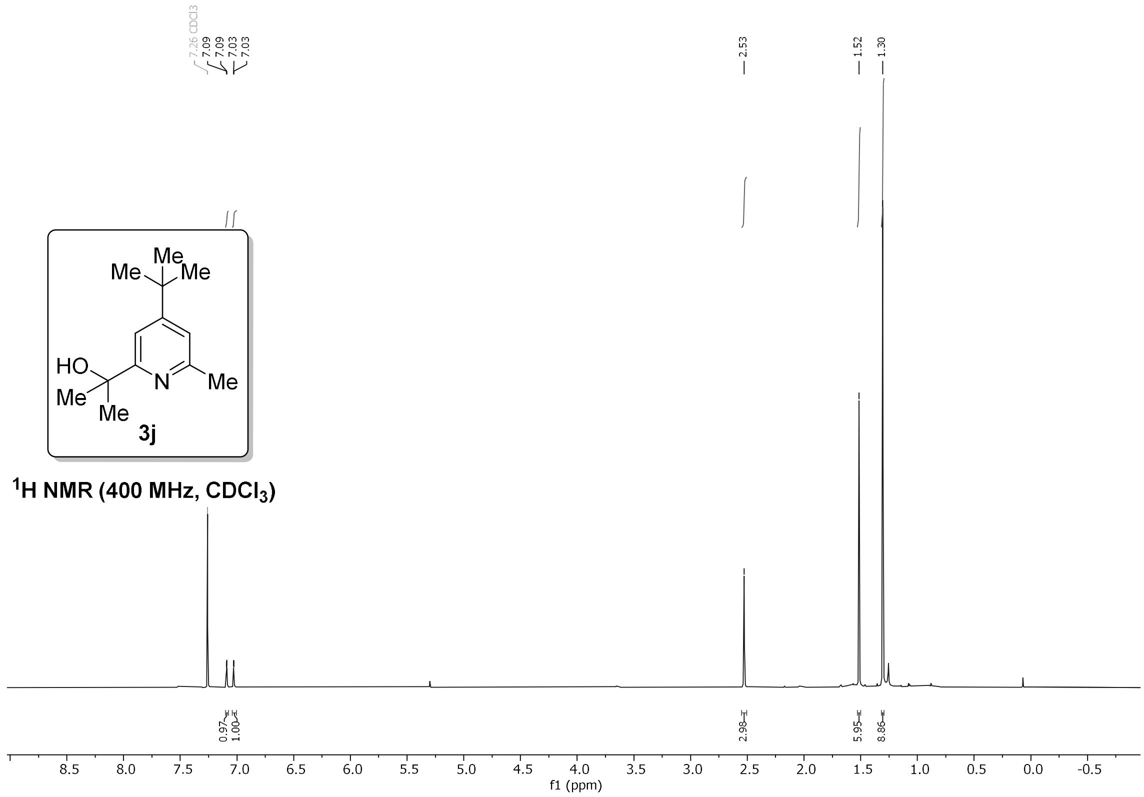

^{1}H NMR (400 MHz, $CDCl_3$)

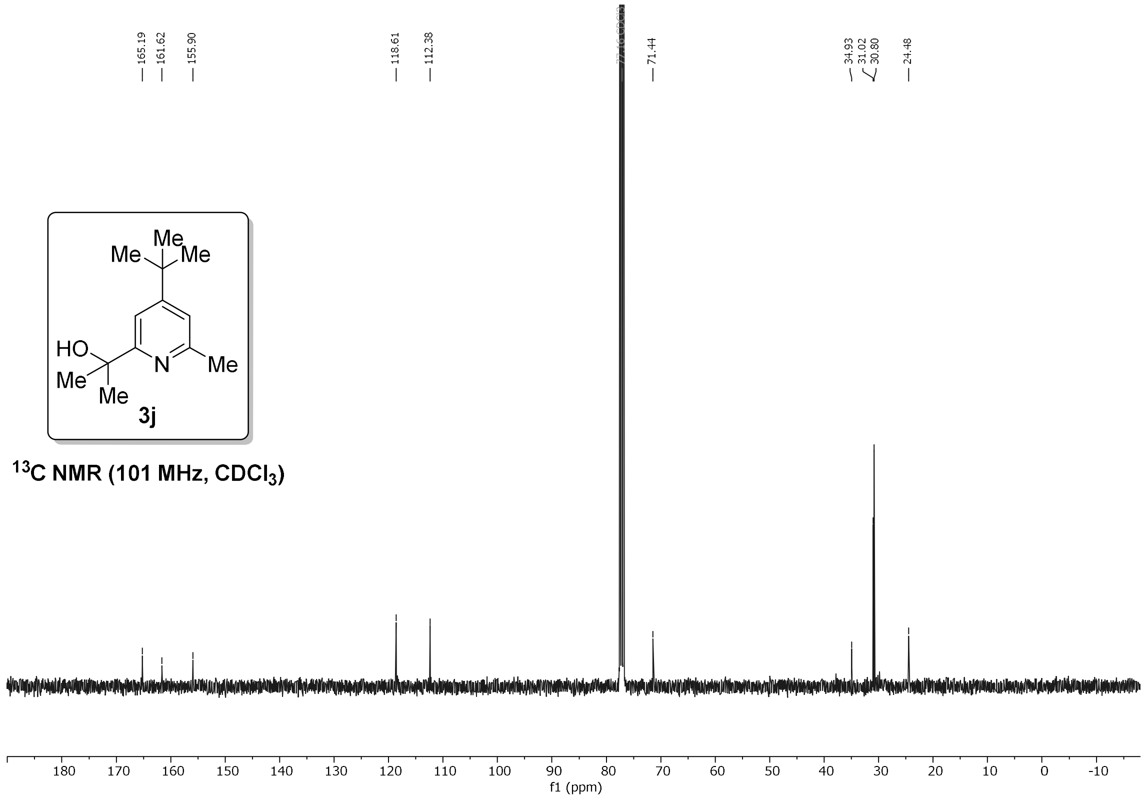

^{13}C NMR (101 MHz, $CDCl_3$)

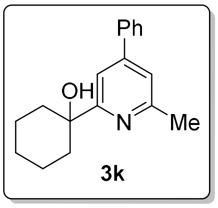

^{1}H NMR (400 MHz, $CDCl_3$)

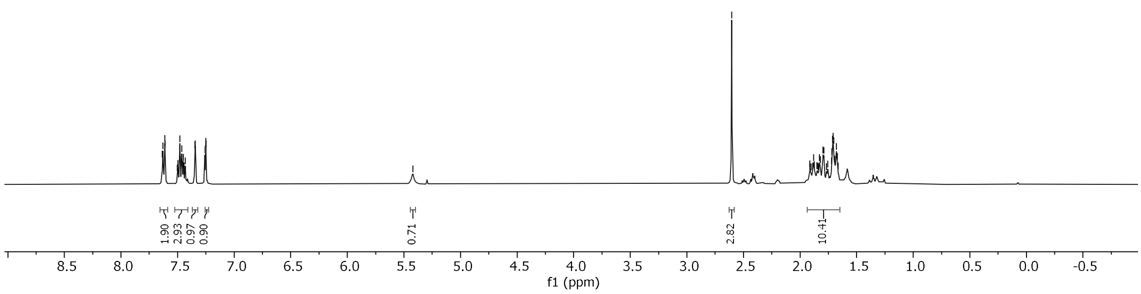

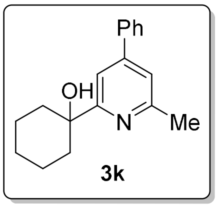

^{13}C NMR (101 MHz, $CDCl_3$)

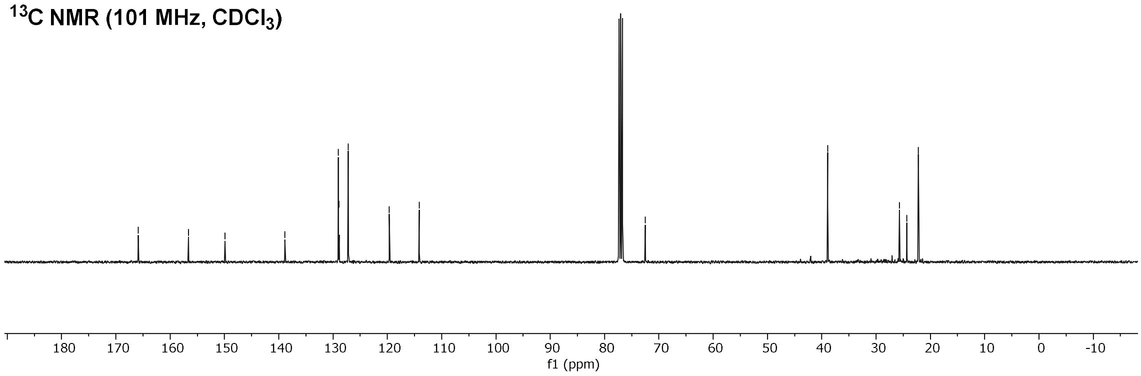

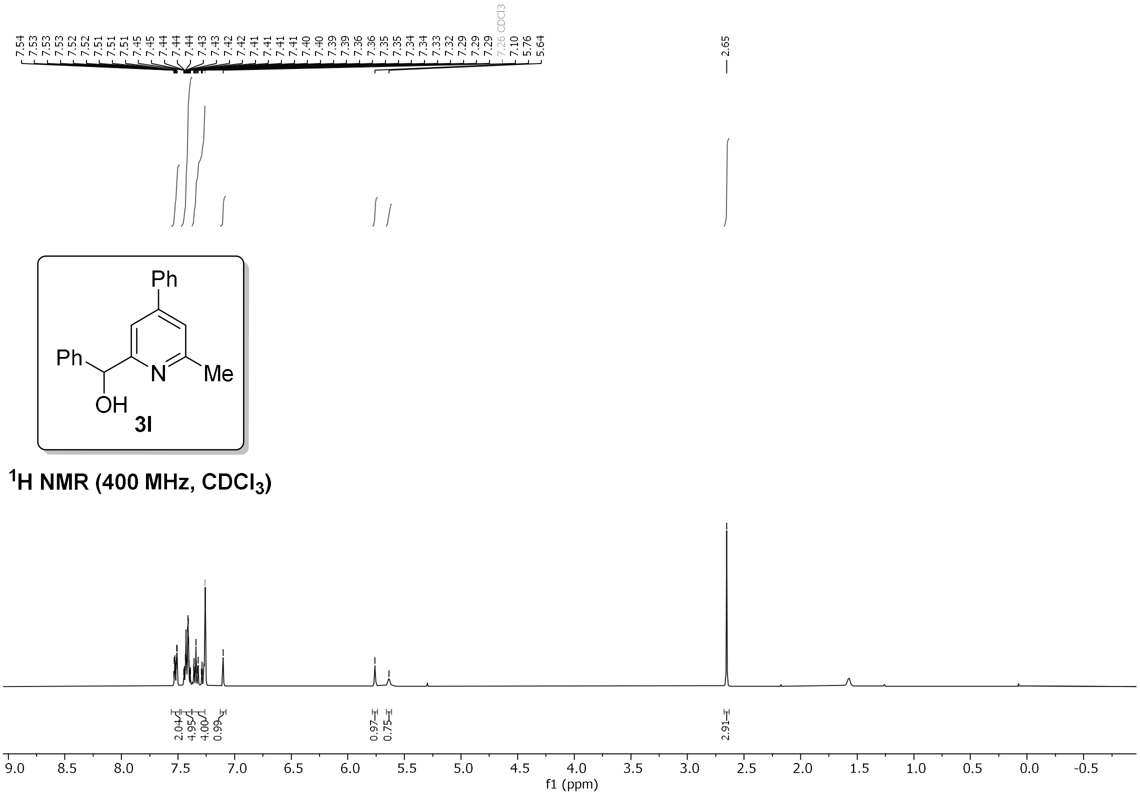

^{1}H NMR (400 MHz, $CDCl_3$)

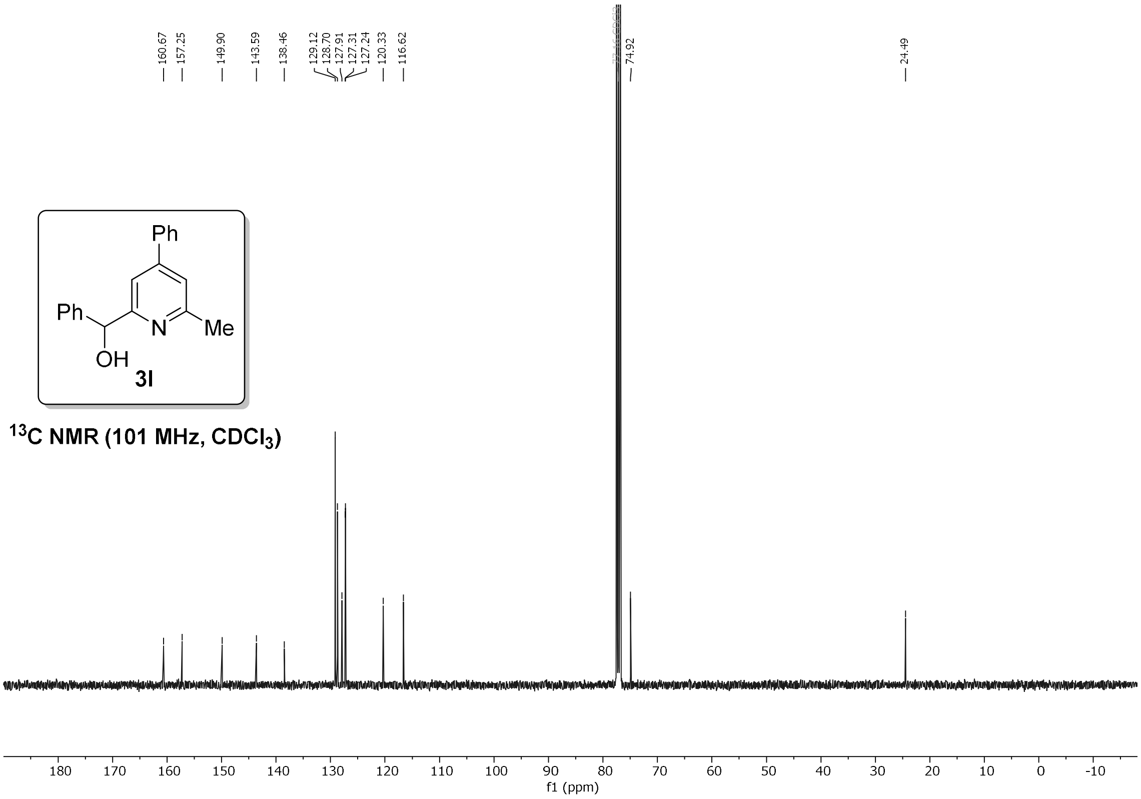

^{13}C NMR (101 MHz, $CDCl_3$)

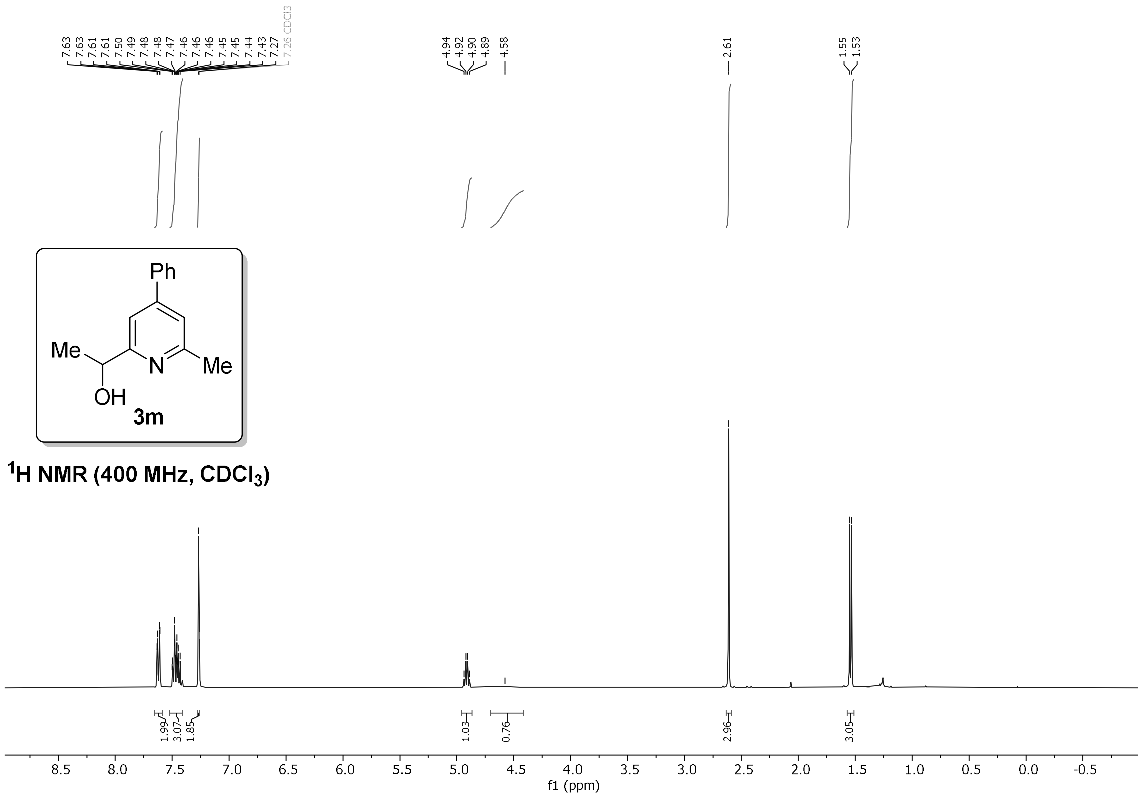

^{1}H NMR (400 MHz, $CDCl_3$)

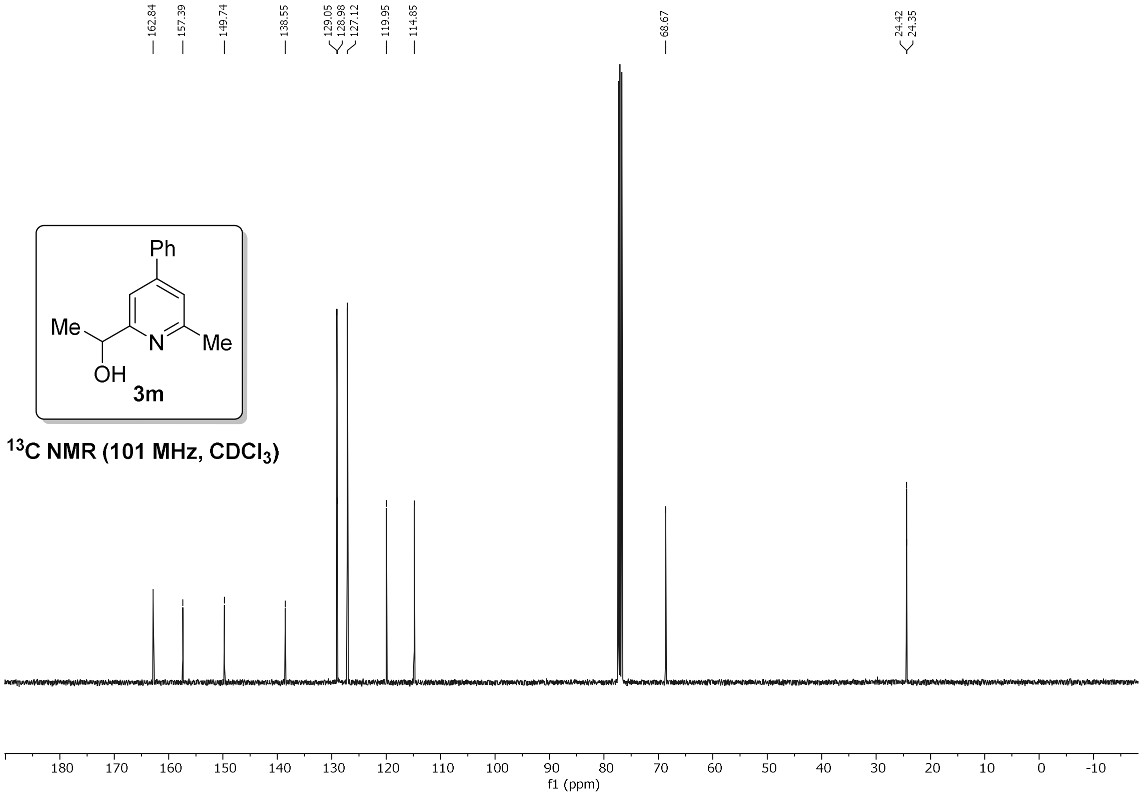

^{13}C NMR (101 MHz, $CDCl_3$)

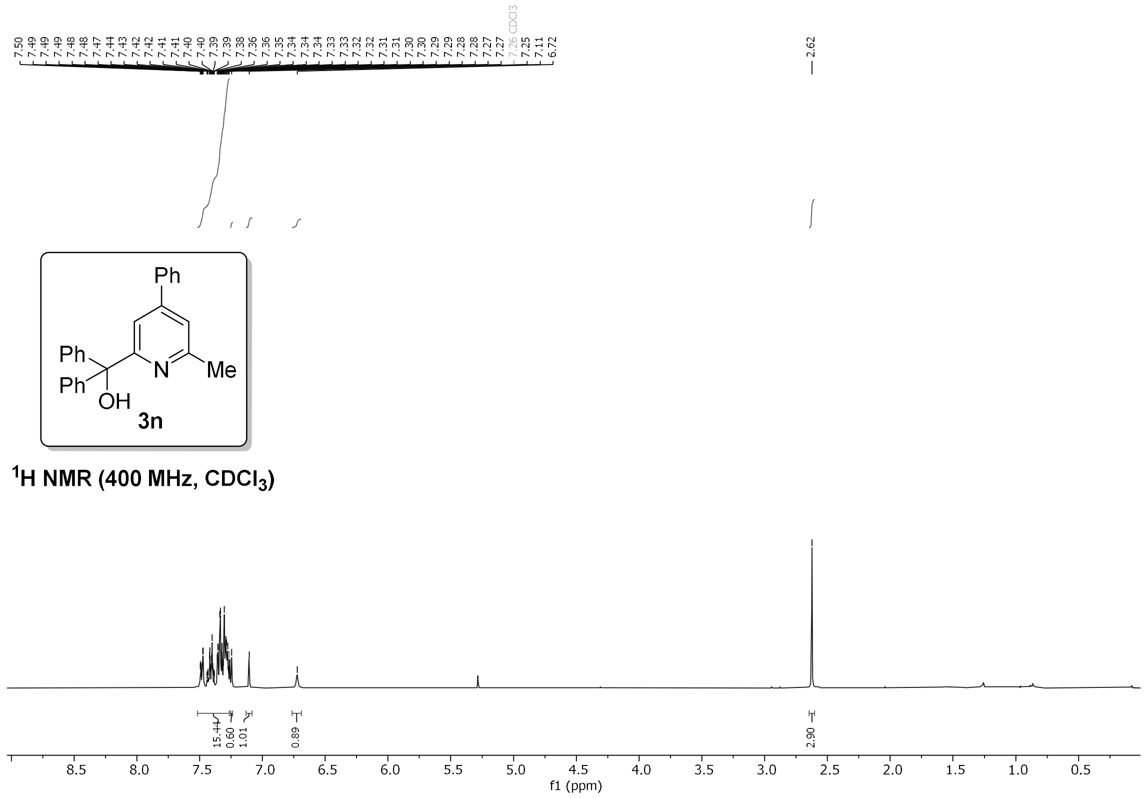

^{1}H NMR (400 MHz, $CDCl_3$)

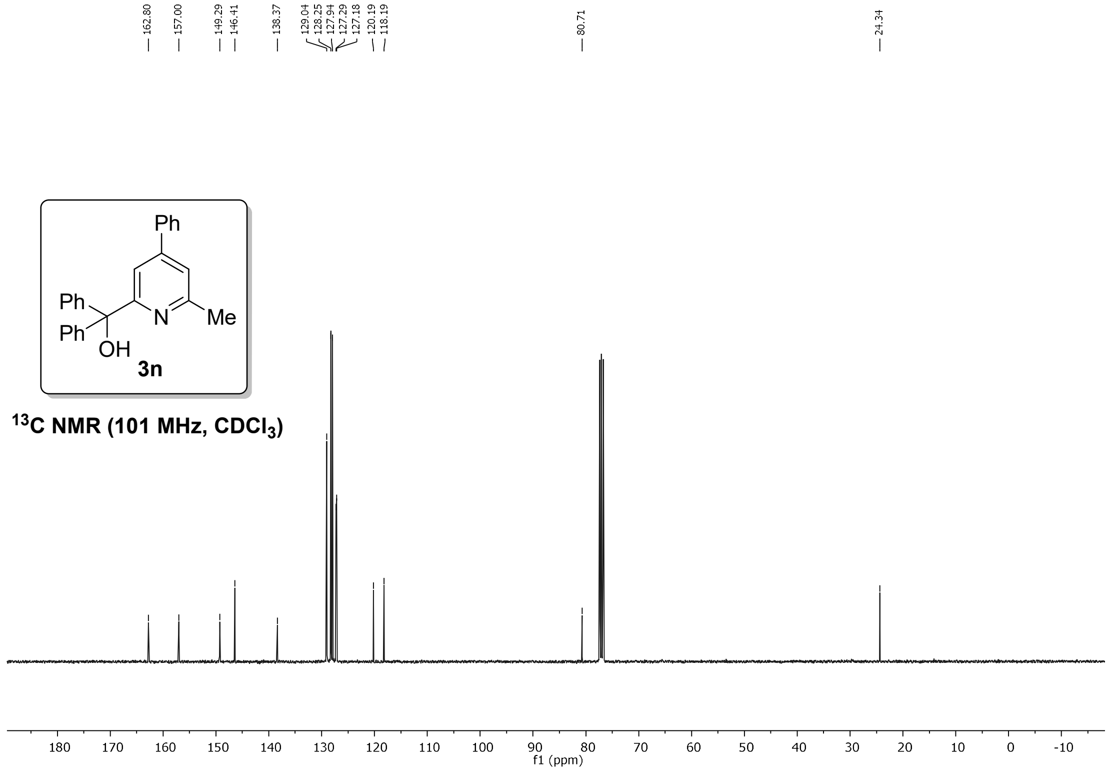

^{13}C NMR (101 MHz, $CDCl_3$)

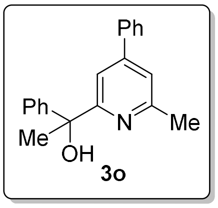

^{1}H NMR (400 MHz, $CDCl_3$)

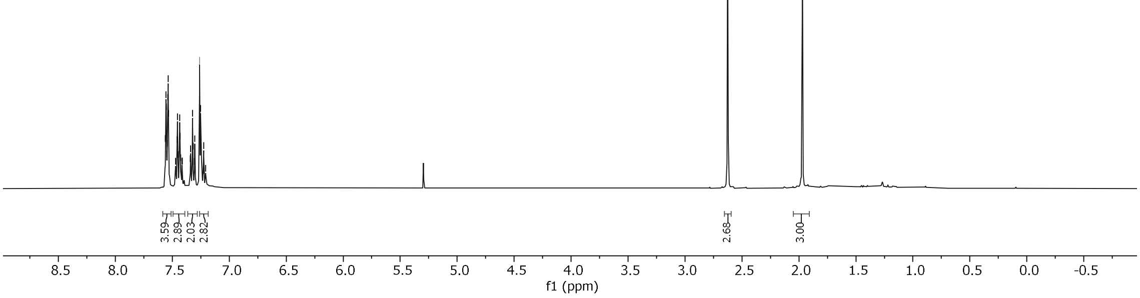

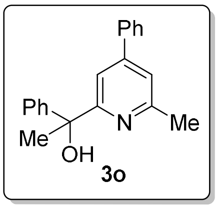

^{13}C NMR (101 MHz, $CDCl_3$)

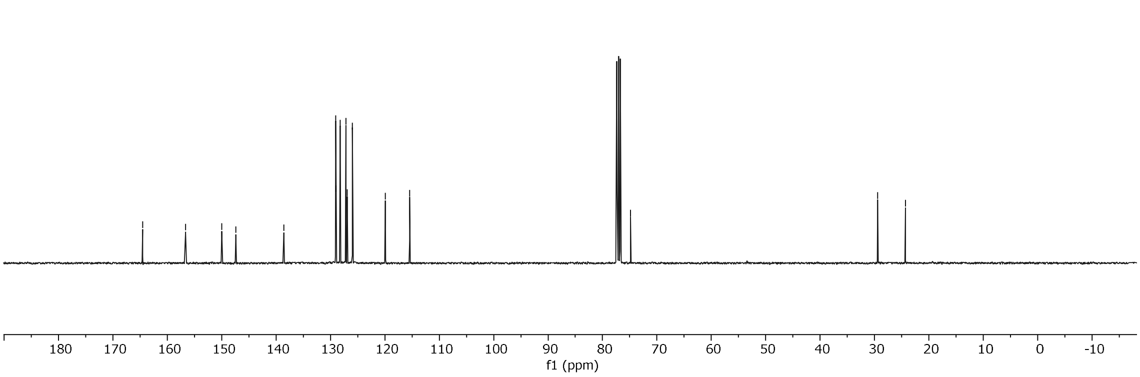

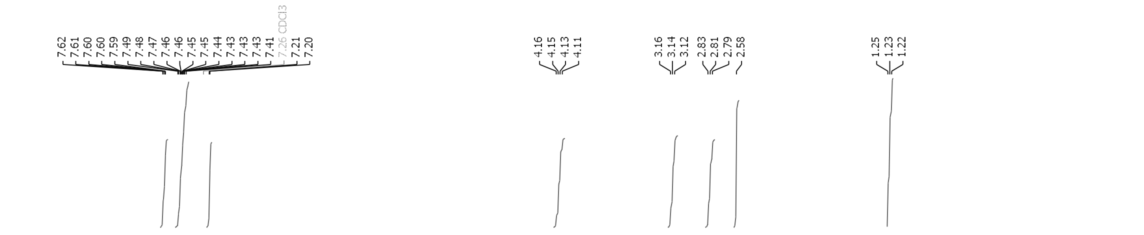

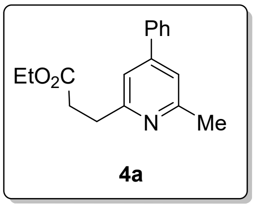

^{1}H NMR (400 MHz, $CDCl_3$)

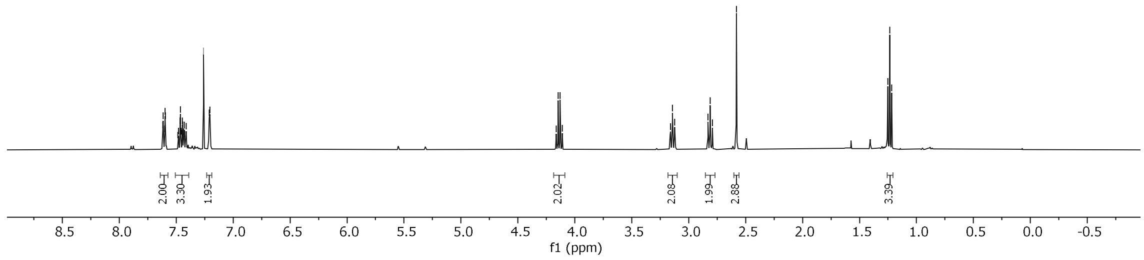

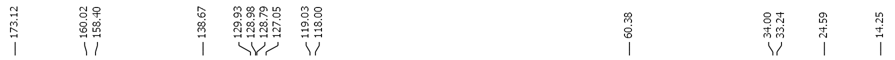

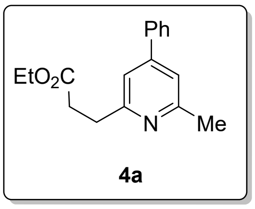

^{13}C NMR (101 MHz, $CDCl_3$)

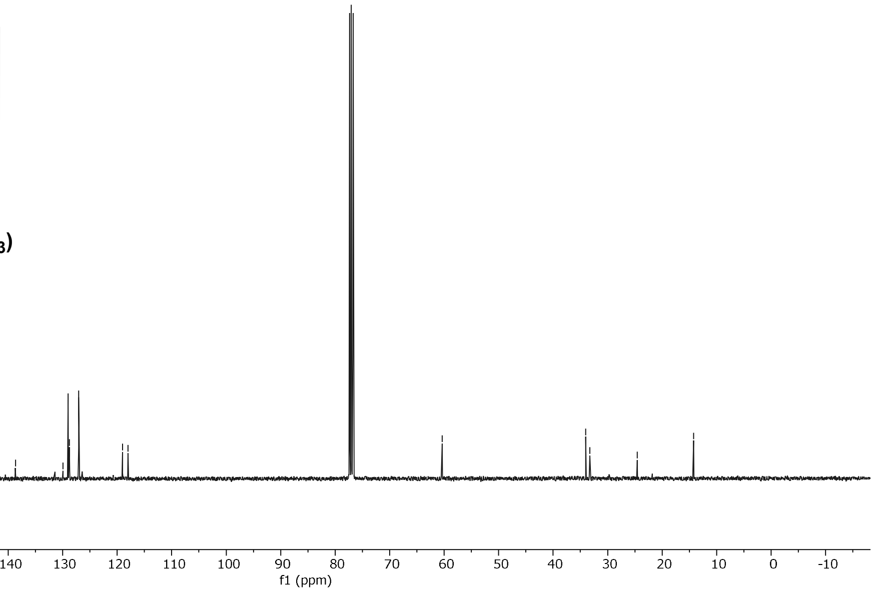

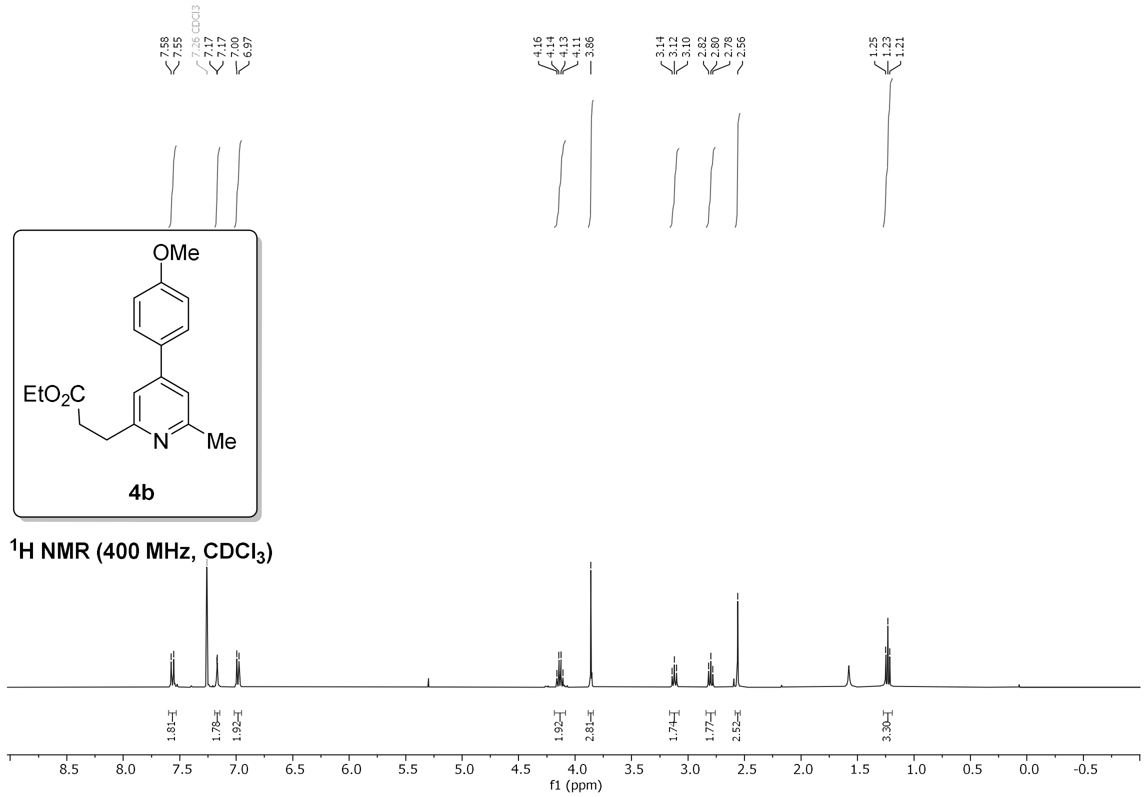
7.58
7.55
7.26 CDCl3
7.17
7.17
7.00
6.97
4.16
4.14
4.13
4.11
3.86
3.14
3.12
3.10
2.82
2.80
2.78
2.56
1.25
1.23
1.21
OMe
EtO2C
N
Me
4b
1H NMR (400 MHz, CDCl3)
1.81
1.78
1.92
1.92
2.81
1.74
1.77
2.52
3.30
8.5 8.0 7.5 7.0 6.5 6.0 5.5 5.0 4.5 4.0 3.5 3.0 2.5 2.0 1.5 1.0 0.5 0.0 -0.5
f1 (ppm)

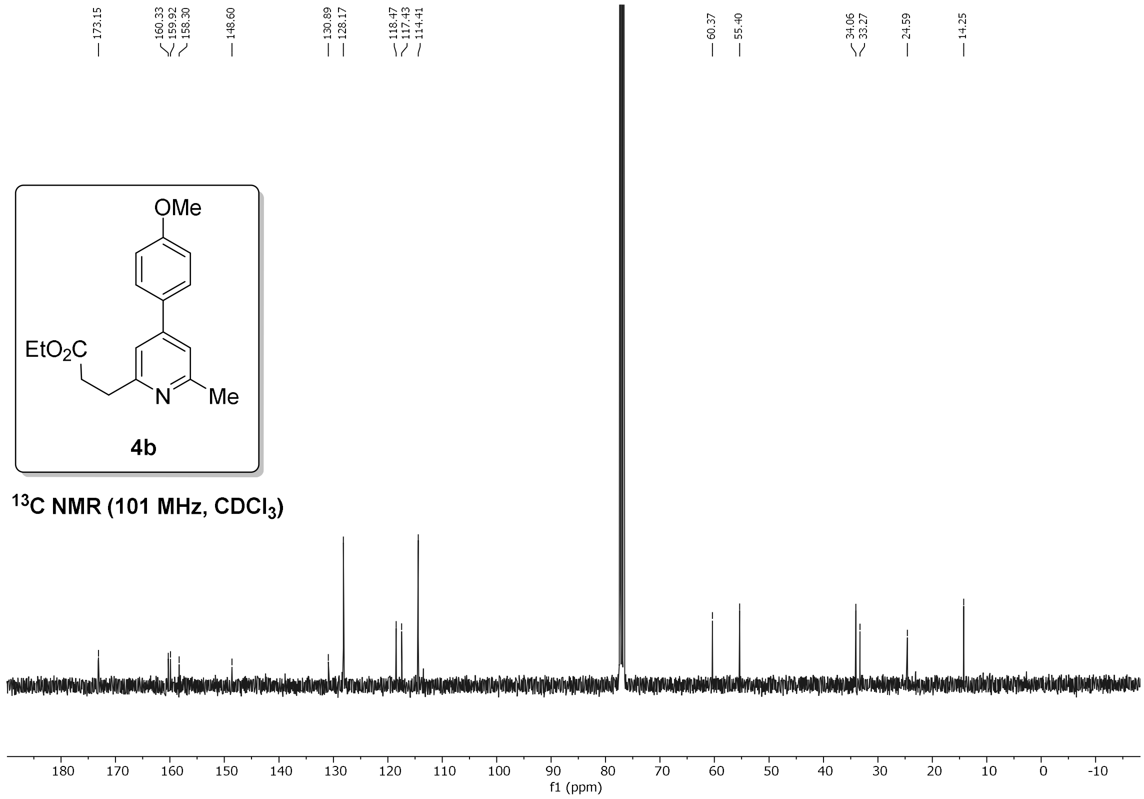
173.15
160.33
159.92
158.30
148.60
130.89
128.17
118.47
117.43
114.41
60.37
55.40
34.06
33.27
24.59
14.25
OMe
EtO2C
N
Me
4b
13C NMR (101 MHz, CDCl3)
180 170 160 150 140 130 120 110 100 90 80 70 60 50 40 30 20 10 0 -10
f1 (ppm)

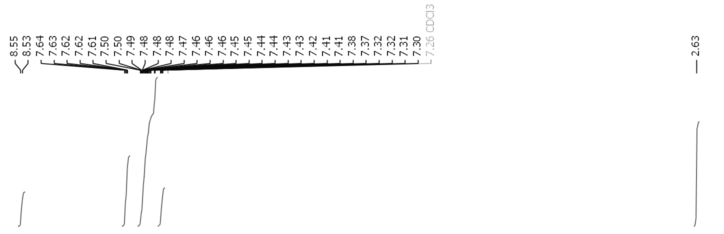

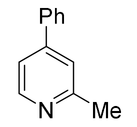

5a

^{1}H NMR (400 MHz, CDCl$_3$)

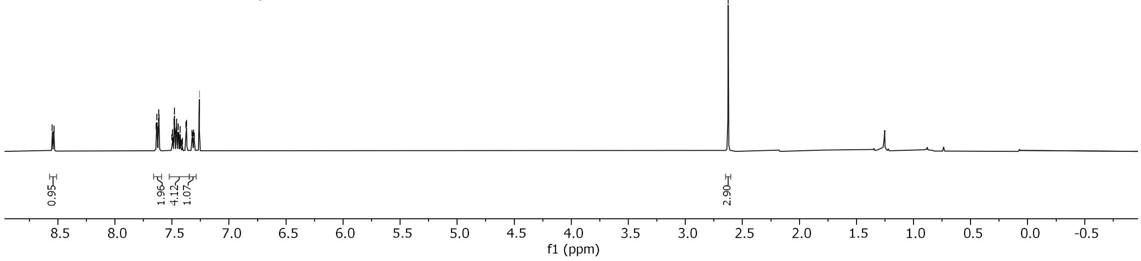

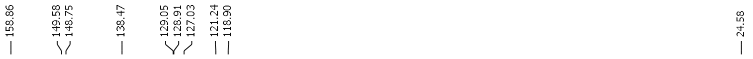

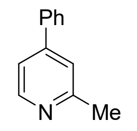

5a

^{13}C NMR (101 MHz, CDCl$_3$)

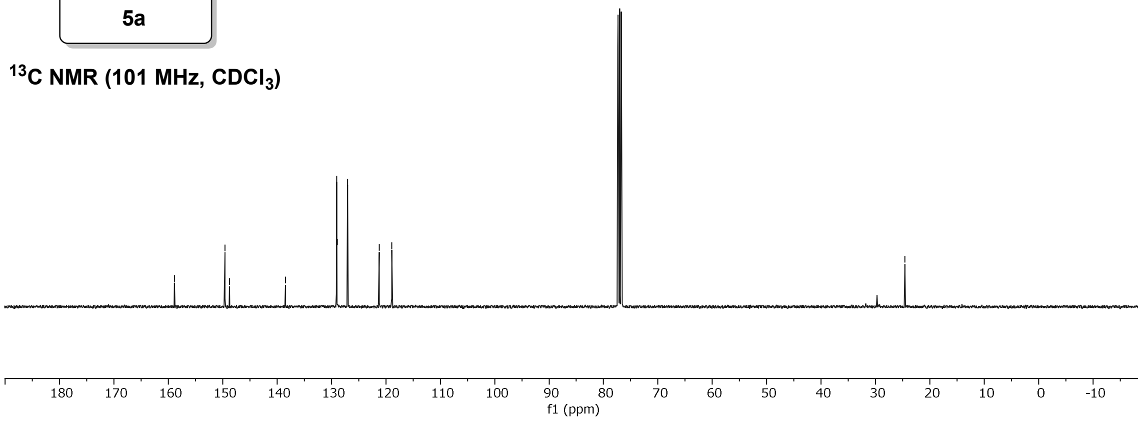

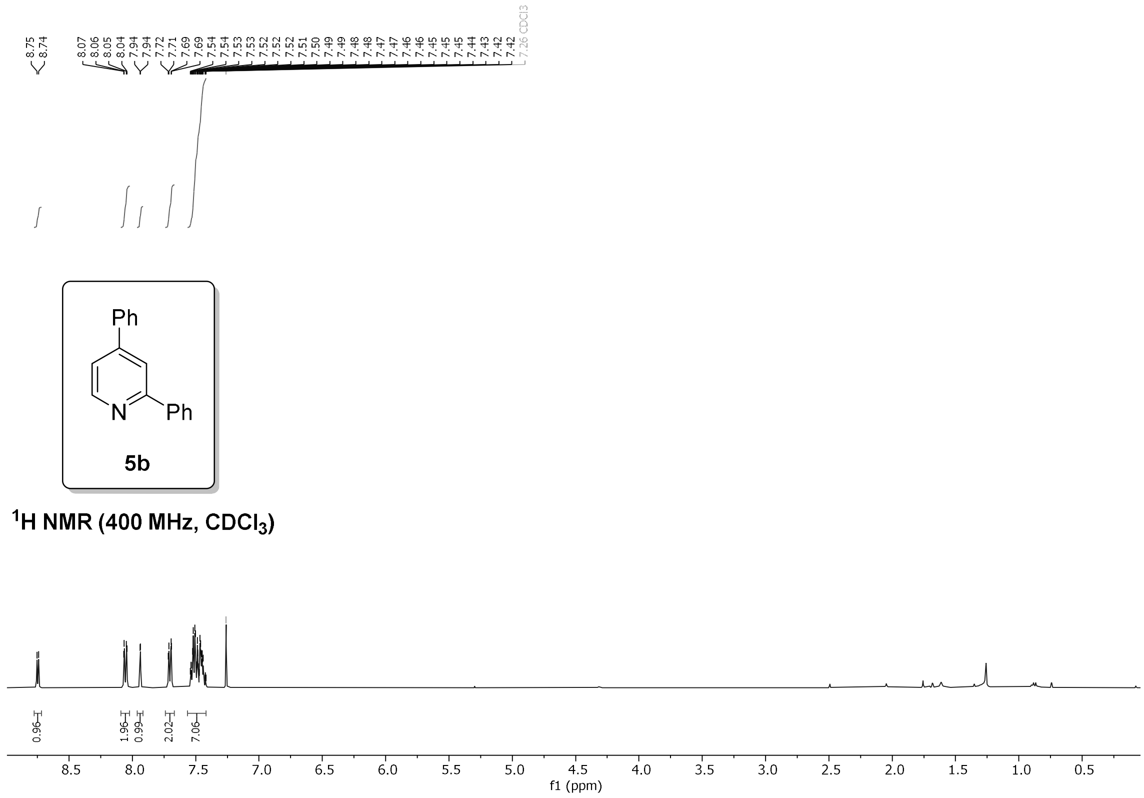

5b

^{1}H NMR (400 MHz, $CDCl_3$)

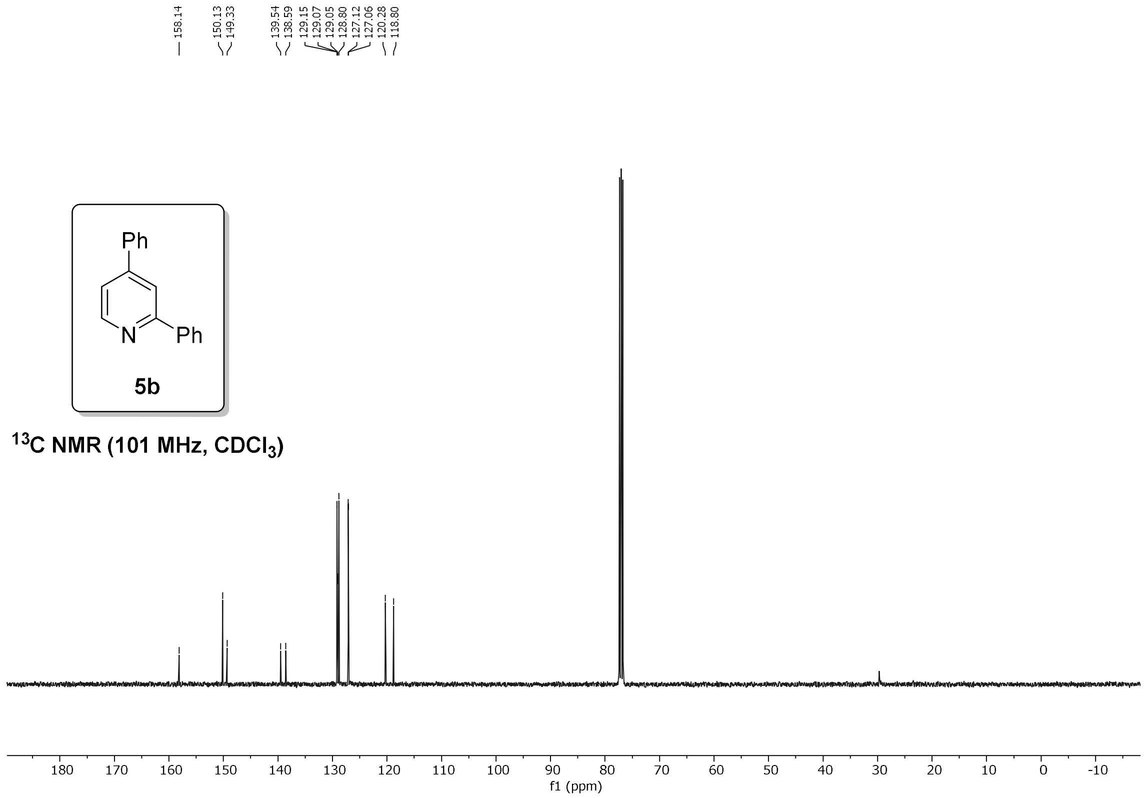

5b

^{13}C NMR (101 MHz, $CDCl_3$)

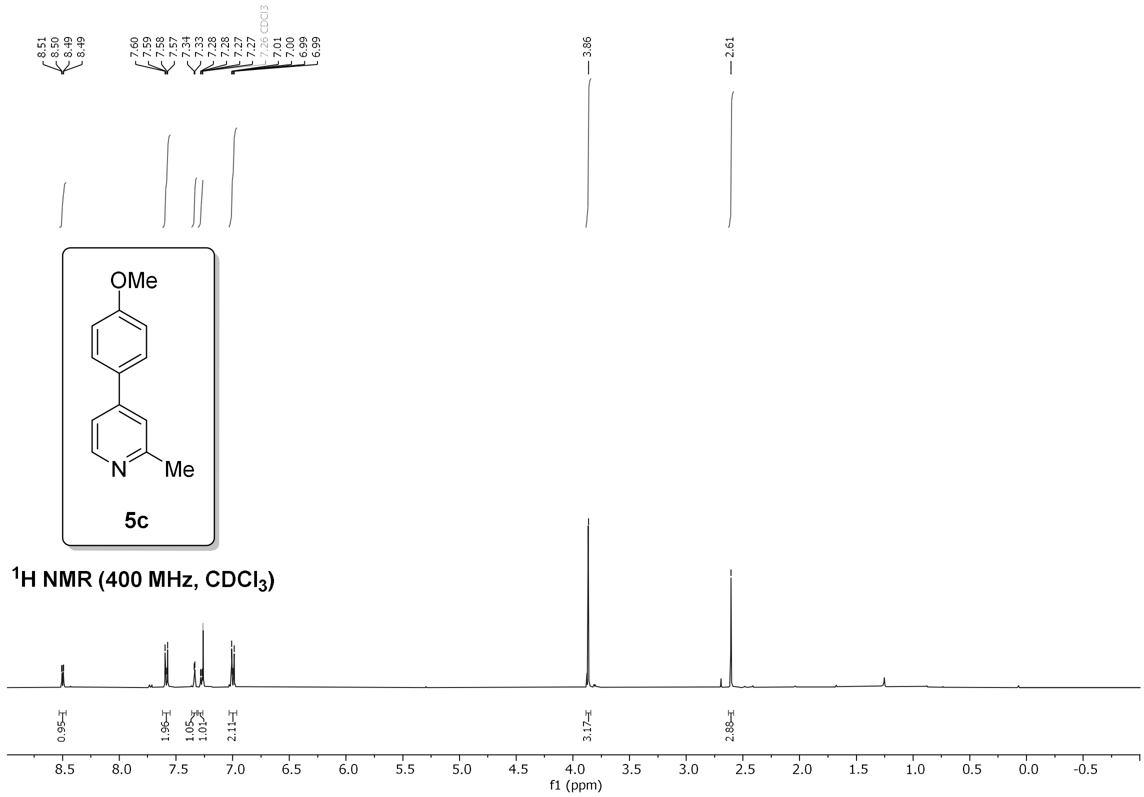

8.51
8.50
8.49
8.49
7.60
7.59
7.58
7.57
7.34
7.33
7.28
7.28
7.27
7.27
7.26 CDCl3
7.01
7.00
6.99
6.99
3.86
2.61
OMe
N
Me
5c
^{1}H NMR (400 MHz, $CDCl_3$)
0.95
1.96
1.05
1.01
2.11
3.17
2.88
f1 (ppm)

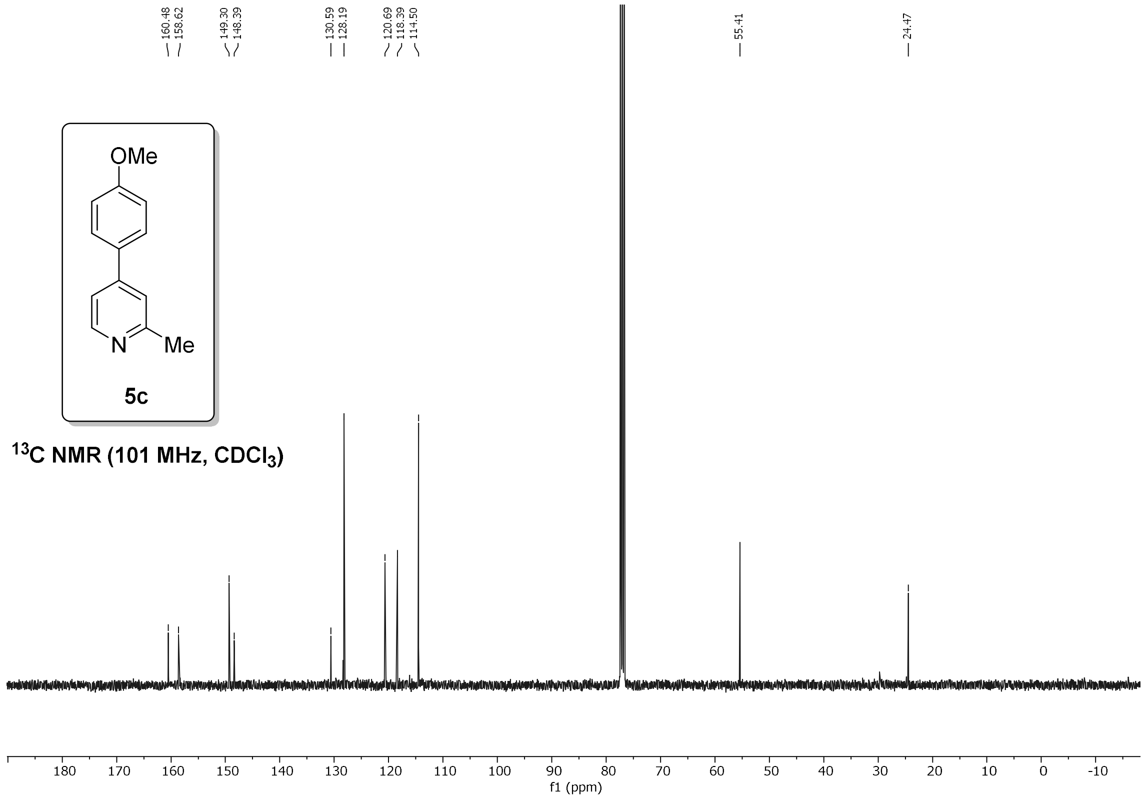

160.48
158.62
149.30
148.39
130.59
128.19
120.69
118.39
114.50
55.41
24.47
OMe
N
Me
5c
^{13}C NMR (101 MHz, $CDCl_3$)
f1 (ppm)

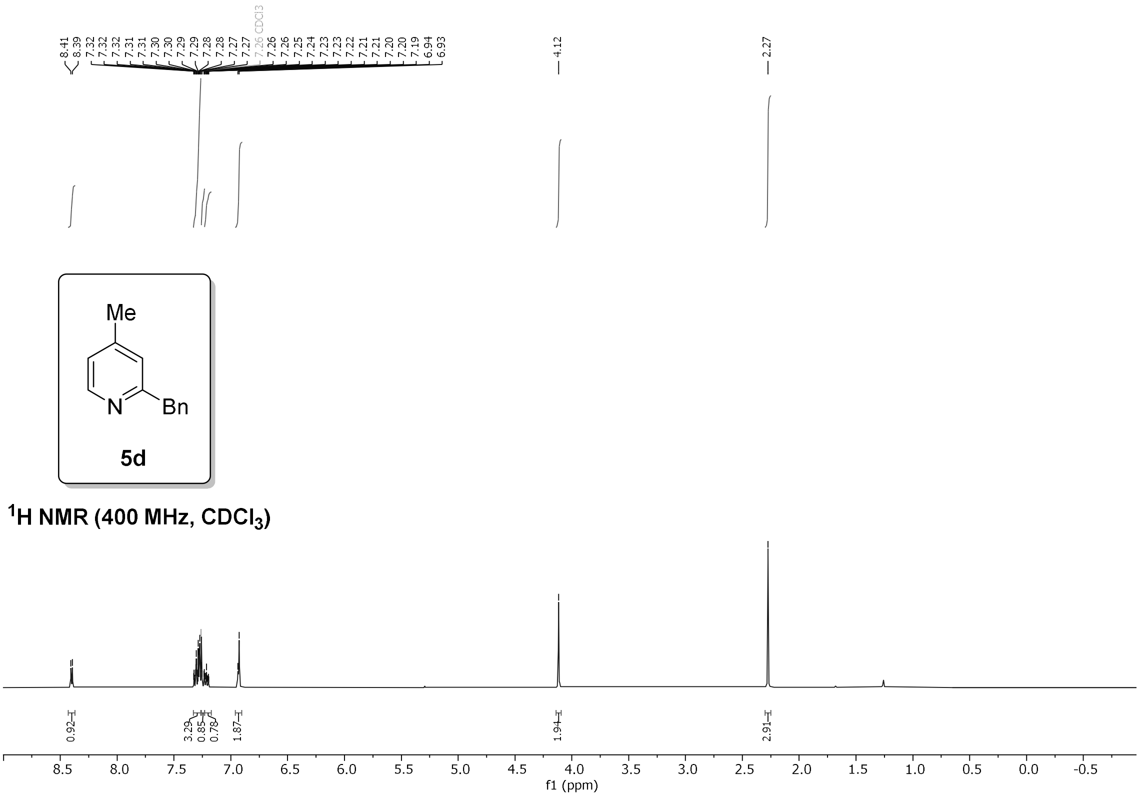

^{1}H NMR (400 MHz, $CDCl_3$)

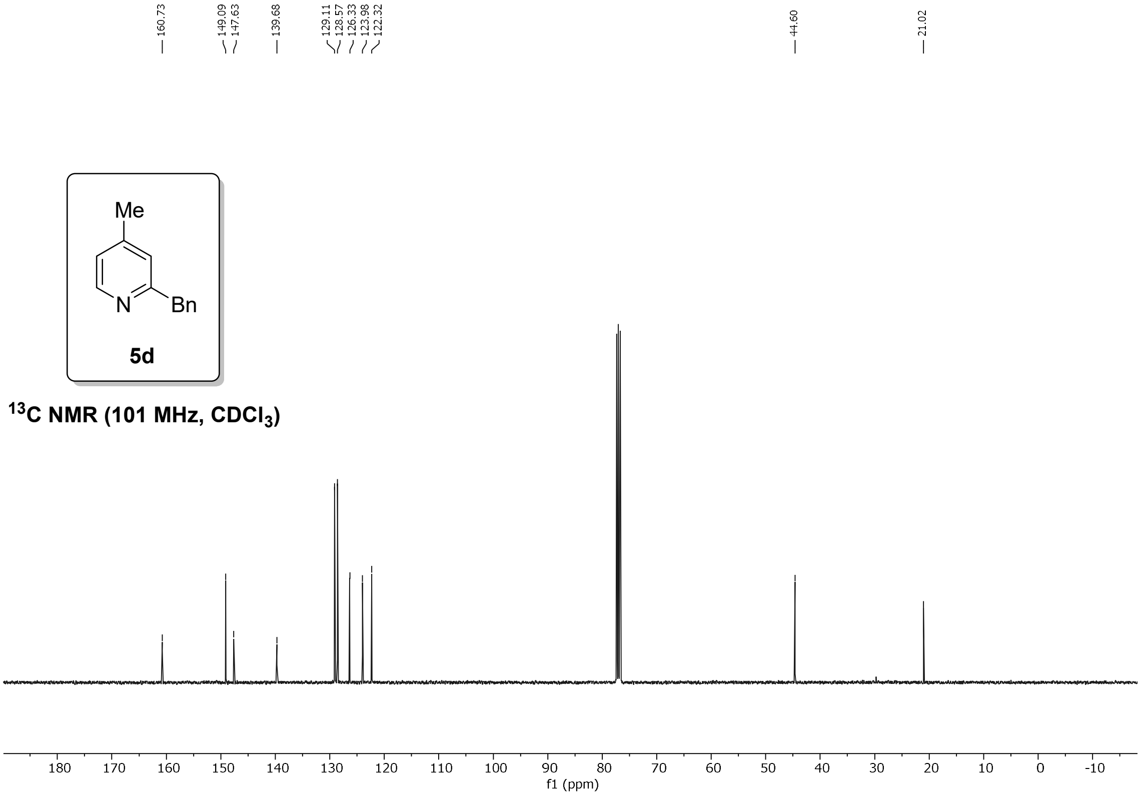

^{13}C NMR (101 MHz, $CDCl_3$)

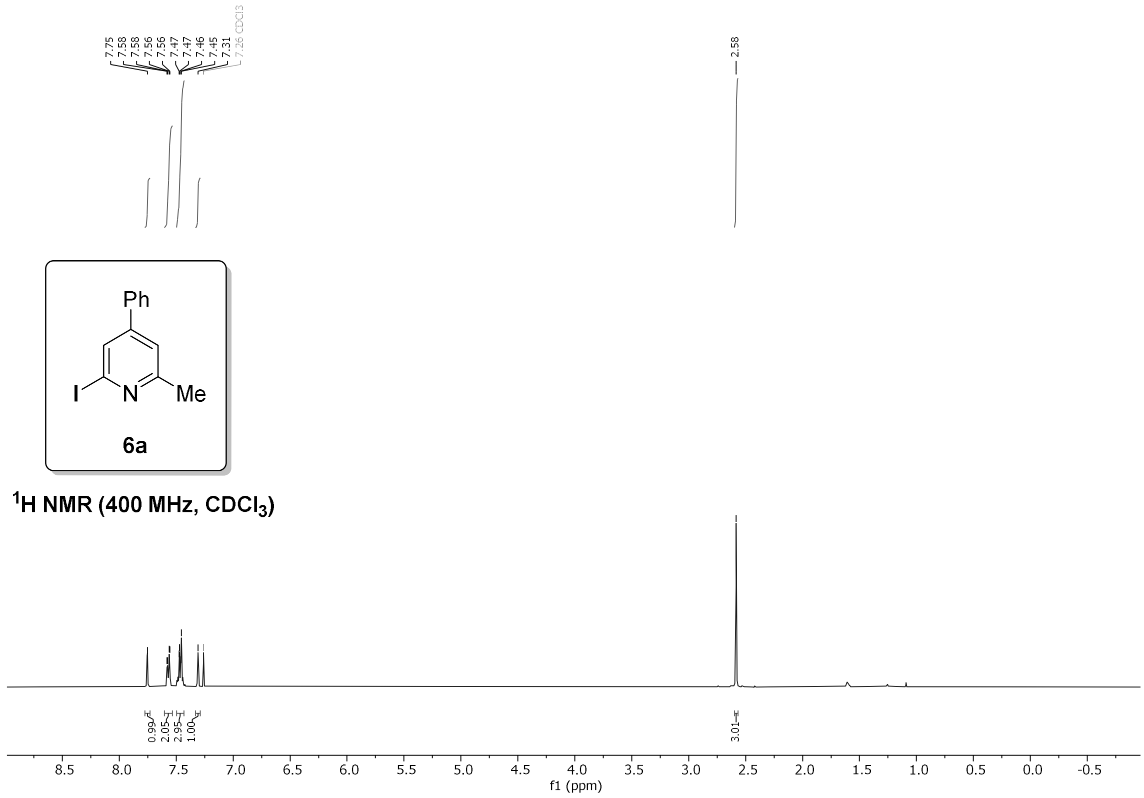

^{1}H NMR (400 MHz, $CDCl_3$)

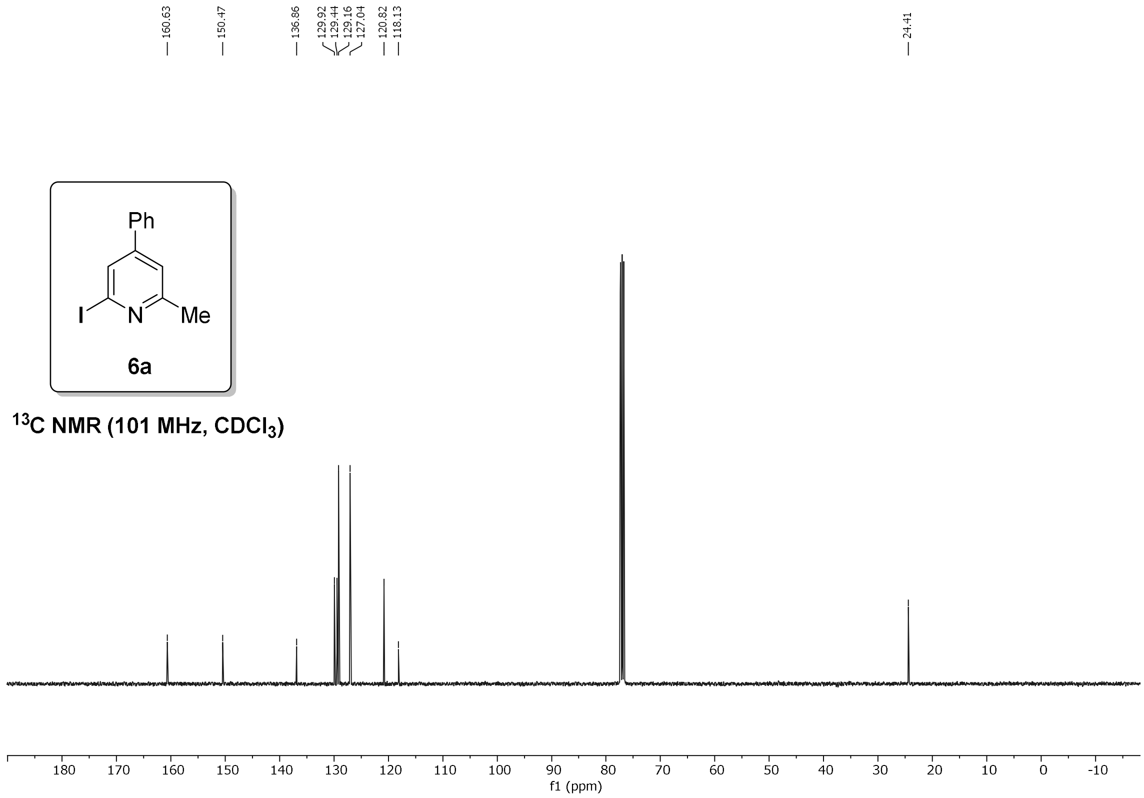

^{13}C NMR (101 MHz, $CDCl_3$)

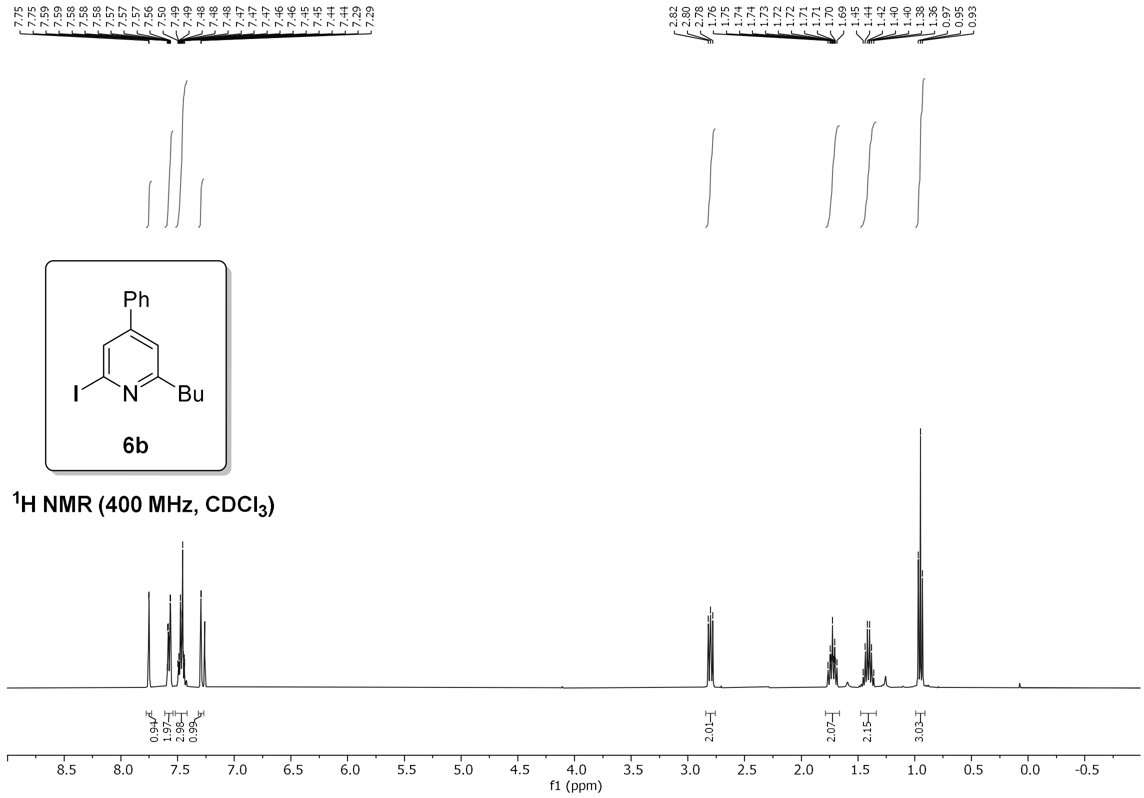
Ph
I
N
Bu
6b
1H NMR (400 MHz, CDCl3)
0.94
1.97
2.98
0.99
2.01
2.07
2.15
3.03
f1 (ppm)

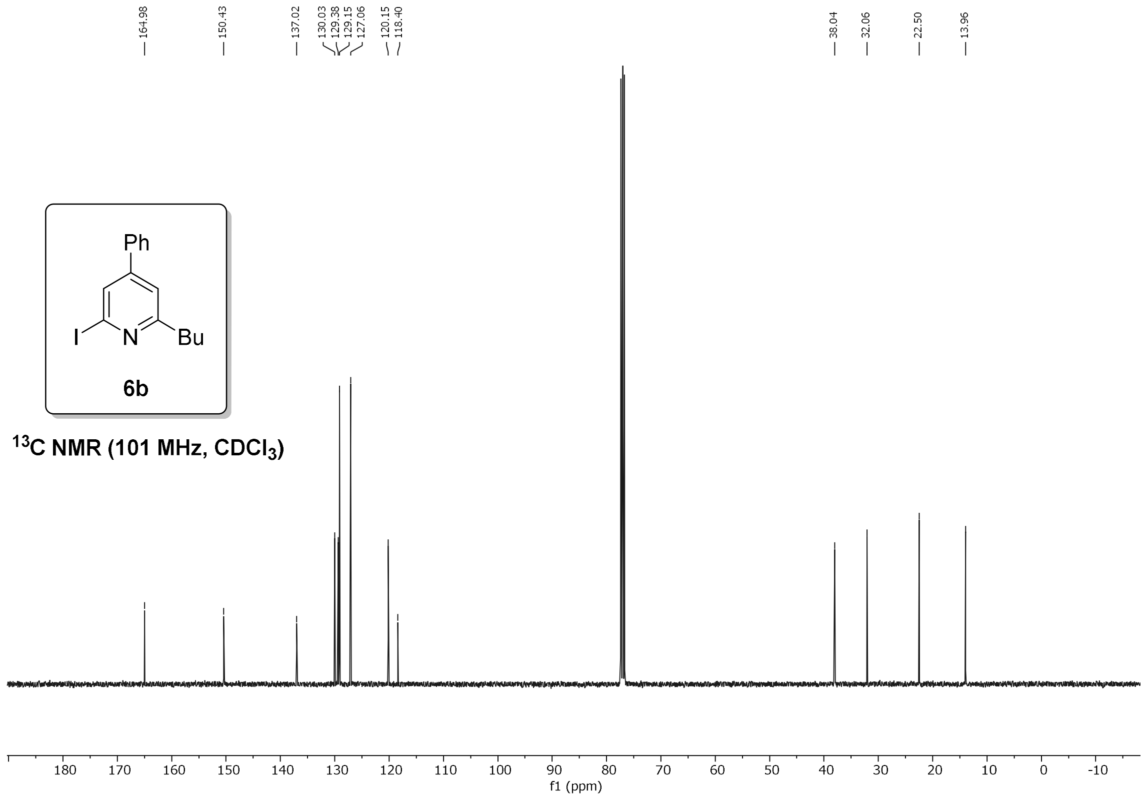
164.98
150.43
137.02
130.03
129.38
129.15
127.06
120.15
118.40
38.04
32.06
22.50
13.96
Ph
I
N
Bu
6b
13C NMR (101 MHz, CDCl3)
f1 (ppm)

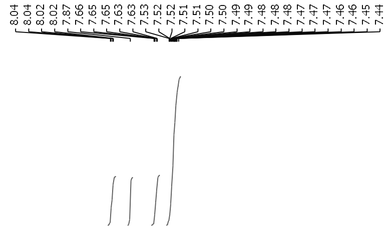

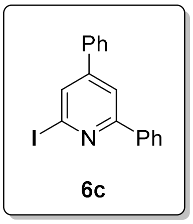

^{1}H NMR (400 MHz, $CDCl_3$)

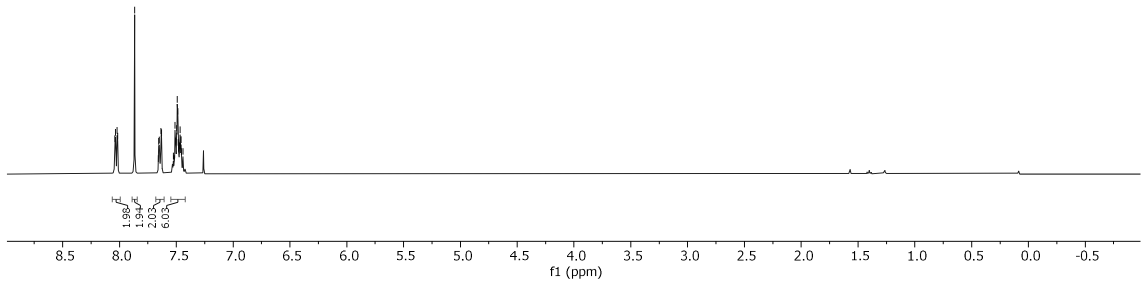

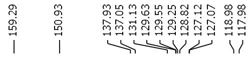

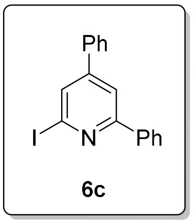

^{13}C NMR (101 MHz, $CDCl_3$)

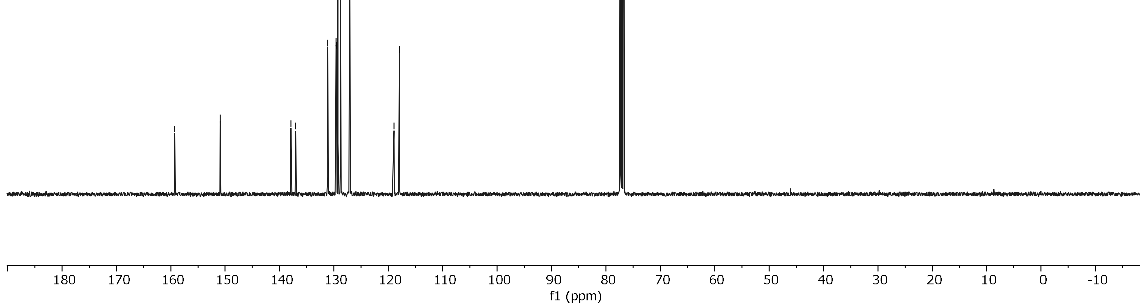

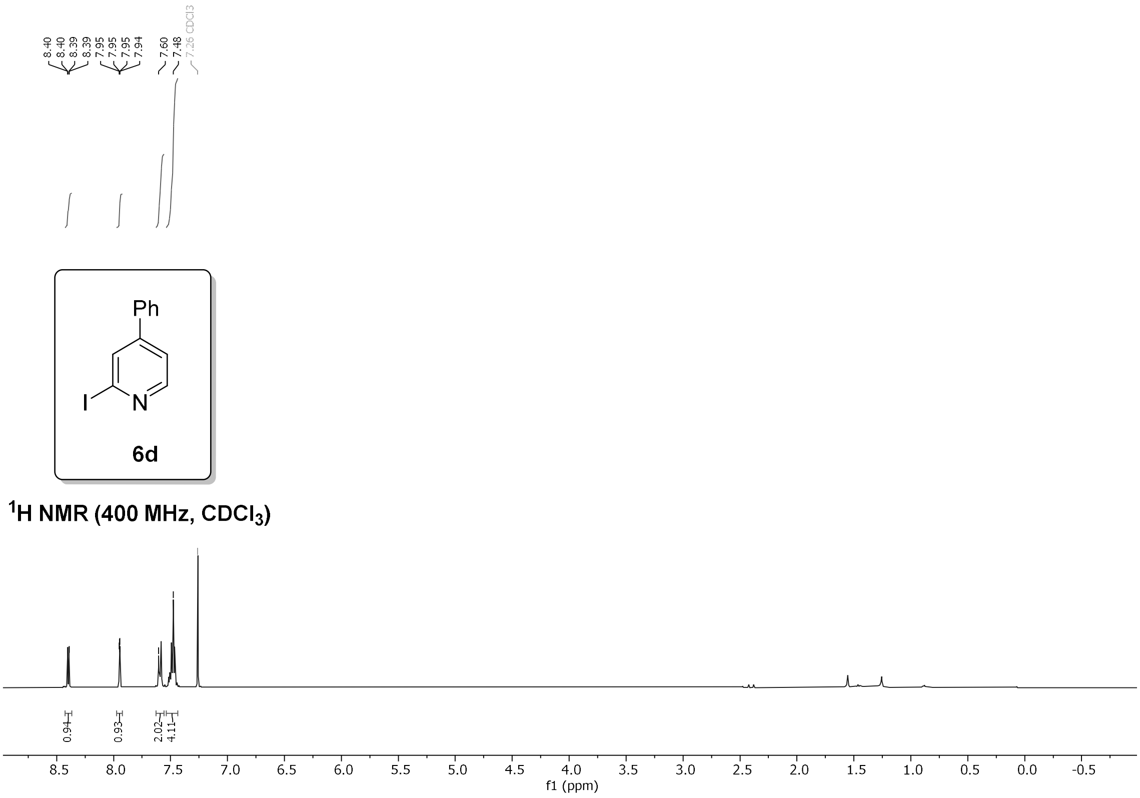

^{1}H NMR (400 MHz, $CDCl_3$)

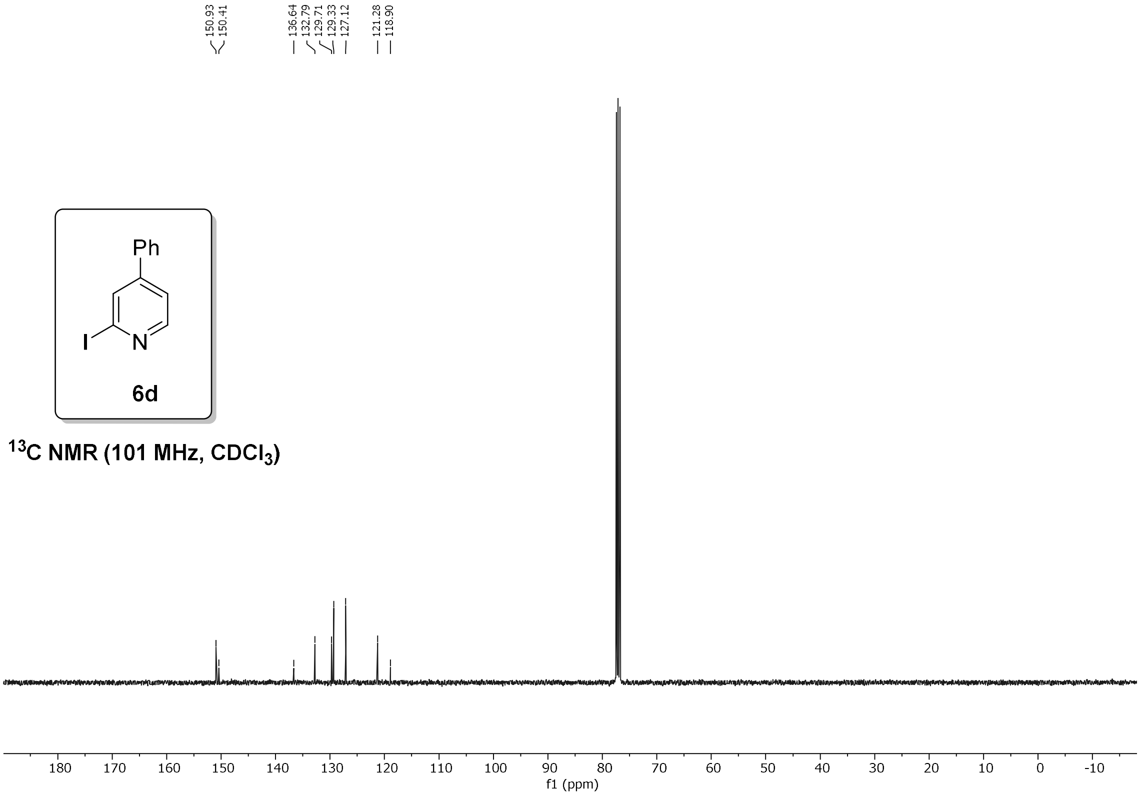

^{13}C NMR (101 MHz, $CDCl_3$)

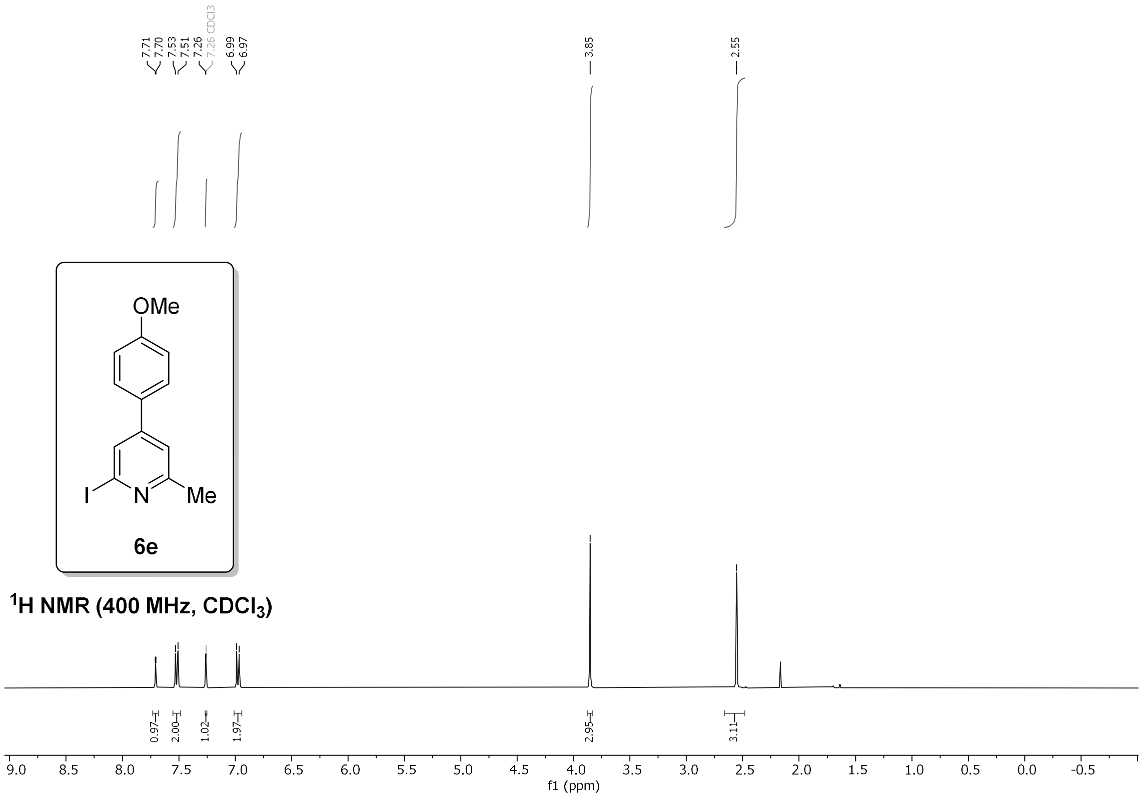

^{1}H NMR (400 MHz, $CDCl_3$)

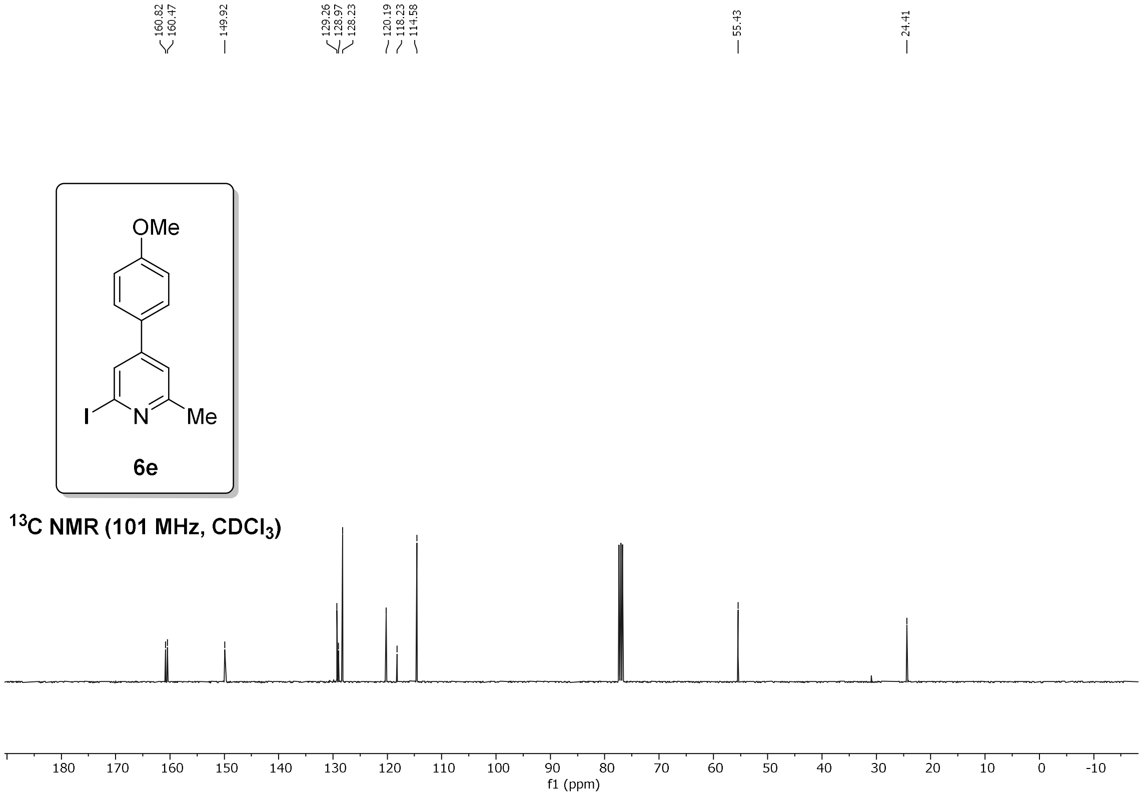

^{13}C NMR (101 MHz, $CDCl_3$)

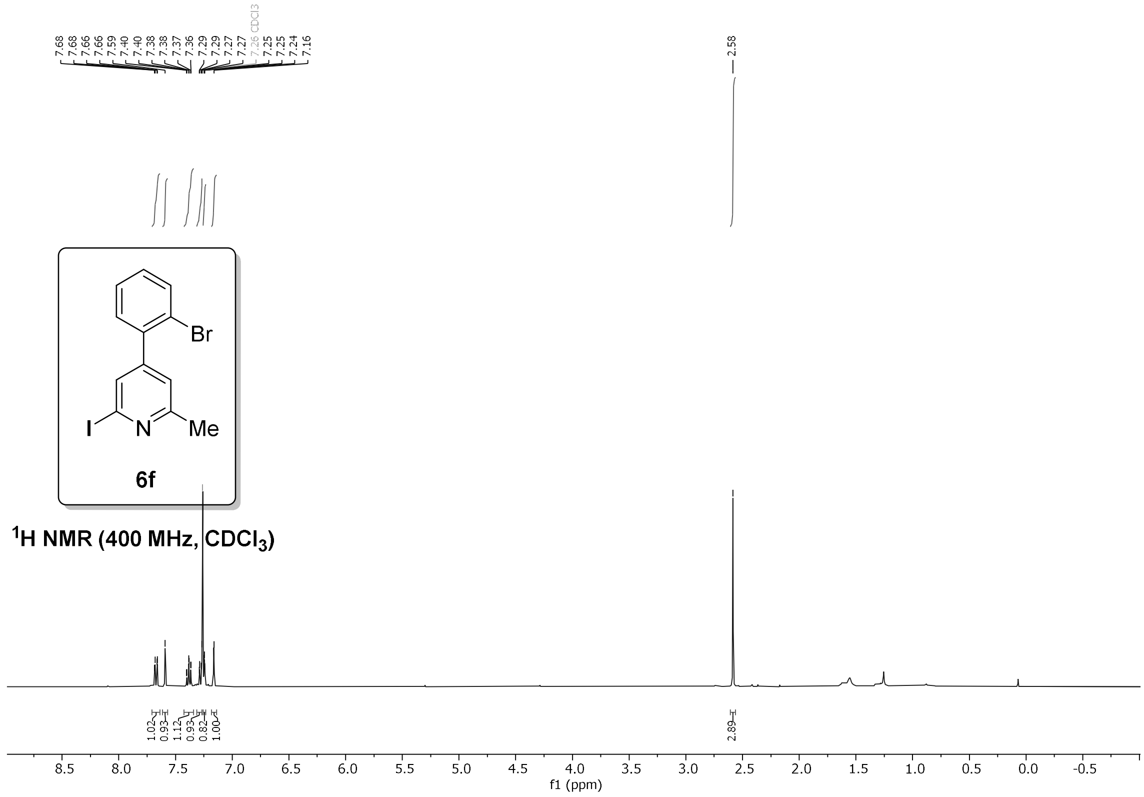
7.68 7.68 7.66 7.66 7.59 7.40 7.40 7.38 7.38 7.37 7.36 7.29 7.29 7.27 7.27 7.26 CDCl3 7.25 7.25 7.24 7.16
2.58
Br
I
N
Me
6f
^{1}H NMR (400 MHz, CDCl$_3$)
1.02 0.93 1.12 0.93 0.82 1.00
2.89
f1 (ppm)

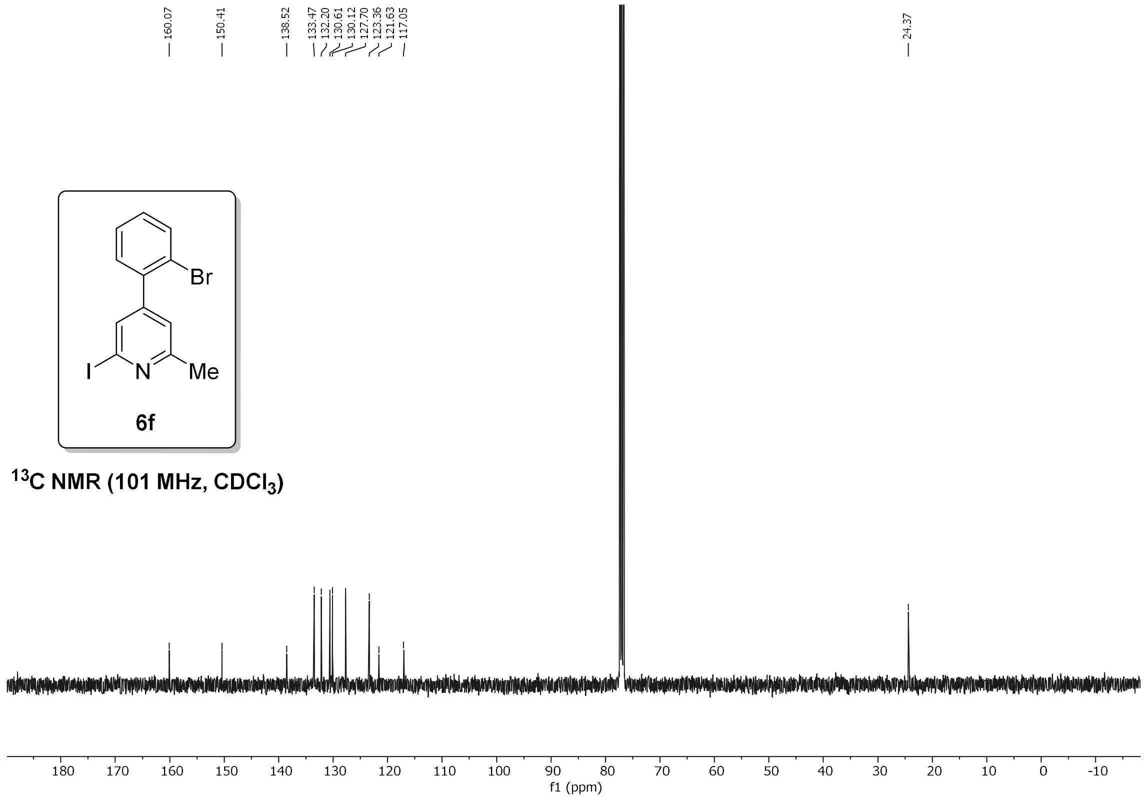
160.07
150.41
138.52
133.47 132.20 130.61 130.12 127.70 123.36 121.63 117.05
24.37
Br
I
N
Me
6f
^{13}C NMR (101 MHz, CDCl$_3$)
f1 (ppm)

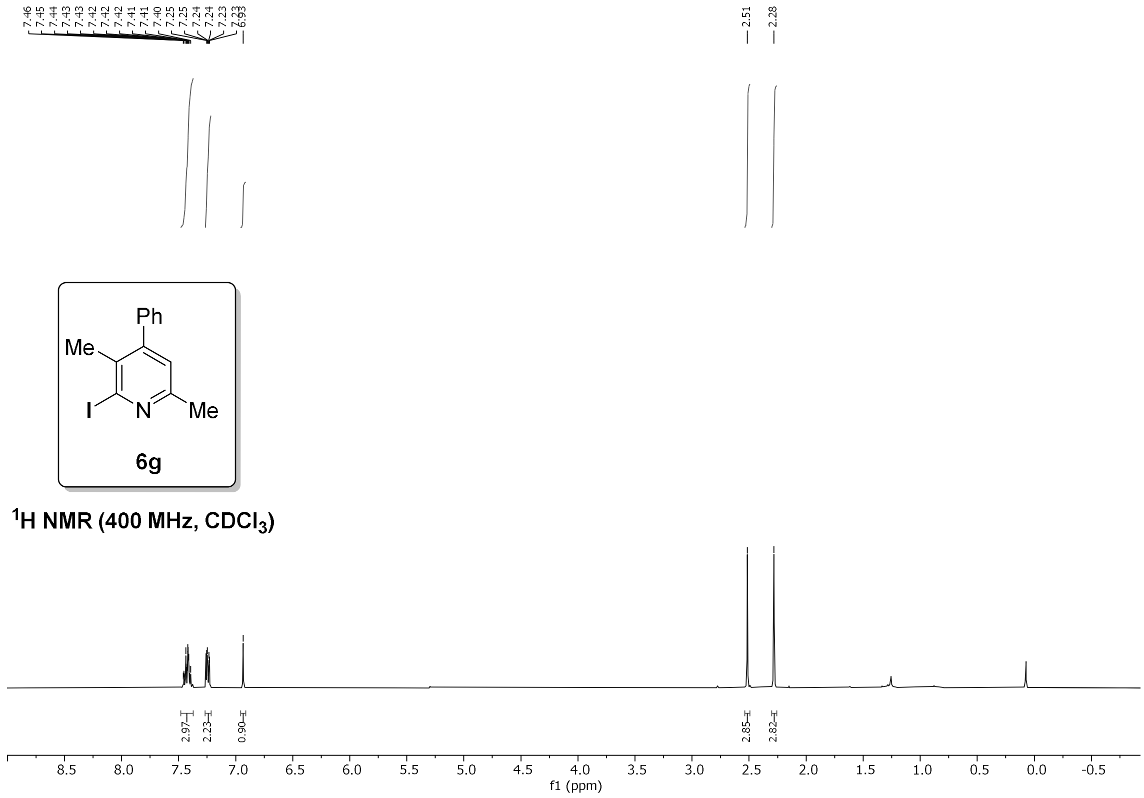

6g

^{1}H NMR (400 MHz, $CDCl_3$)

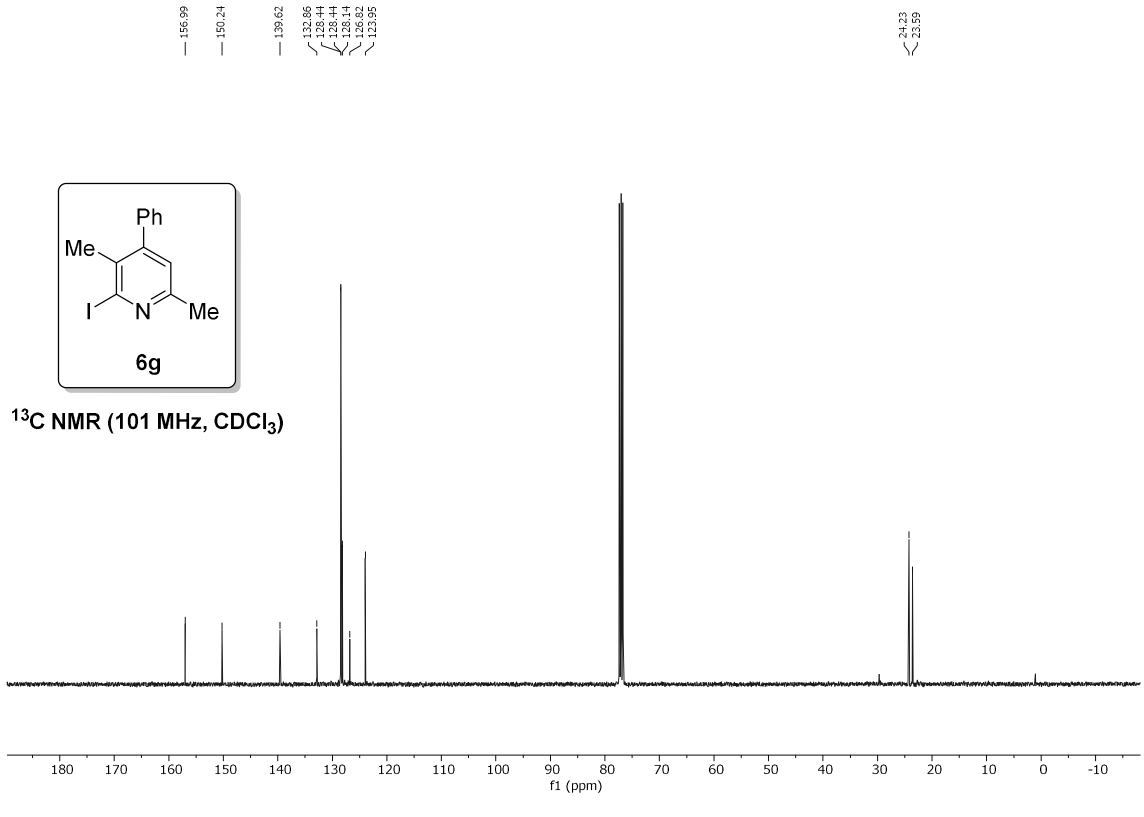

6g

^{13}C NMR (101 MHz, $CDCl_3$)

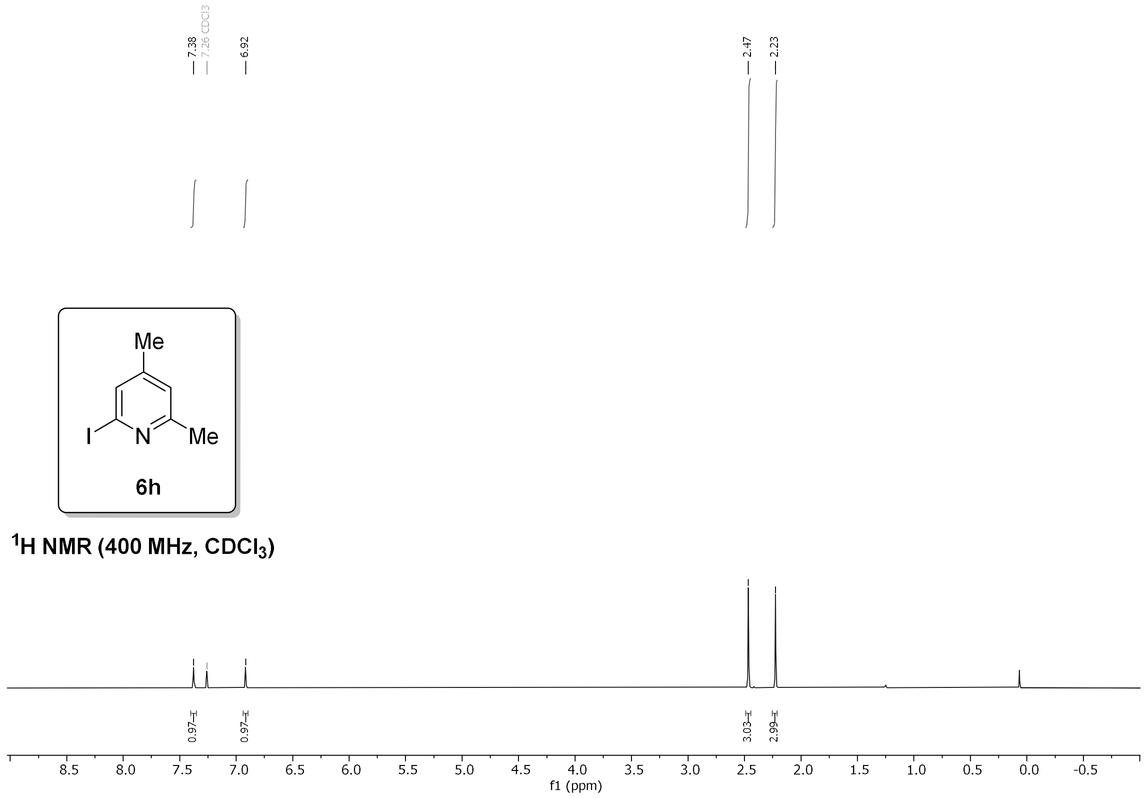
7.38
7.26 CDCl3
6.92
2.47
2.23
Me
I
N
Me
6h
^{1}H NMR (400 MHz, CDCl$_3$)
0.97
0.97
3.03
2.99
8.5 8.0 7.5 7.0 6.5 6.0 5.5 5.0 4.5 4.0 3.5 3.0 2.5 2.0 1.5 1.0 0.5 0.0 -0.5
f1 (ppm)

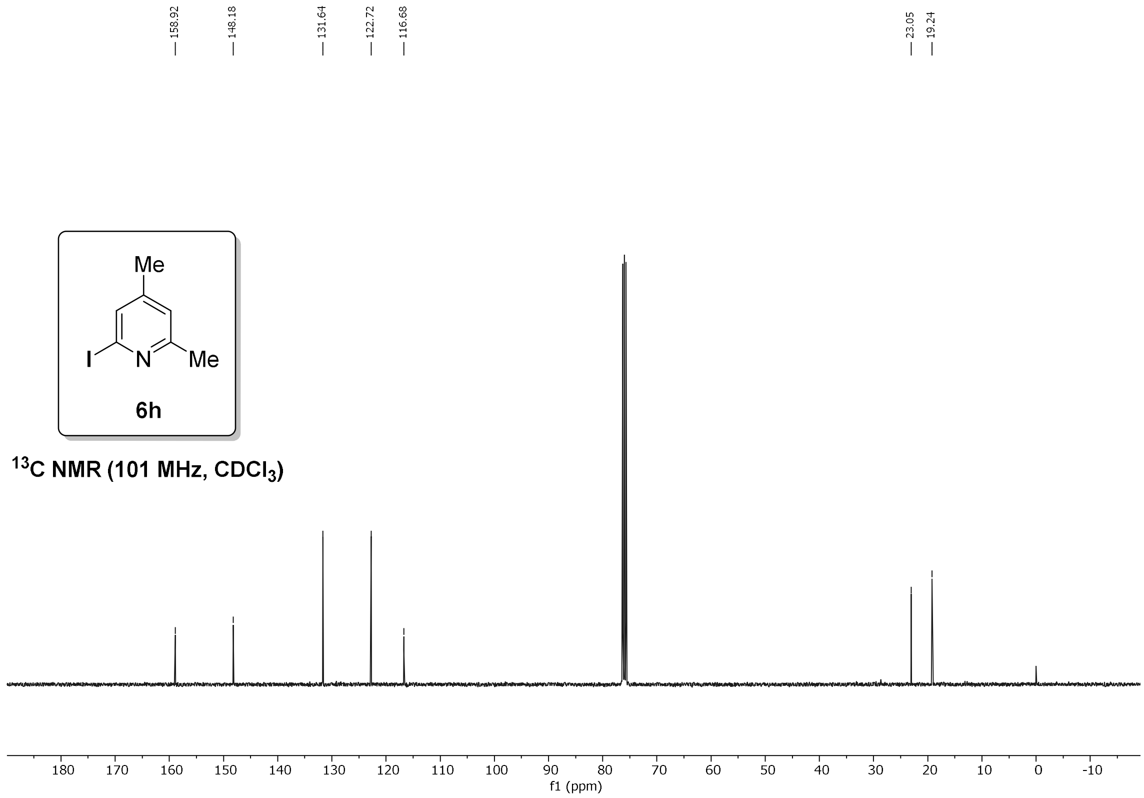
158.92
148.18
131.64
122.72
116.68
23.05
19.24
Me
I
N
Me
6h
^{13}C NMR (101 MHz, CDCl$_3$)
180 170 160 150 140 130 120 110 100 90 80 70 60 50 40 30 20 10 0 -10
f1 (ppm)

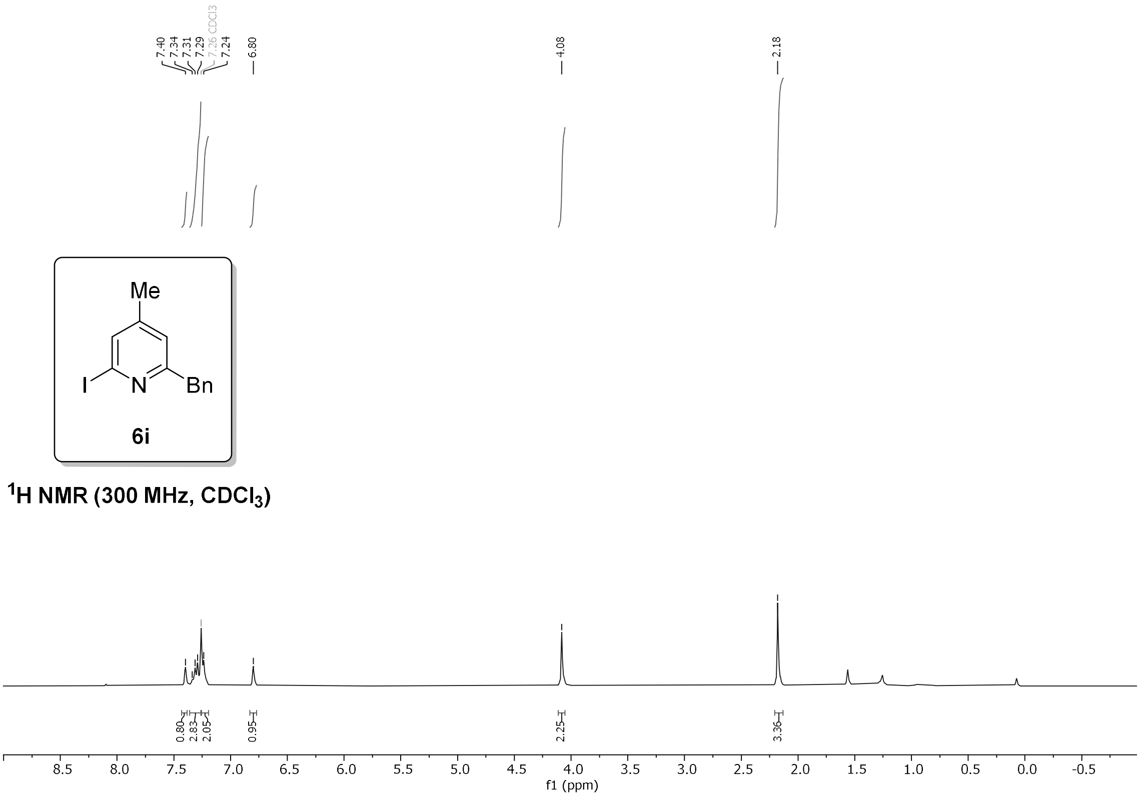

6i

^{1}H NMR (300 MHz, $CDCl_3$)

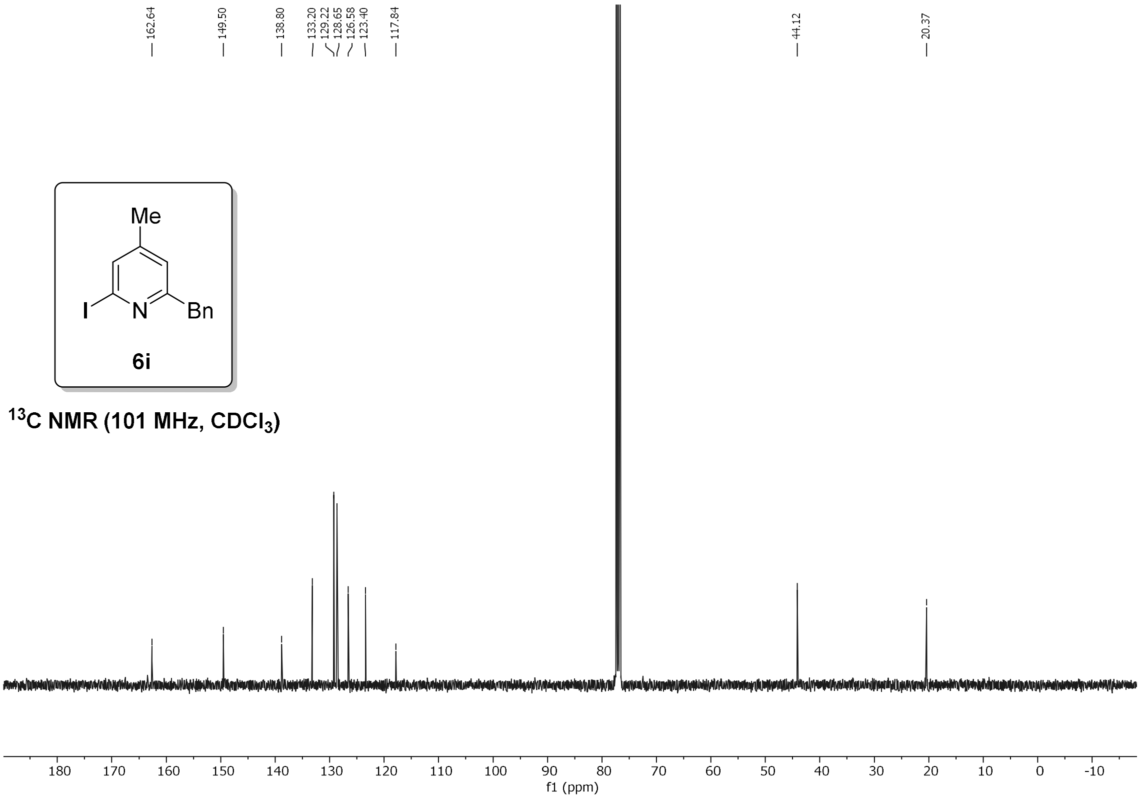

6i

^{13}C NMR (101 MHz, $CDCl_3$)

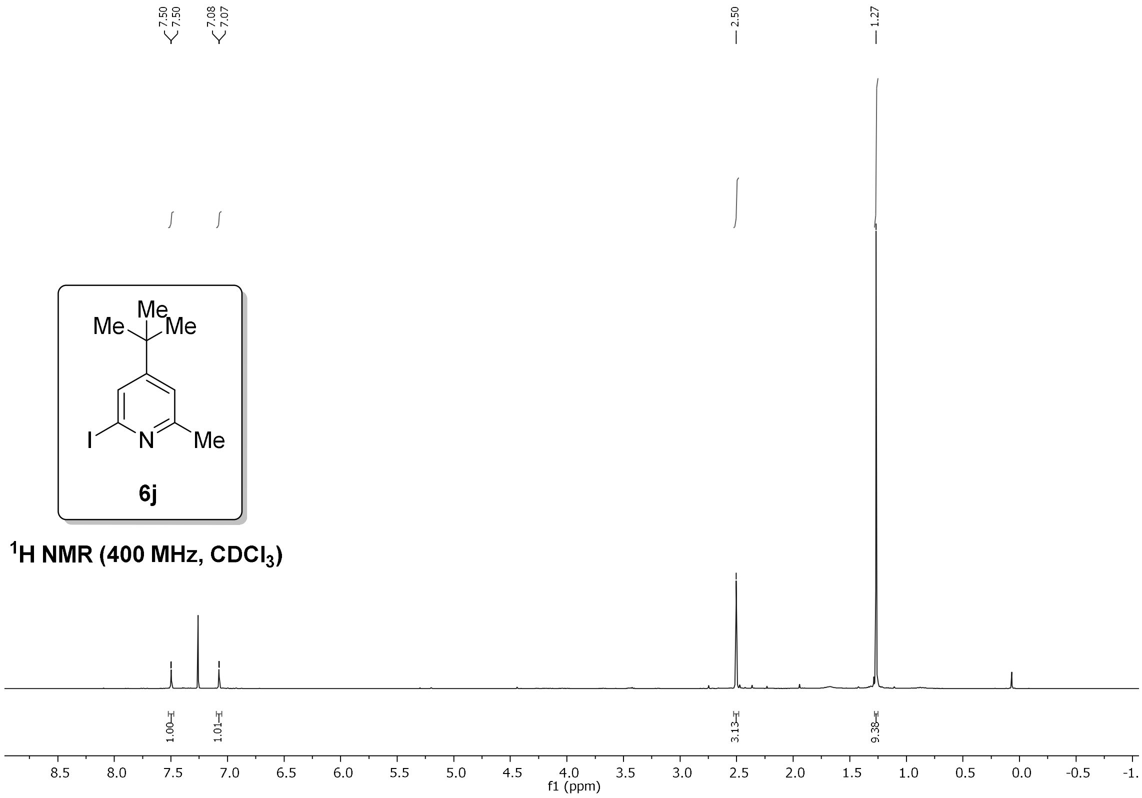

^{1}H NMR (400 MHz, $CDCl_3$)

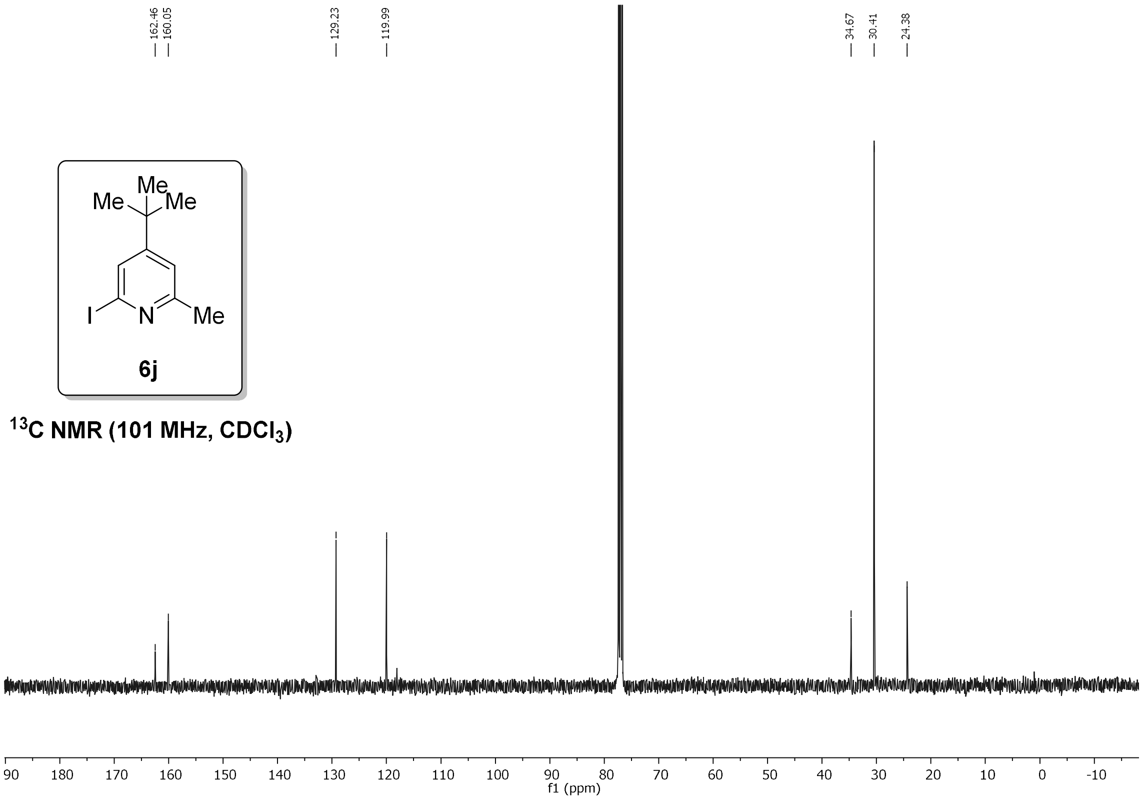

^{13}C NMR (101 MHz, $CDCl_3$)

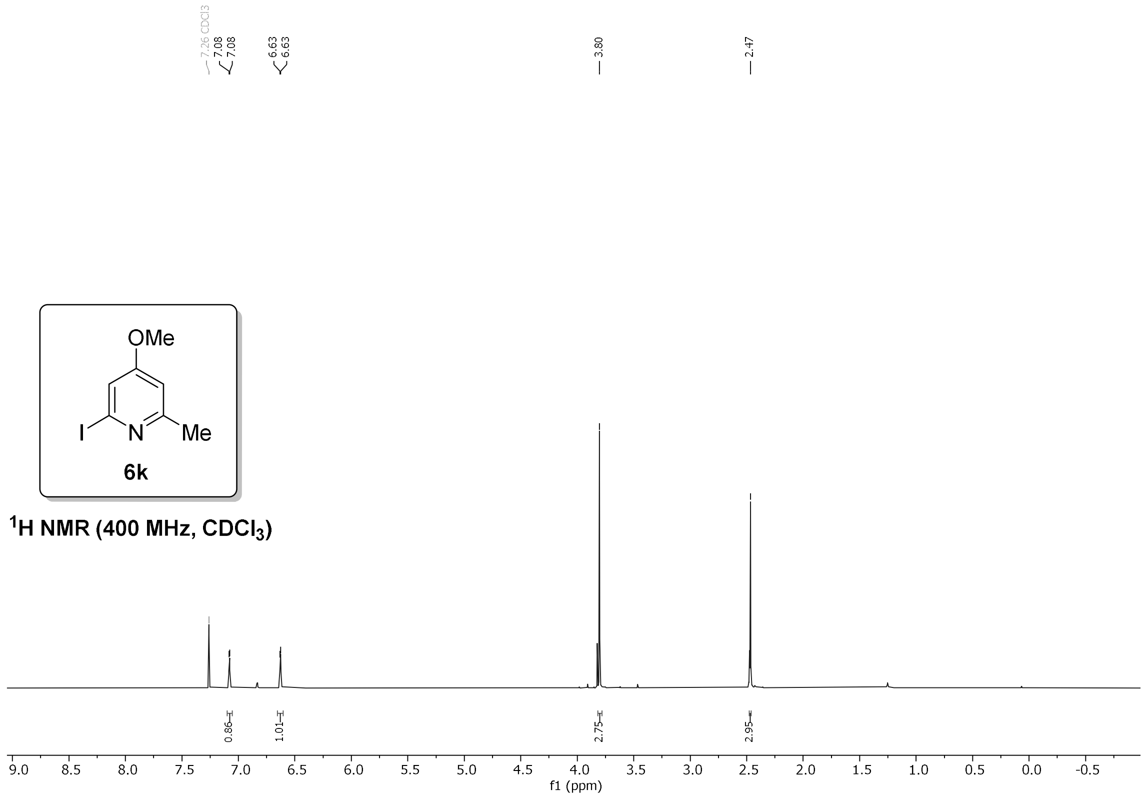

^{1}H NMR (400 MHz, $CDCl_3$)

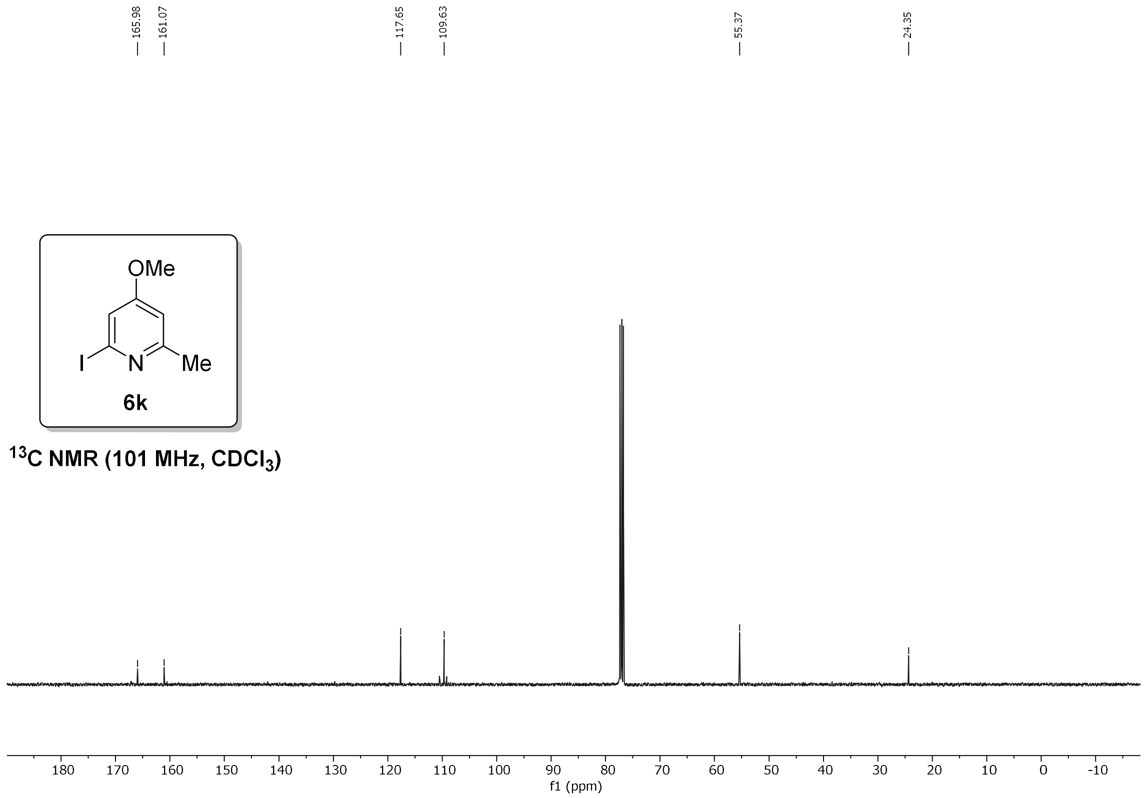

^{13}C NMR (101 MHz, $CDCl_3$)

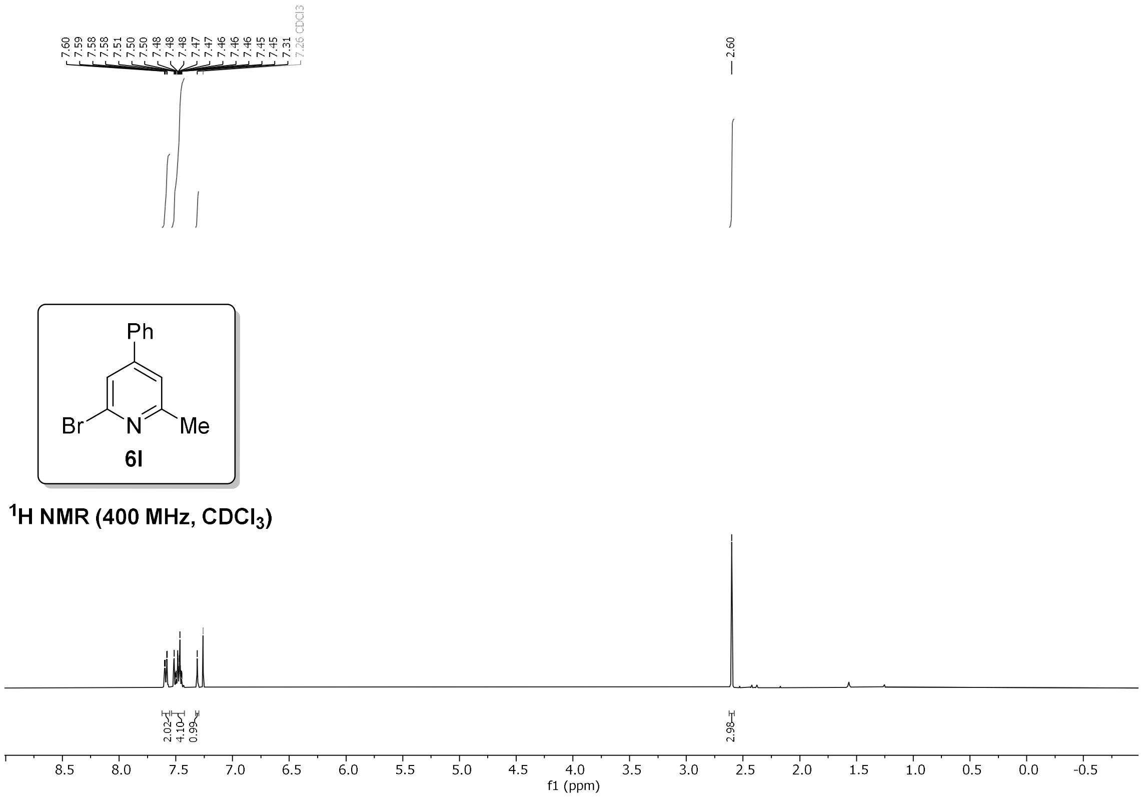

^{1}H NMR (400 MHz, CDCl$_3$)

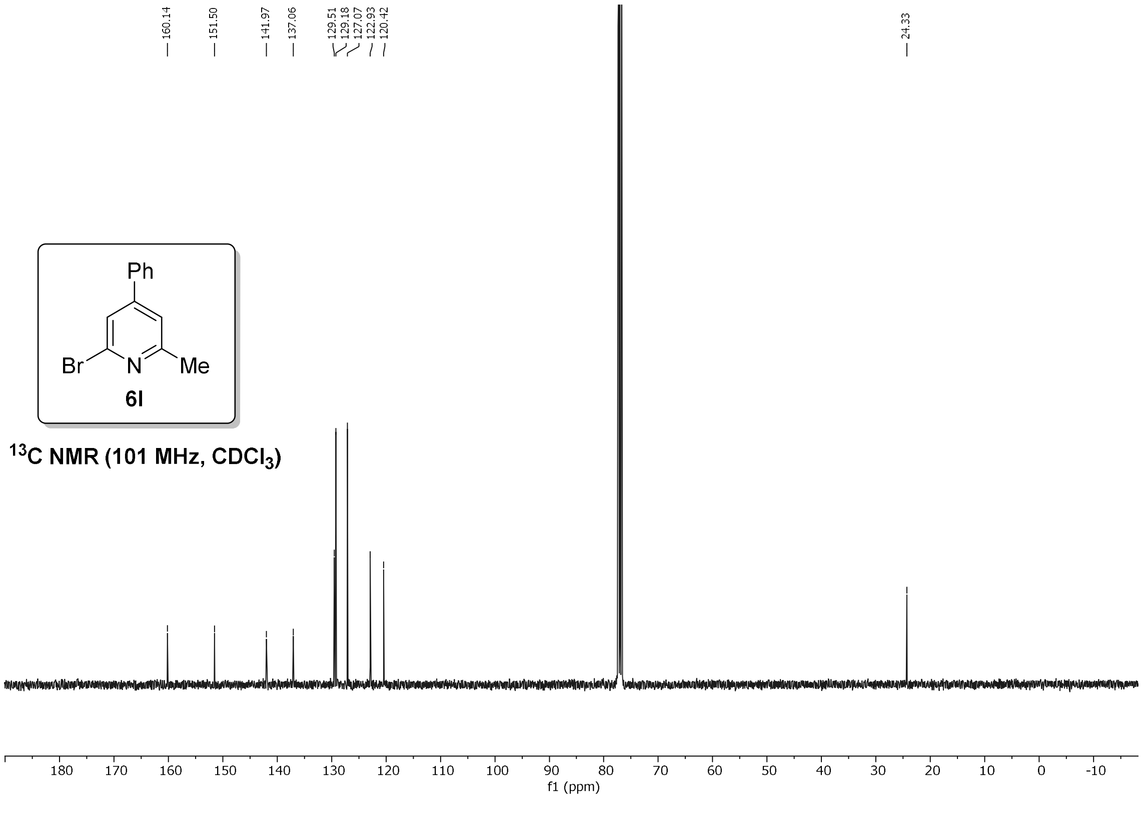

^{13}C NMR (101 MHz, CDCl$_3$)

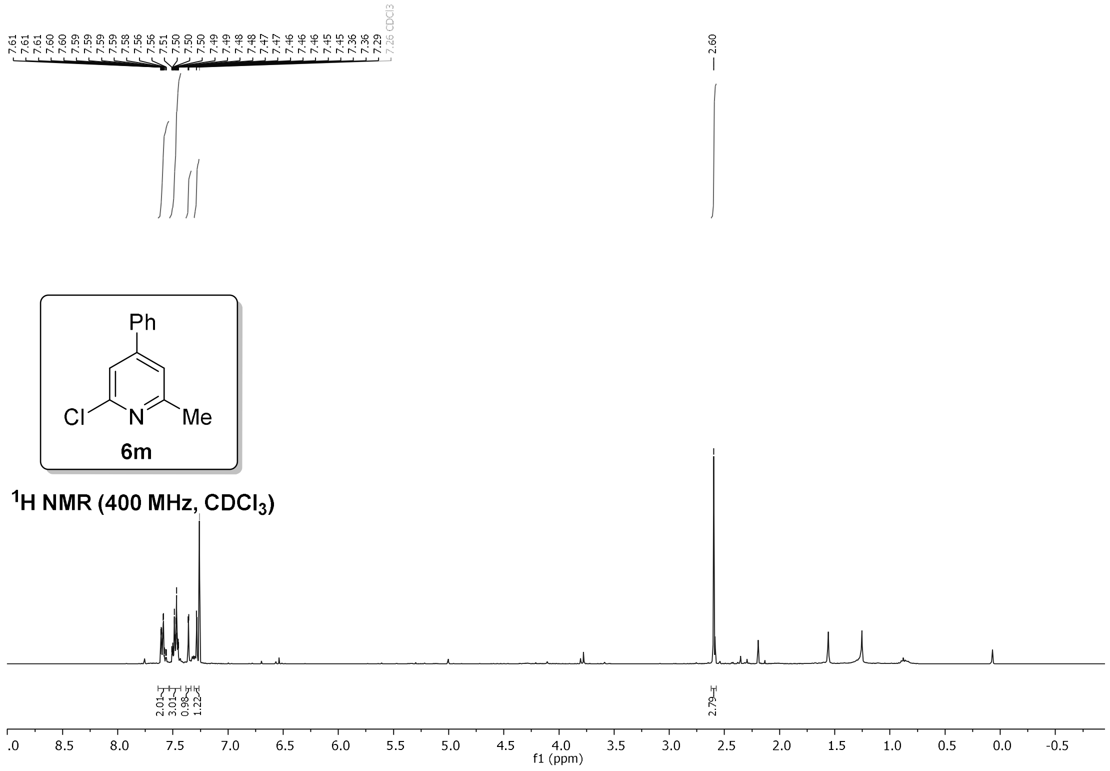
7.61 7.61 7.61 7.60 7.59 7.59 7.59 7.59 7.58 7.56 7.56 7.51 7.50 7.50 7.50 7.49 7.49 7.48 7.48 7.47 7.47 7.46 7.46 7.46 7.45 7.45 7.36 7.36 7.29 7.26 CDCl3
2.60
Ph
Cl
N
Me
6m
^{1}H NMR (400 MHz, $CDCl_3$)
2.01 3.01 0.98 1.22
2.79
f1 (ppm)

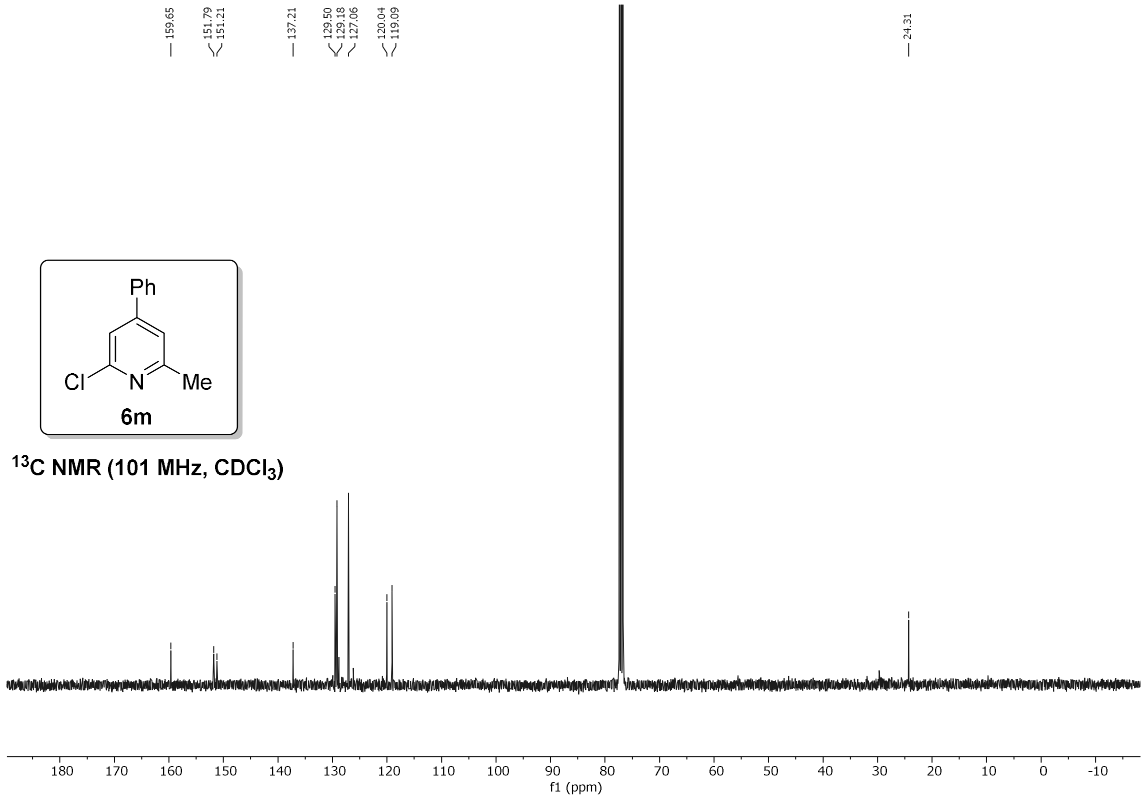
159.65
151.79 151.21
137.21
129.50 129.18 127.06
120.04 119.09
24.31
Ph
Cl
N
Me
6m
^{13}C NMR (101 MHz, $CDCl_3$)
f1 (ppm)

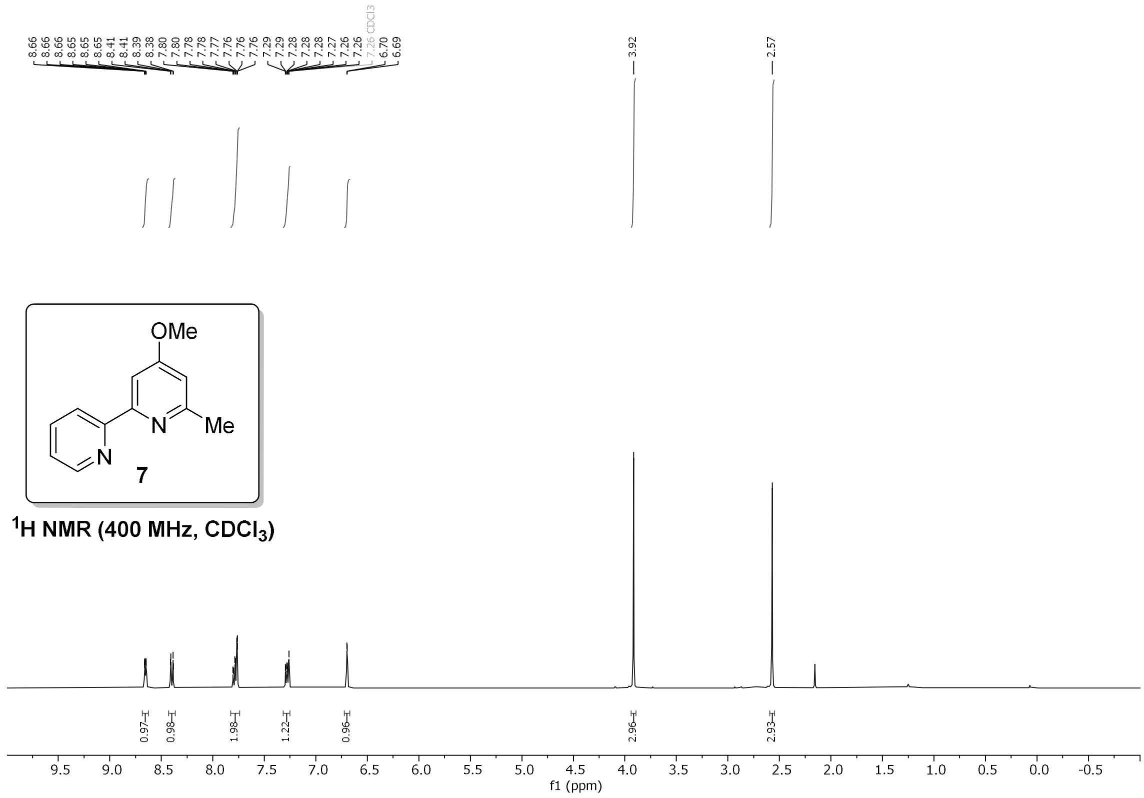
8.66 8.66 8.66 8.65 8.65 8.65 8.41 8.41 8.39 8.38 7.80 7.78 7.78 7.77 7.76 7.76 7.76 7.29 7.29 7.28 7.28 7.28 7.27 7.26 7.26 7.26 CDCl3 6.70 6.69
3.92
2.57
OMe
N
Me
N
7
1H NMR (400 MHz, CDCl3)
0.97 0.98 1.98 1.22 0.96 2.96 2.93
9.5 9.0 8.5 8.0 7.5 7.0 6.5 6.0 5.5 5.0 4.5 4.0 3.5 3.0 2.5 2.0 1.5 1.0 0.5 0.0 -0.5
f1 (ppm)

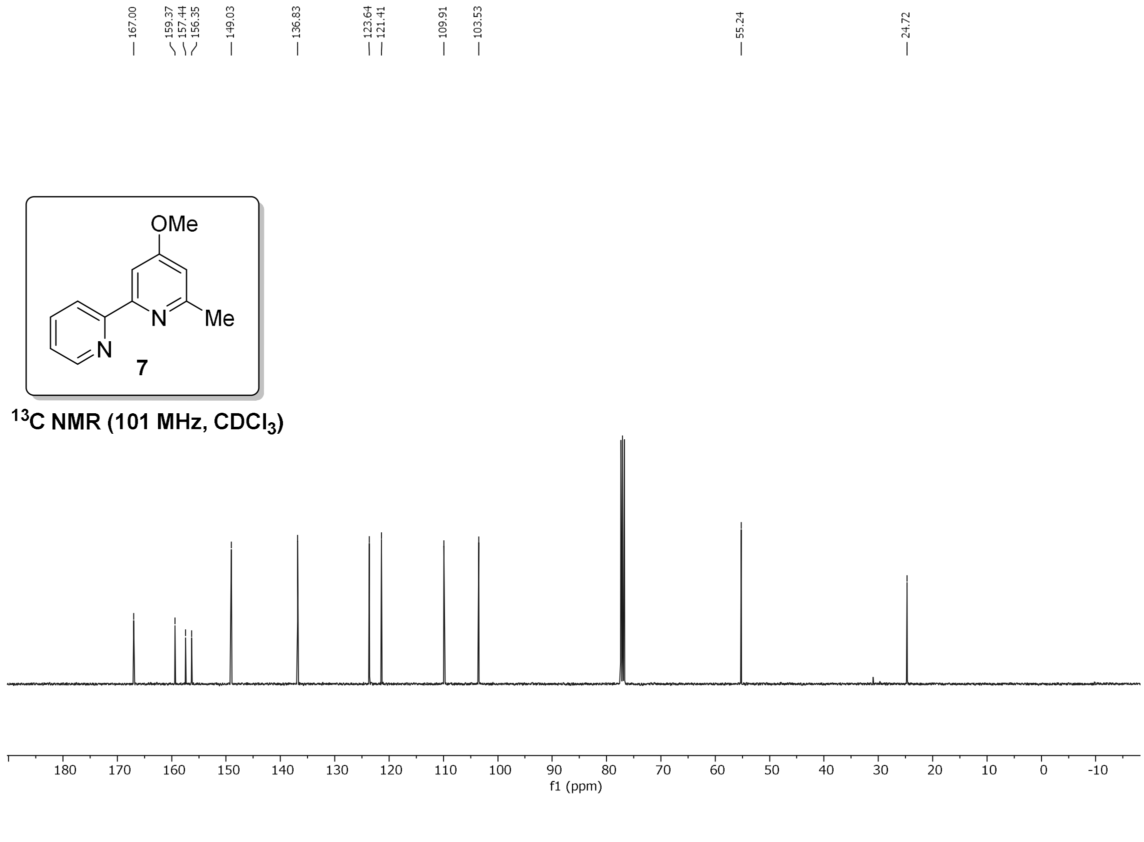
167.00 159.37 157.44 156.35 149.03 136.83 123.64 121.41 109.91 103.53 55.24 24.72
OMe
N
Me
N
7
13C NMR (101 MHz, CDCl3)
180 170 160 150 140 130 120 110 100 90 80 70 60 50 40 30 20 10 0 -10
f1 (ppm)

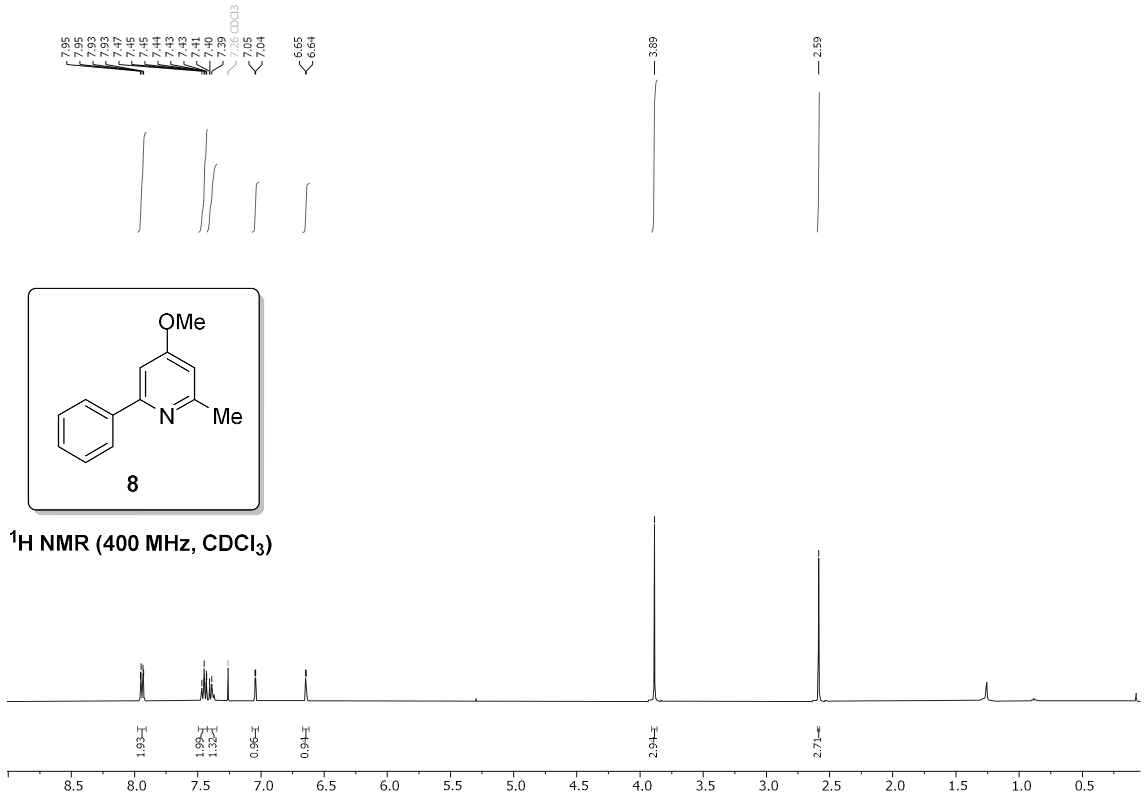

^{1}H NMR (400 MHz, $CDCl_3$)

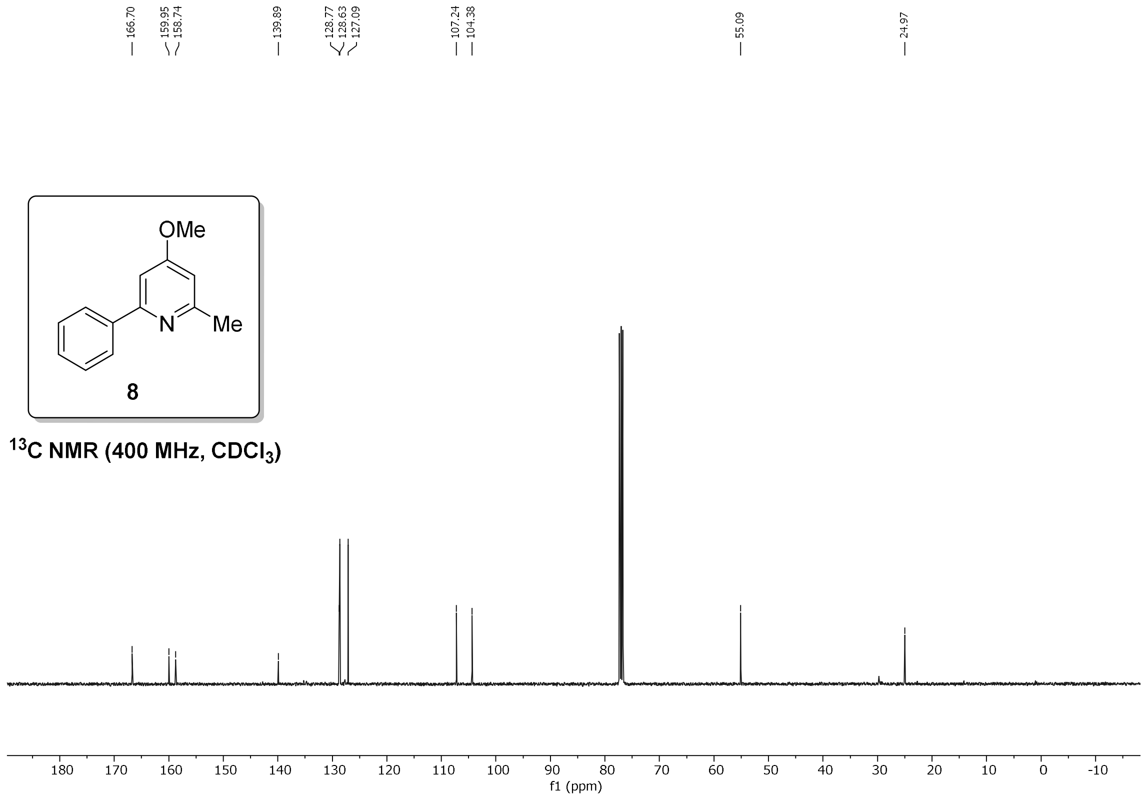

^{13}C NMR (400 MHz, $CDCl_3$)

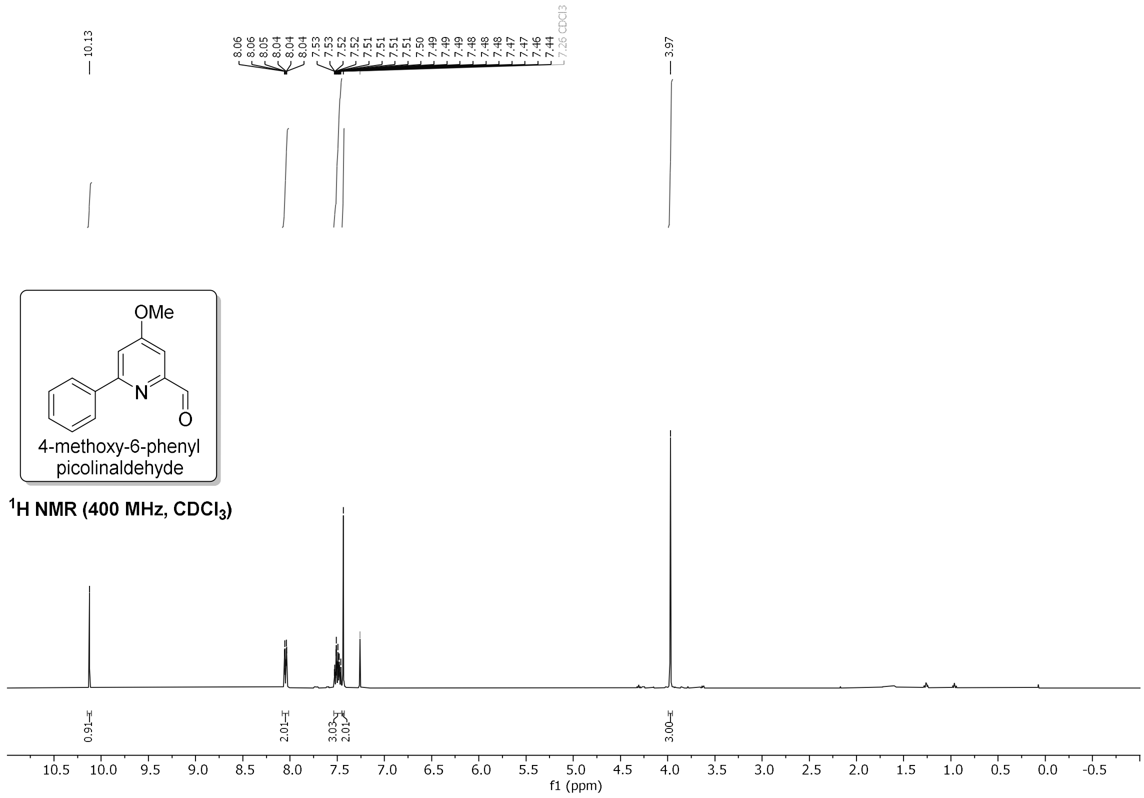

OMe
N
O
4-methoxy-6-phenyl picolinaldehyde
^{1}H NMR (400 MHz, CDCl$_3$)
10.13
8.06 8.06 8.05 8.04 8.04 8.04
7.53 7.53 7.52 7.52 7.51 7.51 7.51 7.50 7.49 7.49 7.49 7.48 7.48 7.48 7.47 7.46 7.44
7.26 CDCl3
3.97
0.91
2.01
3.03
2.01
3.00
f1 (ppm)

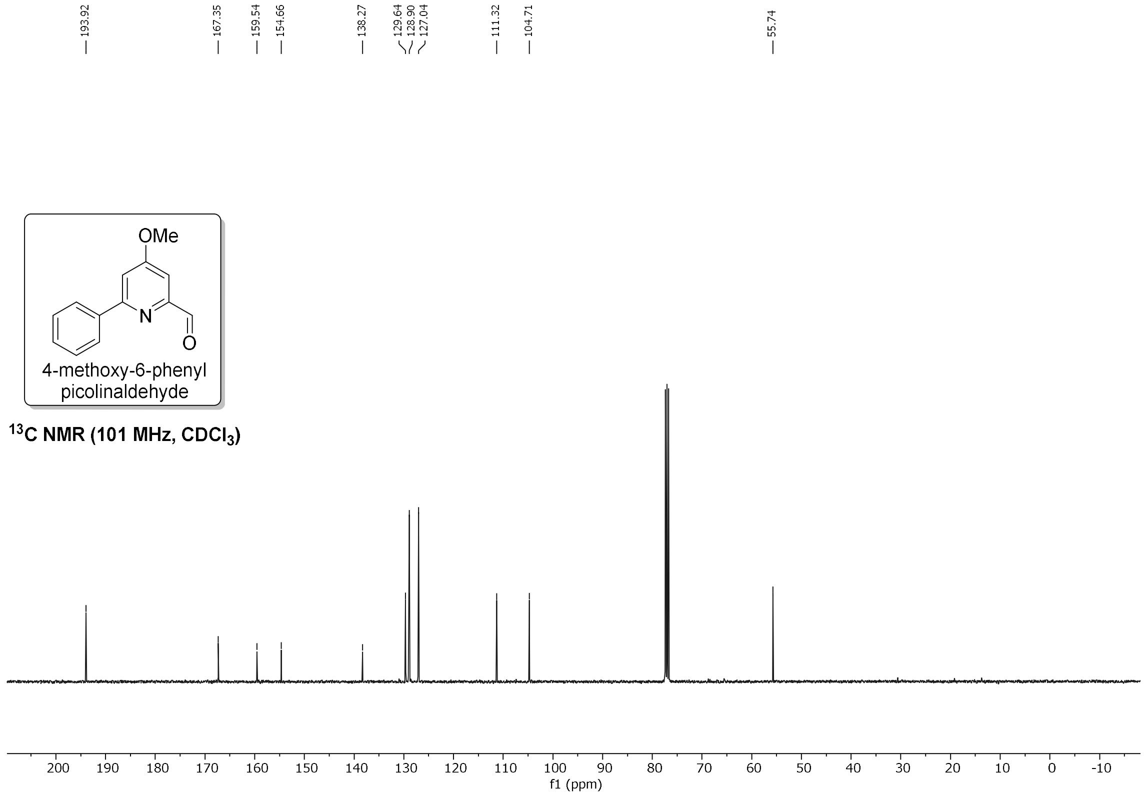

OMe
N
O
4-methoxy-6-phenyl picolinaldehyde
^{13}C NMR (101 MHz, CDCl$_3$)
193.92
167.35
159.54
154.66
138.27
129.64
128.90
127.04
111.32
104.71
55.74
f1 (ppm)

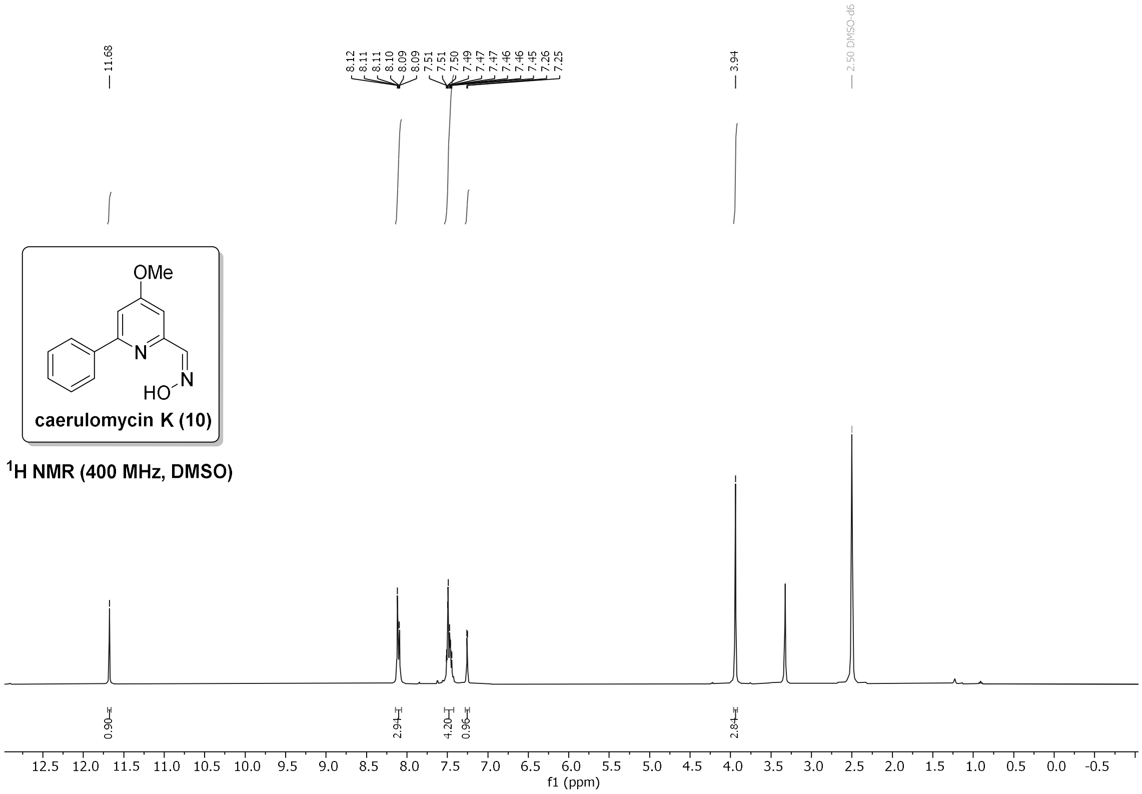

^{1}H NMR (400 MHz, DMSO)

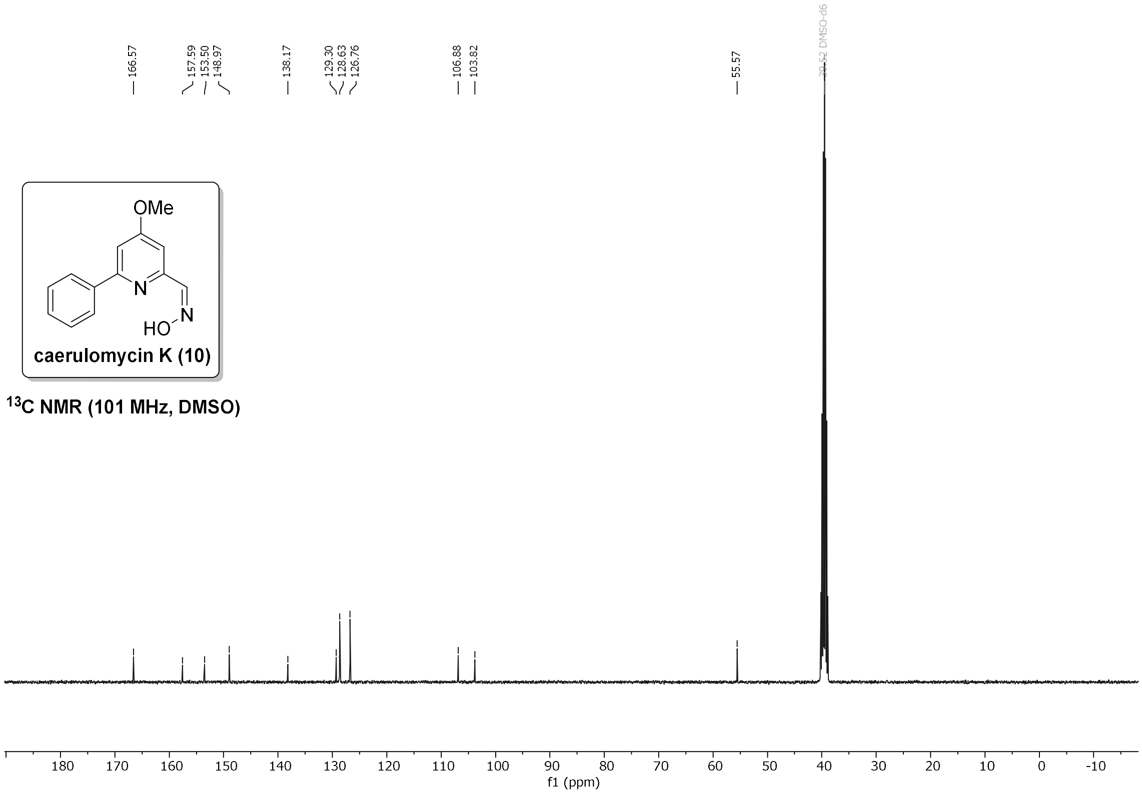

^{13}C NMR (101 MHz, DMSO)
